# Atomically precise copper cluster scintillator

**DOI:** 10.1039/d6sc06463a

**Published:** 2026-09-16

**Authors:** Yuan-Yuan Li, Jin-Le Duan, Yu-Jin Kong, Qiu-Chen Peng, Kai Li, Shuang-Quan Zang

**Affiliations:** a School of Chemistry and Chemical Engineering, Henan University of Technology Zhengzhou Henan 450001 China yuanyuanli@haut.edu.cn; b Key Laboratory of Special Environmental Functional Materials (Zhengzhou University), Ministry of Education, Tianjian Laboratory of Advanced Biomedical Sciences, Henan Key Laboratory of Crystalline Molecular Functional Materials, College of Chemistry, Zhengzhou University Zhengzhou 450001 China likai@zzu.edu.cn zangsqzg@zzu.edu.cn

## Abstract

As a kind of newly developed scintillator, copper cluster scintillators have garnered considerable attention due to their atomically precise structures and multiple advantages such as low toxicity, facile synthesis and outstanding X-ray excited luminescence (XEL) properties. The copper cluster scintillators have been successfully applied in various fields including low-dose radiation detection, X-ray imaging, and the monitoring of α/β particles, suggesting that they are a highly active research field. This review focuses on recent advancements in copper cluster scintillators and comprehensively summarize the luminescence mechanisms and design strategies employed to tailor the XEL of copper clusters, copper halide clusters and copper cluster-based assembly materials. This review also presents the novel applications derived from the as-obtained copper cluster scintillators, along with the underlying structure–functionality correlations.

## Abstract 2

Copper cluster scintillators have garnered considerable attention due to their atomically precise structures and multiple advantages such as low toxicity, facile synthesis and outstanding X-ray excited luminescence (XEL) properties.

## Graphical abstract

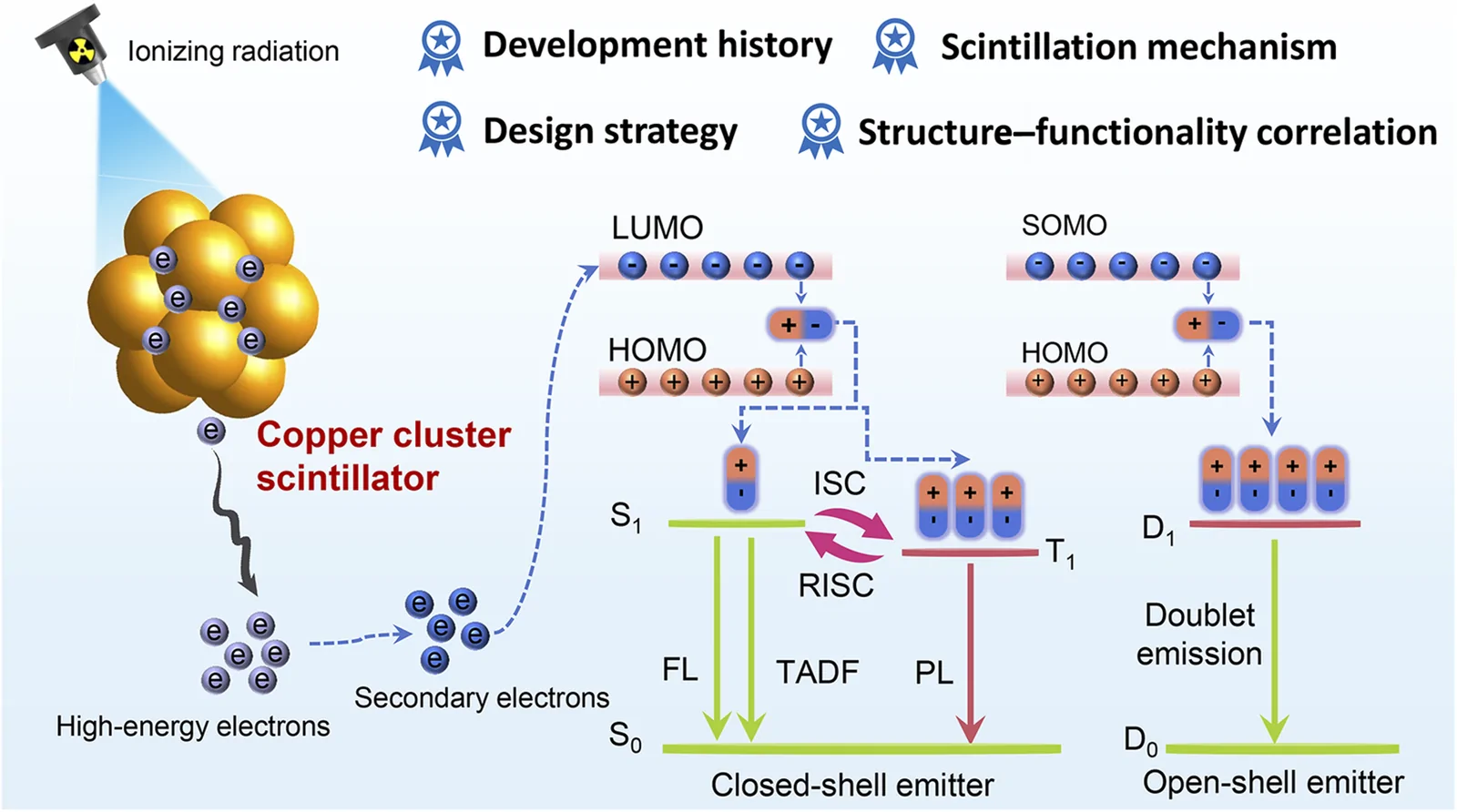

## 1. Introduction

Copper clusters are a kind of important coinage metal cluster; their atomically precise structure and lower cost compared with silver clusters and gold clusters endow them with broad and promising application prospects, including luminescent dyes in bioimaging, catalysts in organic synthesis, and chemical sensors for the detection of various target analytes.^1–4^ In recent years, the exceptional X-ray excited luminescence (XEL) properties of copper clusters have increasingly attracted research interest, leading to the successive reporting of numerous copper cluster-based scintillators. As an emerging class of scintillators, copper clusters not only possess atomically precise structures with high designability but also offer multiple advantages such as low toxicity, facile synthesis, low cost and adjustable luminescence performance.^5,6^ Copper cluster scintillators have been successfully applied in diverse fields including low-dose radiation detection, X-ray imaging, and the monitoring of α/β particles.

Since the first report in 2017 on XEL properties of a copper iodide cluster, more than 50 publications have focused on the development of copper cluster scintillators (most within the past three years), highlighting this area as a highly active and rapidly advancing research frontier. Nevertheless, there remains no comprehensive review that systematically summarizes the luminescence mechanisms and design strategies of copper cluster scintillators, which are crucial for guiding the development of new high-performance scintillating materials. Therefore, this work presents a comprehensive overview of copper cluster scintillators. We discuss their historical development, underlying scintillation mechanisms, and rational design strategies. Furthermore, the advantages and challenges associated with the use of copper clusters as scintillating materials are analysed. Finally, we summarize the practical applications that have already been realized using copper cluster scintillators.

## 2. Development history of copper cluster scintillators

The first study on XEL properties of a copper cluster was reported in 2017 by Kirakci's team.^7^ They synthesized and characterized three copper iodide clusters with Cu_4_I_4_ cores, systematically investigating their XEL properties and illustrating that the copper clusters have the potential to be used as scintillators. However, research in this area did not attract significant attention in the following years. In 2019, Su and coworkers reported a multiresponsive copper iodide cluster exhibiting UV light-induced luminescence, near-infrared two-photon absorption (NIR-TPA)-induced luminescence, and X/γ-ray-excited luminescence.^8^ Unfortunately, the strength of the X/γ-ray excited luminescence remained limited, and no direct comparison was made with commercially available scintillators, which hindered broader interest in the field. A turning point emerged at the end of 2022, when Huang's, Zhao's, and Liu's teams independently reported a series of copper iodide cluster scintillators.^9,10^ These copper iodide cluster scintillators exhibit efficient and long-term stable radioluminescence. Meanwhile, high-resolution X-ray imaging of actual samples was successfully achieved using the copper iodide cluster-based scintillator screens, underscoring their promising practical applications. Liu and Qiu highlighted these advances in 2023, emphasizing that copper iodide clusters hold great potential as scintillators for real-time X-ray imaging.^6^ After that, a series of copper iodide cluster scintillators were successively developed, with rapidly improving radioluminescence performance.^11–16^ Li's group reported the first copper iodide cluster-based MOF scintillator in 2023. The formation of a crystalline framework endowed the material with high XEL efficiency and excellent chemical stability. Moreover, by incorporating polyvinylpyrrolidone (PVP) during the *in situ* synthesis process, regular rod-like microcrystals were obtained, further enhancing both the XEL intensity and processability of the scintillator.^17^

In addition to copper iodide cluster scintillators, two copper-based alloy clusters were reported in 2023 by Li's group and Bakr's group, respectively. The first atomically precise gold–copper cluster scintillator with thermally activated delayed fluorescence (TADF) properties was reported.^18^ The TADF characteristic enables the gold–copper cluster to achieve an ultrahigh exciton utilization efficiency. Combined with efficient X-ray absorption arising from the heavy-atom effect and suppressed nonradiative transitions due to close packing in the crystalline state, the gold–copper cluster demonstrates outstanding radioluminescence performance. Subsequently, Bakr and co-workers reported an aggregation-induced emission (AIE)-active gold–copper cluster. The AIE characteristic imparts intense radioluminescence to the gold–copper cluster, with a relative light yield (LY) estimated at 13 600–17 000 photons per MeV.^19^ Subsequently, an AIE-active Cu_4_@Cu_3_ cluster without halogen or other metal atoms was reported by Yao's team, indicating that the pure copper clusters also have the potential to become scintillators.^20^

Recently, copper cluster scintillators with novel radioluminescence properties have received increased attention. For instance, the incorporation of a 9-(pyridin-4-yl)-9*H*-carbazole (Cz-Py) ligand into a Cu_2_I_2_ core has endowed the resulting material with X-ray excited afterglow emission.^21^ In another study, an open-shell luminescent radical ligand was employed to construct a copper cluster, yielding a radical-based copper cluster scintillator that achieves 100% exciton utilization efficiency along with a short radiative decay lifetime on the nanosecond scale.^22^ Furthermore, temperature-independent radioluminescence has been observed in a TADF-active copper cluster scintillator, demonstrating its potential for high-resolution X-ray imaging under variable thermal conditions.^23^

## 3. Scintillation mechanism of copper clusters

**Fig. 1:**
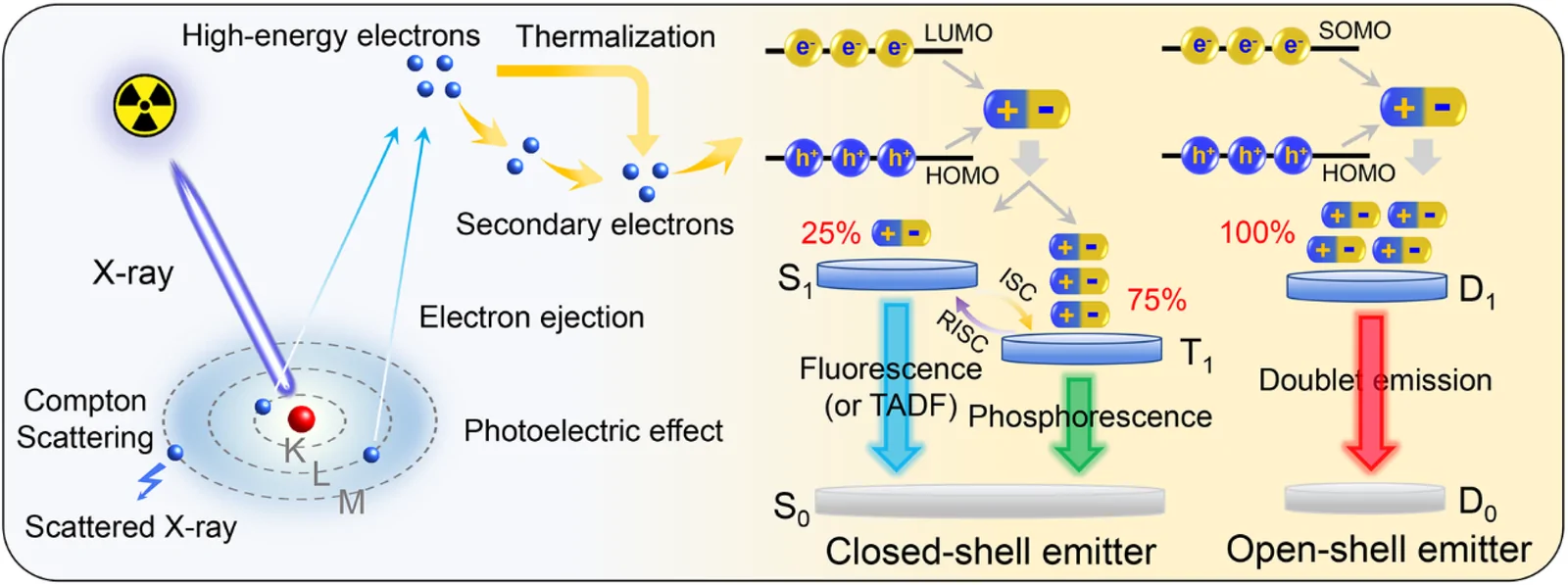
Radioluminescence mechanism of copper cluster scintillators.^28^ Reproduced from ref. 28 with permission from American Chemical Society, copyright 2023.

As shown in Fig. 1,^28^ the radioluminescence mechanism of copper cluster scintillators generally involves four sequential stages. In the first stage, high-energy X-ray photons are absorbed by atoms within the copper cluster through the photoelectric effect and Compton scattering, generating primary high-energy electrons. Due to their higher atomic number, heavy atoms exhibit a greater capacity for X-ray absorption, leading to more efficient ejection of outer-shell electrons. In the second stage, these primary electrons rapidly produce numerous secondary electrons and holes *via* inelastic electron scattering and the Auger effect within an ultrashort timescale (on the order of femtoseconds to picoseconds). During the third stage, the generated charge carriers undergo thermal relaxation and subsequently localize into the lowest unoccupied molecular orbital (LUMO) and highest occupied molecular orbital (HOMO) of the constituent molecules and then interact to form excitons. In the final stage, exciton recombination gives rise to radiative emission through various pathways, yielding singlet and triplet excitons (in closed-shell systems) or doublet excitons (in open-shell radical systems), depending on the electronic structure of the material. While the initial three stages are largely consistent across different copper cluster scintillators, the fourth stage (governing the nature of radiative transitions) varies significantly and encompasses diverse luminescence mechanisms such as phosphorescence, TADF, and doublet emission. Accordingly, this section is organized into four parts based on the distinct radiative transition processes.

### 3.1 Phosphorescent copper cluster scintillators

**Fig. 2:**
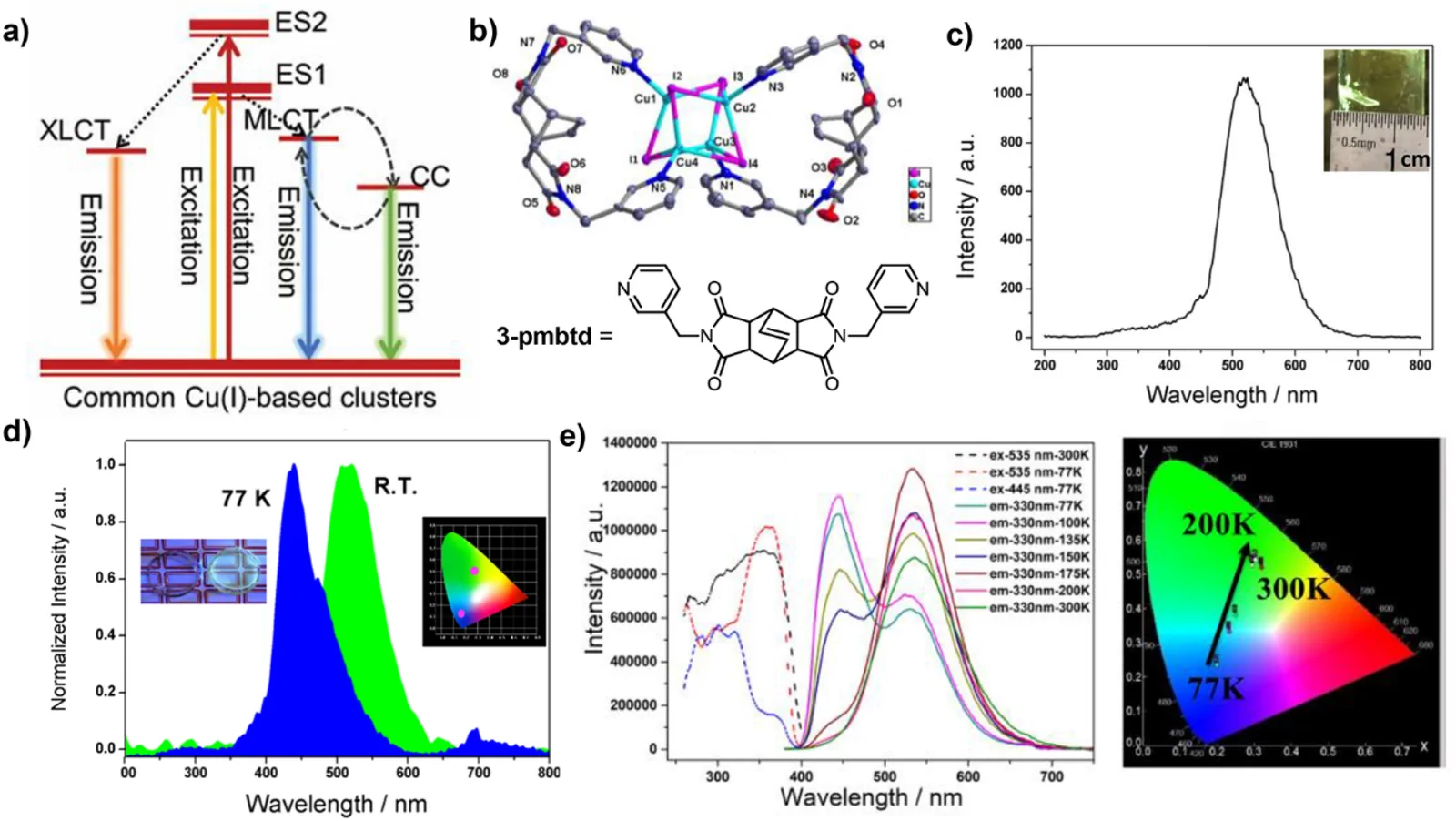
(a) Energy diagrams of copper clusters.^53^ Reproduced from ref. 53 with permission from Wiley, copyright 2022. (b) Structure of Cu_4_I_4_(3-pmbtd)_2_. (c) XEL spectrum of Cu_4_I_4_(3-pmbtd)_2_ at room temperature. (d) XEL spectra of Cu_4_I_4_(3-pmbtd)_2_@PMMA at 77 K and room temperature, respectively. Inset: The photographs and CIE coordinates of the sample. (e) Temperature-dependent luminescence spectra and corresponding CIE coordinates of Cu_4_I_4_(3-pmbtd)_2_@PMMA.^8^ Reproduced from ref. 8 with permission from American Chemical Society, copyright 2019.

Phosphorescence emission represents one of the most significant radiative transition pathways in copper clusters. The triplet excited states of these clusters are typically classified into two types: one is the metal-to-ligand charge transfer state (MLCT) and the other is the cluster-centered excited state (CC).^24^ For copper halide clusters, the halide-to-ligand charge transfer state (XLCT) represents another important type of triplet excited state, which typically coexists with the MLCT state.^25^ These excited states have different energy levels, related to different emission wavelengths (Fig. 2a). Different emission channels are typically activated at distinct temperature ranges. Consequently, copper clusters often exhibit multiple emission colors under varying temperature conditions. For example, Su and coworkers reported a multi-responsive copper cluster based on a Cu_4_I_4_ core coordinated by two 3-pmbtd (3-pmbtd = 2,6-bis(pyridin-3-ylmethyl)-3*a*,4,4*a*,7*a*,8,8*a*-hexahydro-4,8-ethenopyrrolo[3,4-*f*]isoindole-1,3,5,7(2*H*,6*H*)-tetraone) ligands (Cu_4_I_4_(3-pmbtd)_2_), as shown in Fig. 2b.^8^Cu_4_I_4_(3-pmbtd)_2_ exhibits bright green radioluminescence at room temperature (Fig. 2c). In order to further detect and compare low temperature radioluminescence, a Cu_4_I_4_(3-pmbtd)_2_-based monolith gel sample (Cu_4_I_4_(3-pmbtd)_2_@PMMA) was prepared (PMMA = polymethyl methacrylate), which exhibits blue radioluminescence at 77 K and green radioluminescence at room temperature (Fig. 2d). These radioluminescence behaviors are consistent with the photoluminescence behaviors of Cu_4_I_4_(3-pmbtd)_2_. As shown in Fig. 2e, upon excitation with 330 nm UV light at room temperature, Cu_4_I_4_(3-pmbtd)_2_ exhibits a broad emission band centered at 535 nm, corresponding to intense green luminescence. This green emission has a high photoluminescence quantum yield (PLQY) of 58% and a long decay lifetime of 13.1 µs, which are consistent with phosphorescence originating from the CC triplet excited states. Upon cooling from 300 K to 175 K, the intensity of the green emission steadily increases due to suppression of non-radiative thermal deactivation pathways. Further cooling from 175 K to 77 K leads to a gradual decrease in the green emission, concurrently accompanied by the emergence and progressive enhancement of a blue emission centered at 445 nm, which can be assigned to the XLCT triplet states.

### 3.2 TADF copper cluster scintillators

**Fig. 3:**
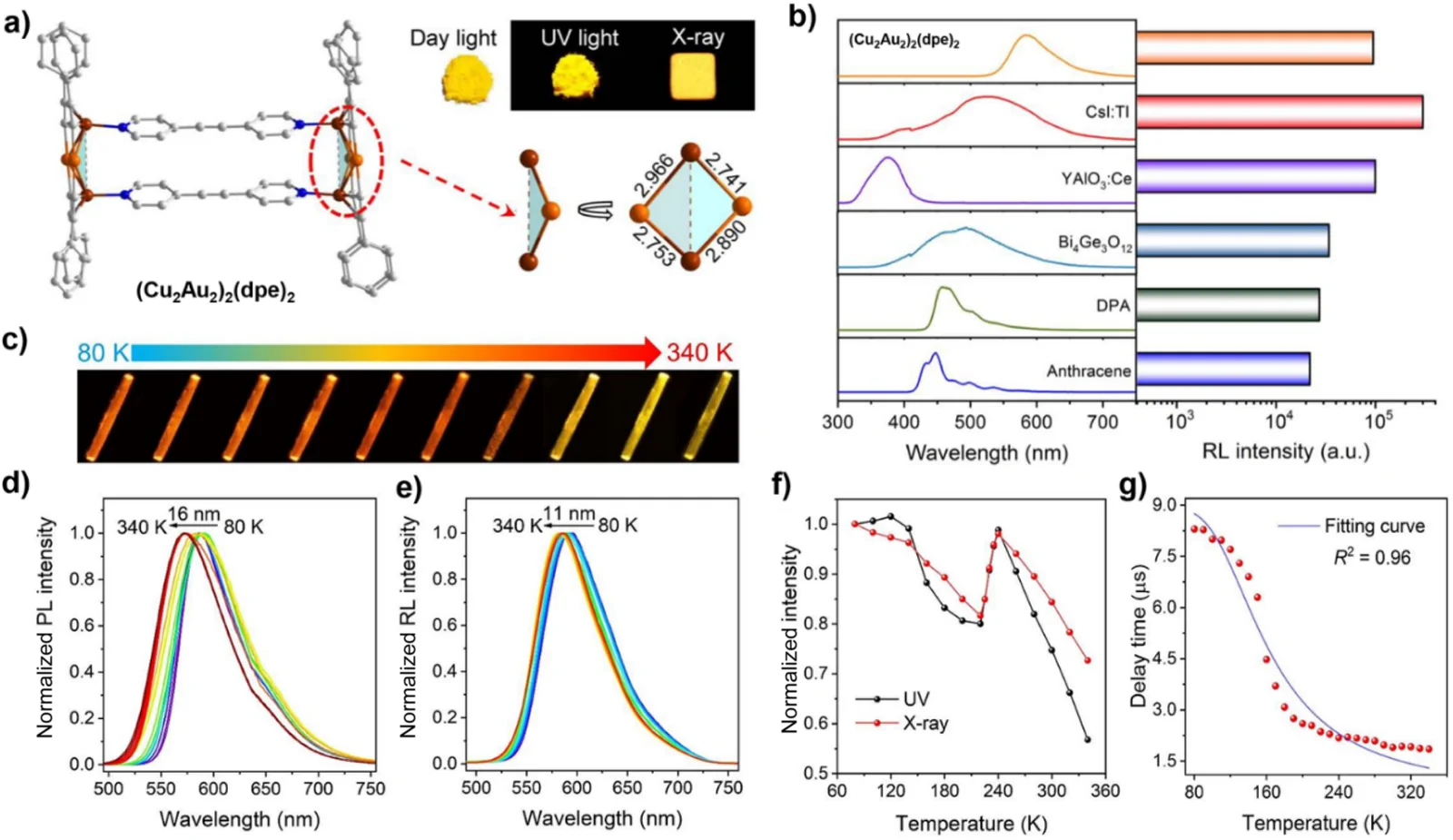
(a) Crystal structure and photographs of (Cu_2_Au_2_)_2_(dpe)_2_ (color codes for atoms: orange, Au; brown, Cu; blue, N; and gray, C). (b) Comparison of the XEL spectra of (Cu_2_Au_2_)_2_(dpe)_2_ and commercial scintillators. (c) Photographic image of the temperature-variable luminescence of a crystal of (Cu_2_Au_2_)_2_(dpe)_2_ under UV light excitation. (d and e) Normalized temperature-dependent photoluminescence spectra and XEL spectra of (Cu_2_Au_2_)_2_(dpe)_2_. (f) Normalized luminescence intensity of (Cu_2_Au_2_)_2_(dpe)_2_ under UV light and X-ray excitation as a function of temperature. (g) Temperature-dependent luminescence lifetimes of (Cu_2_Au_2_)_2_(dpe)_2_. The blue solid line is the fitting curve of the modified Boltzmann equation.^18^ Reproduced from ref. 18 with permission from American Chemical Society, copyright 2023.

In 2023, Li and coworkers reported an atomically precise gold–copper cluster scintillator, (Cu_2_Au_2_)_2_(dpe)_2_ (dpe = 1,2-di(pyridin-4-yl)ethyne), exhibiting TADF properties (Fig. 3a).^18^ The cluster can be readily synthesized *via* the solvent evaporation method and exhibits high stability toward water and oxygen. The TADF properties endow the cluster with an ultrahigh exciton utilization efficiency and favorable XEL performance (Fig. 3b). The TADF characteristic of a molecule can be confirmed through temperature-dependent emission spectra and luminescence lifetime measurements. Thus, the temperature-dependent emission spectra of (Cu_2_Au_2_)_2_(dpe)_2_ under UV light and X-ray excitations in the temperature range from 80 K to 340 K were measured. As shown in Fig. 3c–e, the emission wavelength of the cluster exhibited a continuous blue shift, attributed to thermally activated reverse intersystem crossing (RISC) from the lowest triplet excited state (T_1_) to the lowest singlet excited state (S_1_). As the temperature increased, the contribution of TADF (short-wavelength emission) increased, while that of phosphorescence (long-wavelength emission) decreased. Moreover, a significant emission enhancement in the temperature range from 220 K to 240 K was observed (Fig. 3f), which is consistent with the behavior of typical TADF molecules. Next, the temperature-dependent luminescence lifetime decay curve of (Cu_2_Au_2_)_2_(dpe)_2_ from low to high temperature was recorded. As shown in Fig. 3g, at low temperatures, (Cu_2_Au_2_)_2_(dpe)_2_ exhibits relatively long luminescence lifetime, primarily attributed to radiative transitions from the triplet excited state to the ground state. Upon continuous heating, the thermally activated RISC process is initiated, resulting in a gradual decrease in the luminescence lifetime as the TADF process becomes active. The lifetime variation of (Cu_2_Au_2_)_2_(dpe)_2_ can be described by the modified Boltzmann equation:
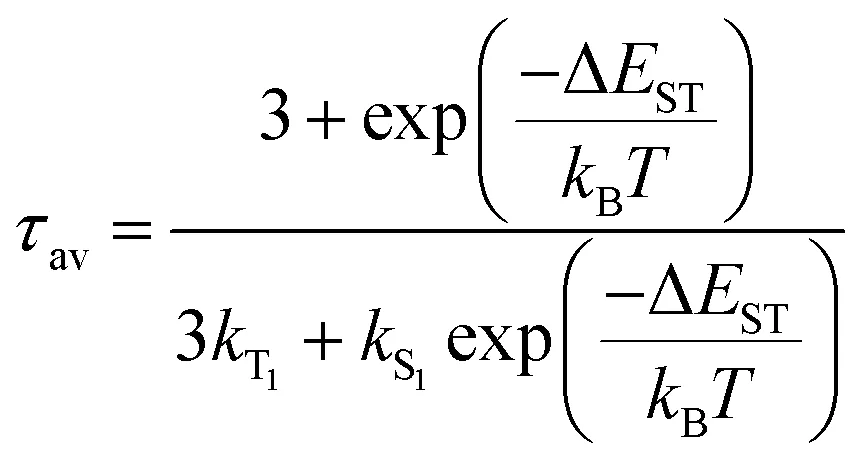
where *τ*_av_, *k*_B_ and *T* are the experimental average lifetime, Boltzmann constant and temperature, respectively. *k*_T_1__ is the phosphorescence radiative transition rate, *k*_S_1__ is the instantaneous fluorescence radiative transition rate, Δ*E*_ST_ is the energy gap between S_1_ and T_1_, which can be determined by fitting the temperature-dependent lifetime decay curve (Fig. 3g). As a result, *k*_T_1__ was calculated as 1.1 × 10^5^ s^−1^ and *k*_S_1__ was calculated as 1.3 × 10^7^ s^−1^. The *k*_PL_ value (1.6 × 10^5^ s^−1^) is very close to *k*_T_1__, indicating that the radiative transition of the cluster comprises both phosphorescence and TADF contributions. Furthermore, Δ*E*_ST_ is calculated to be 0.05 eV, which is significantly lower than the typical threshold of 0.37 eV required for efficient TADF emitters.^26^

**Fig. 4:**
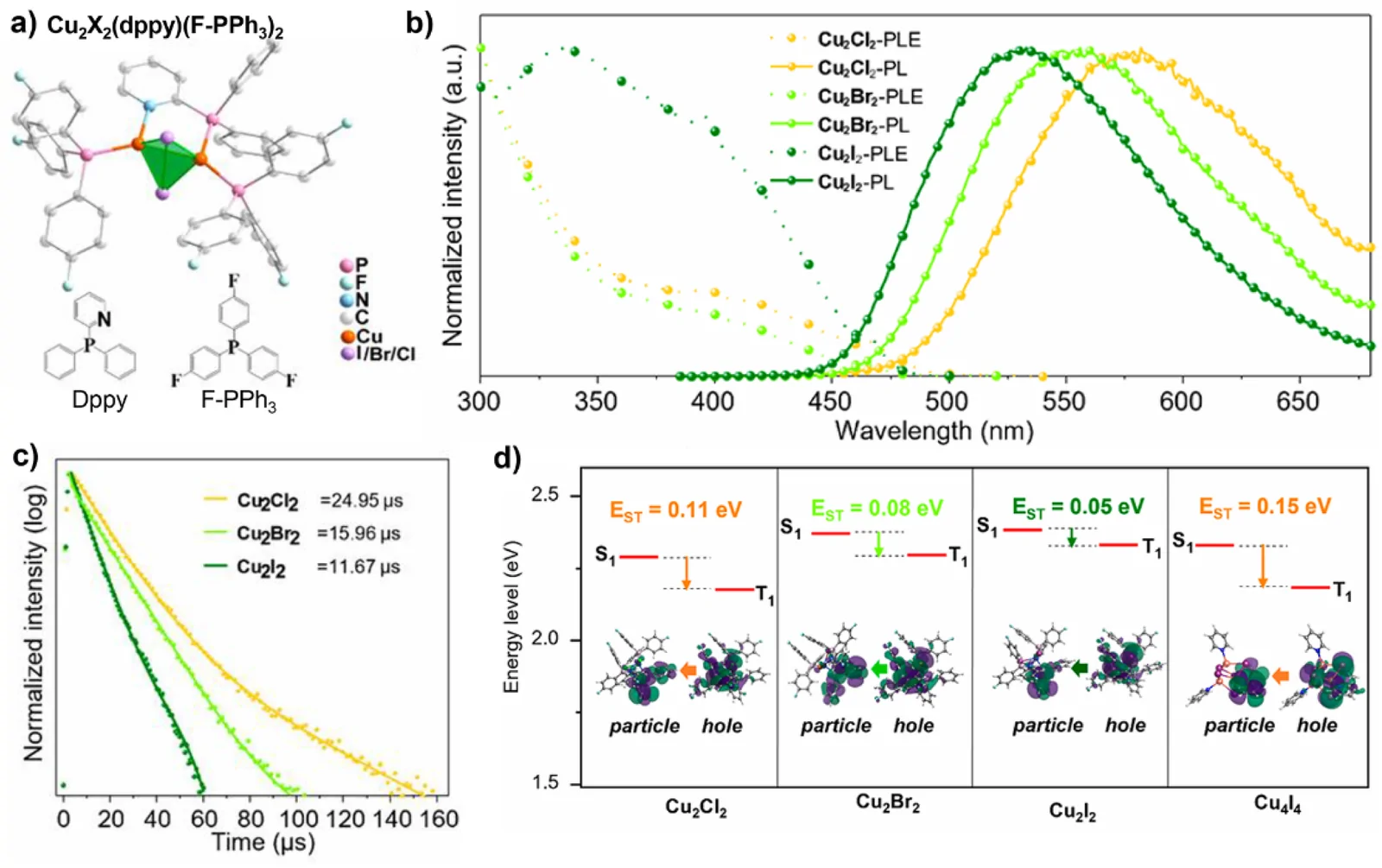
(a) Molecular structure of Cu_2_X_2_(dppy)(F-PPh_3_)_2_. (b) Normalized emission and excitation spectra of Cu_2_X_2_(dppy)(F-PPh_3_)_2_. (c) Excited-state lifetimes of Cu_2_X_2_(dppy)(F-PPh_3_)_2_ upon excitation at 365 nm. (d) Calculated Δ*E*_ST_ for Cu_2_X_2_(dppy)(F-PPh_3_)_2_. Insets show the charge densities for the holes and particles from natural orbital transitions from S_0_ to S_1_.^12^ Reproduced from ref. 12 with permission from American Chemical Society, copyright 2023.

The halogen atoms have important influence on the TADF properties of copper halide clusters. Omar and coworkers reported a series of TADF copper halide clusters (Cu_2_X_2_(dppy)(F-PPh_3_)_2_, dppy = diphenyl-2-pyridylphosphine, F-PPh_3_ = tri(4-fluorophenyl)phosphine, X = halogen) with different radioluminescence colors and excited-state lifetimes (Fig. 4a).^12^ As shown in Fig. 4b, the steady-state photoluminescence spectra of these clusters exhibit broad emission bands in the visible region, centered at 533 nm (iodine), 553 nm (bromine), and 580 nm (chlorine), respectively. Meanwhile, a distinct trend in the emission maxima is observed, correlating with the nature of the halides, as evidenced by a progressive blue shift from Cu_2_Cl_2_(dppy)(F-PPh_3_)_2_ to Cu_2_I_2_(dppy)(F-PPh_3_)_2_. As shown in Fig. 4c, the photoluminescence decay times at room temperature gradually decrease from 24.95 µs for Cu_2_Cl_2_(dppy)(F-PPh_3_)_2_ to 11.67 µs for Cu_2_I_2_(dppy)(F-PPh_3_)_2_. The different halogen atoms endow the clusters with different Δ*E*_ST_, which is 0.11 eV (chlorine), 0.08 eV (bromine) and 0.05 eV (iodine), respectively (Fig. 4d). Natural transition orbitals (NTOs) analysis indicates that the optical absorption from S_0_ to S_1_ can be attributed to electronic transitions from the “hole” state, which is delocalized across the Cu_2_X_2_ core, to the “particle” state, predominantly localized on the bridging organic ligand of dppy. This result indicates that the optical transition is predominantly characterized by a (M + X)LCT character between the Cu_2_X_2_ core and dppy ligands, with negligible contribution from F-PPh_3_. Structurally, the Cu–Cu interaction in Cu_2_I_2_(dppy)(F-PPh_3_)_2_ is stronger than the intermolecular interactions observed in Cu_2_Cl_2_(dppy)(F-PPh_3_)_2_ and Cu_2_Br_2_(dppy)(F-PPh_3_)_2_, thereby increasing the possibility of orbital overlap. The favorable balance between luminescence and orbital overlap of Cu_2_I_2_(dppy)(F-PPh_3_)_2_ renders it the highest radioluminescence performance among the three clusters.

**Fig. 5:**
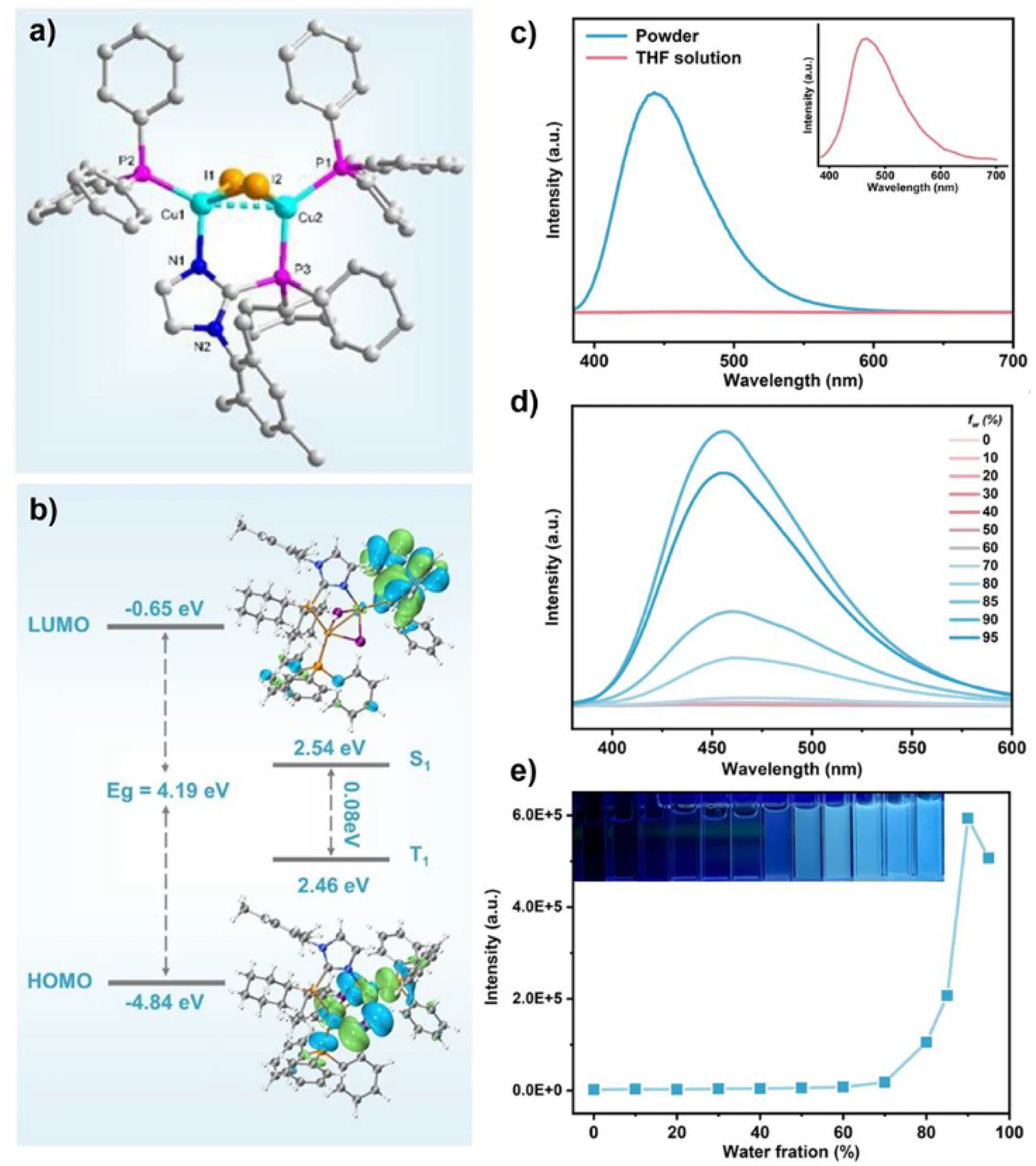
(a) Molecular structure of Cu_2_I_2_L(PPh_3_)_2_. (b) HOMO and LUMO distribution, frontier orbital energies, and S_1_ and T_1_ energy levels of Cu_2_I_2_L(PPh_3_)_2_. (c) Emission spectra of Cu_2_I_2_L(PPh_3_)_2_ in the powder state and THF solution measured under identical conditions. Inset: Enlarged emission spectrum of Cu_2_I_2_L(PPh_3_)_2_ in THF solution. (d) Emission spectra of Cu_2_I_2_L(PPh_3_)_2_ in mixed H_2_O/THF solutions with different water fractions. (e) The photoluminescence intensity of Cu_2_I_2_L(PPh_3_)_2_ as a function of water fraction. Inset: Photographs of Cu_2_I_2_L(PPh_3_)_2_ in mixed H_2_O/THF solutions with different water fractions under 365 nm UV light.^27^ Reproduced from ref. 27 with permission from Wiley, copyright 2025.

Hong and Chen's research team reported a TADF/AIE-active copper halide cluster of Cu_2_I_2_L(PPh_3_)_2_ (L = 1-(2,4,6-trimethylphenyl)-2-(dicyclohexylphosphino)imidazole), which demonstrates excellent water oxygen stability and photochemical stability (Fig. 5a). Frontier orbital composition analysis reveals that the HOMO of Cu_2_I_2_L(PPh_3_)_2_ is primarily localized on the Cu_2_I_2_ core, whereas the LUMO is predominantly distributed over the triphenylphosphine ligand (Fig. 5b). Orbital transition analysis indicates that the lowest lying transitions of Cu_2_I_2_L(PPh_3_)_2_ possess a significant (M + X)LCT character. The Δ*E*_ST_ value of Cu_2_I_2_L(PPh_3_)_2_ calculated by the time-dependent density functional theory (TD-DFT) method is 0.08 eV. Cu_2_I_2_L(PPh_3_)_2_ exhibits intense emission in the solid state but negligible emission in dissolved state, consistent with typical AIE characteristics (Fig. 5c). Integrating the AIE effect into TADF copper halide clusters enables efficient luminescence in aggregated states and enhances the utilization of triplet excitons, thereby offering promising opportunities for the development of high-performance scintillator materials.^27^

### 3.3 Doublet emission copper cluster scintillators

**Fig. 6:**
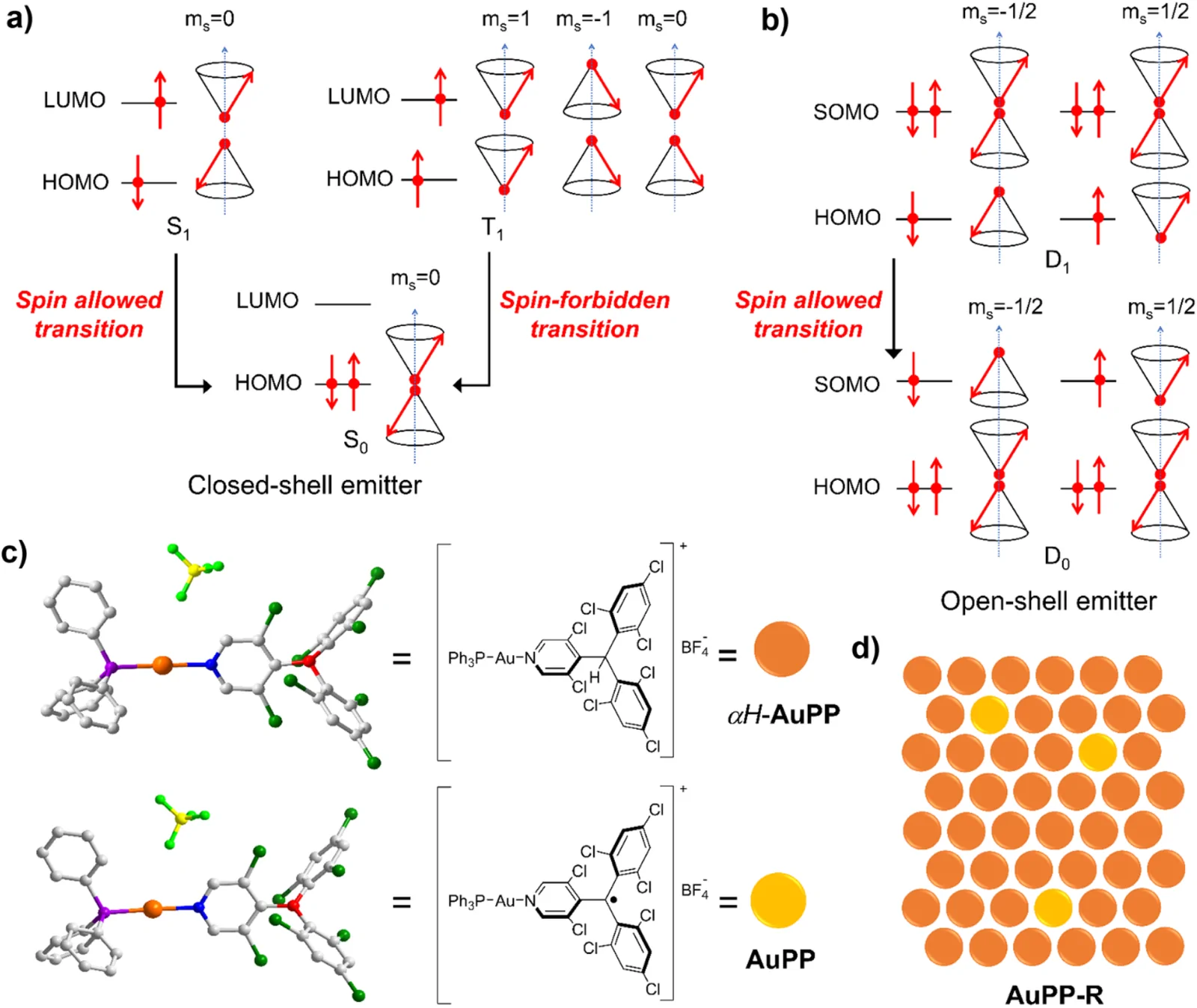
Schematic diagrams of the excited-state spin configurations of (a) a closed-shell emitter and (b) an open-shell emitter. *m*_s_ is the projection value of the total spin quantum number along the direction of the external magnetic field. (c) Crystal structures of AuPP and αH-AuPP. (d) Composition diagram of AuPP-R.^28^ Reproduced from ref. 28 with permission from American Chemical Society, copyright 2023.

Stable luminescent radicals are open-shell emitters with unique doublet emission characteristics. In 2023, Li's group reported the first stable luminescent radical-based X-ray scintillator, AuPP-R, which exhibited a high XEL efficiency as well as excellent stability. Unlike closed-shell emitters, whose emission is typically limited by the spin-forbidden transition from T_1_ to S_0_ (Fig. 6a), open-shell emitters exhibit an intrinsically high exciton utilization efficiency. As shown in Fig. 6b, in open-shell emitters, excitons occupy the excited doublet state (D_1_) and subsequently decay radiatively to the ground doublet state (D_0_), enabling a theoretical maximum exciton utilization efficiency of 100%, indicating that doublet emission is highly favourable for radioluminescence applications.^28^ This strategy was subsequently applied to the construction of organic light-emitting organic scintillators.^29^

**Fig. 7:**
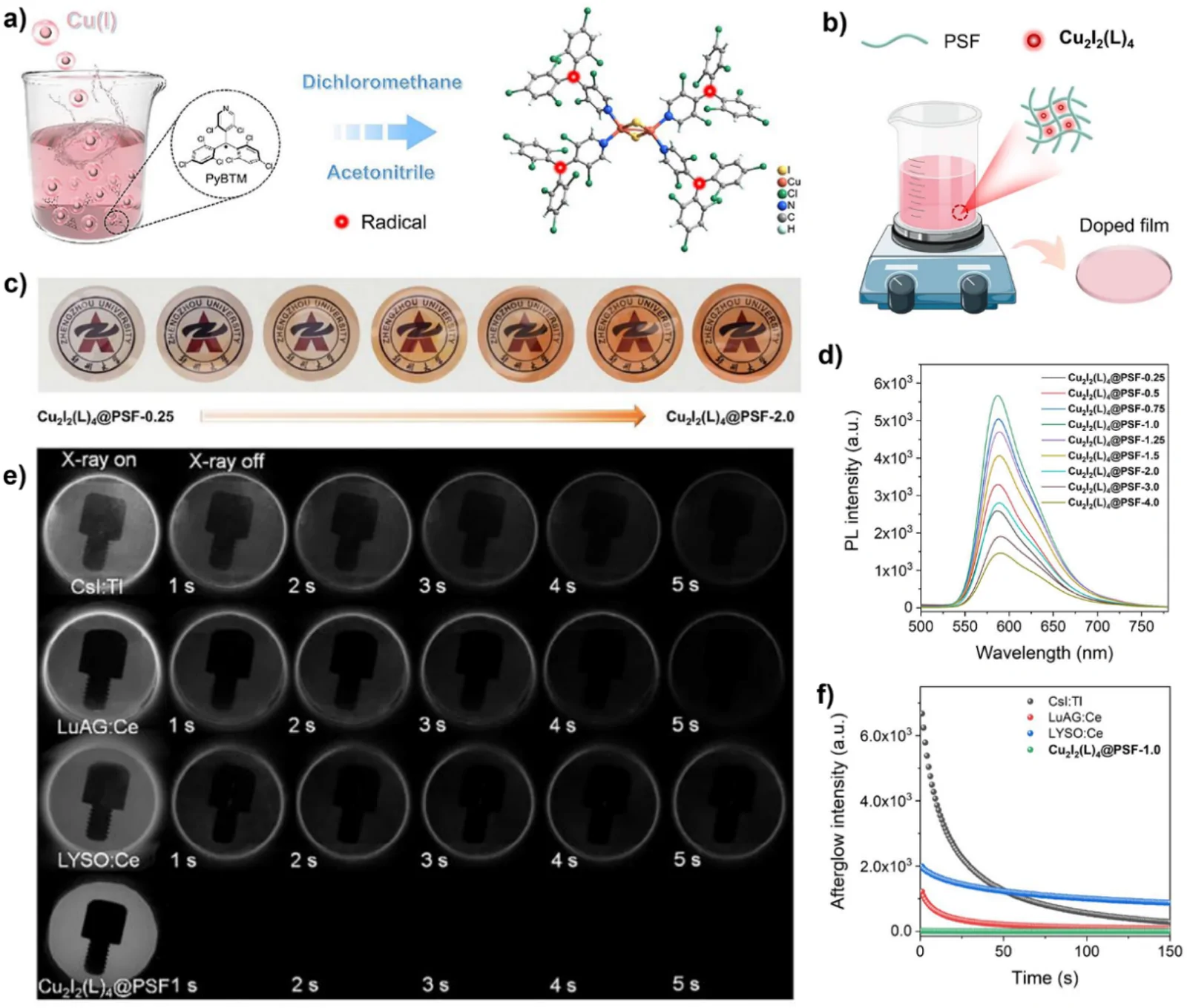
(a) Schematic representation of the solution synthesis process of Cu_2_I_2_(L)_4_ and the crystal structure of Cu_2_I_2_(L)_4_. (b) Schematic diagram of the preparation of the doped film using an *in situ* strategy. (c) Cu_2_I_2_(L)_4_@PSF-*R* (*R* is the doping ratio (wt%) of Cu_2_I_2_(L)_4_ to PSF). (d) Luminescence spectra of Cu_2_I_2_(L)_4_@PSF-*R* (*λ*_ex_ = 400 nm). (e) Continuous imaging of a screw after X-ray irradiation using commercial scintillator wafers and Cu_2_I_2_(L)_4_@PSF-1.0. All the samples were irradiated with X-rays for 30 s before testing. (f) Afterglow decay curves of commercial scintillator wafers and Cu_2_I_2_(L)_4_@PSF-1.0 after X-ray irradiation.^22^ Reproduced from ref. 22 with permission from Wiley, copyright 2025.

Based on this work, they further synthesized an open-shell copper halide cluster, Cu_2_I_2_(L)_4_, using the luminescent radical ligand (L = (3,5-dichloro-4-pyridyl)bis(2,4,6-trichlorophenyl)methyl) (Fig. 7a).^22^ Meanwhile, an *in situ* fabrication strategy was employed to embed Cu_2_I_2_(L)_4_ into polysulfone (PSF), yielding a scintillator screen designated as Cu_2_I_2_(L)_4_@PSF-1.0 (Fig. 7b–d). Cu_2_I_2_(L)_4_@PSF-1.0 exhibits intense XEL, excellent X-ray photostability, and high thermal and water–oxygen stability, enabling high-resolution imaging of the internal structures of various objects. The spin-allowed doublet emission of Cu_2_I_2_(L)_4_ enables a theoretical 100% exciton utilization efficiency and is associated with a short radiative decay lifetime on the nanosecond scale, making Cu_2_I_2_(L)_4_@PSF-1.0 suitable for continuous X-ray imaging without residual images (Fig. 7e and f).

### 3.4 Copper cluster scintillators with other luminescence processes

**Fig. 8:**
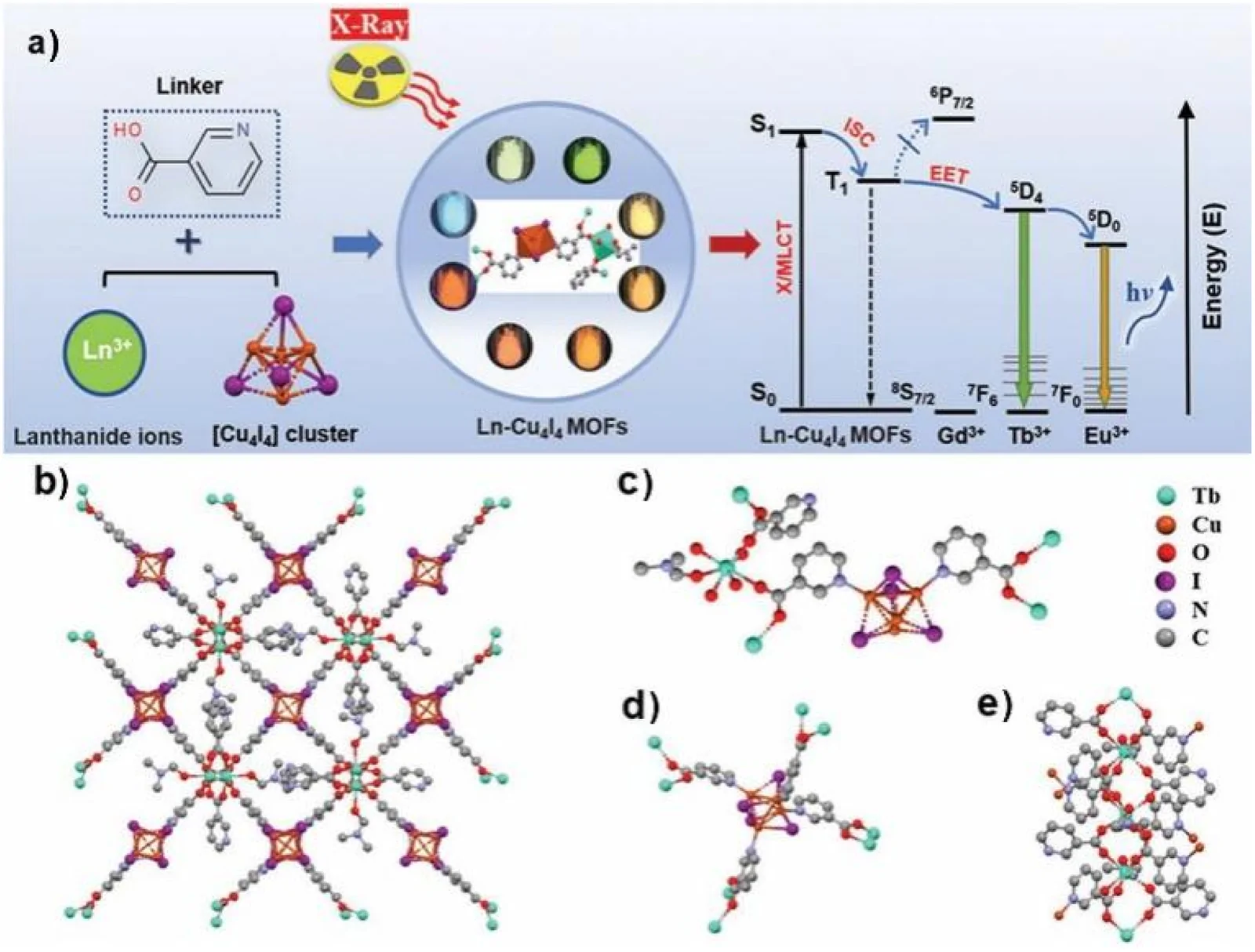
(a) A schematic representation of the rational design and the relative XEL processes of Ln–Cu_4_I_4_ MOFs. (b) Perspective view of the three-dimensional crystal structure of Tb–Cu_4_I_4_ MOFs, (c) asymmetric unit of Tb–Cu_4_I_4_ MOFs, (d) connection mode of the cubic Cu_4_I_4_ center, and (e) connection mode of Ln–O centers.^10^ Reproduced from ref. 10 with permission from Wiley, copyright 2022.

Zhao's group reported a series of lanthanide(iii)–Cu_4_I_4_ heterometallic organic framework (Ln–Cu_4_I_4_ MOF)-based X-ray scintillators, developed by rationally assembling X-ray absorption centers (Cu_4_I_4_ clusters) and luminescent chromophores (Ln(iii) ions) in a well-defined structural configuration (Fig. 8a).^10^ Under X-ray irradiation, the heavy inorganic units of Cu_4_I_4_ clusters absorb X-ray energy and generate triplet excitons through XLCT synergized with MLCT, forming an X/MLCT excited state. This excited state subsequently sensitizes Tb(iii) ions, enabling intense XEL *via* efficient excitation energy transfer. Among the Ln–Cu_4_I_4_ MOFs, Tb–Cu_4_I_4_ MOF scintillators exhibit high humidity and radiation resistance, an excellent linear response to X-ray dose rate, and a high LY of 29 379 ± 3000 photons per MeV. Notably, the LY of Tb–Cu_4_I_4_ MOFs is about three times higher than that of the control Tb(iii) complex.

## 4. Design strategy of copper cluster scintillators

Copper clusters usually exhibit intense photoluminescence through phosphorescence and TADF processes. However, the atomic number of copper is only 29, and its limited X-ray absorption capacity restricts the application of copper clusters as scintillators. Thus, to improve the X-ray absorption capacity, heavy atoms are usually introduced to the copper clusters to form copper alloy clusters or copper halide clusters. To improve the stability, copper cluster-based assembly materials were further developed. According to their structural characteristics, copper cluster-based scintillators are classified into three categories in this review: copper clusters/copper alloy clusters, copper halide clusters and copper cluster-based assembly materials. In this part, the design strategy for copper cluster scintillators will be introduced as three parts according to their different structural features.

### 4.1 Copper clusters and copper alloy clusters

**Fig. 9:**
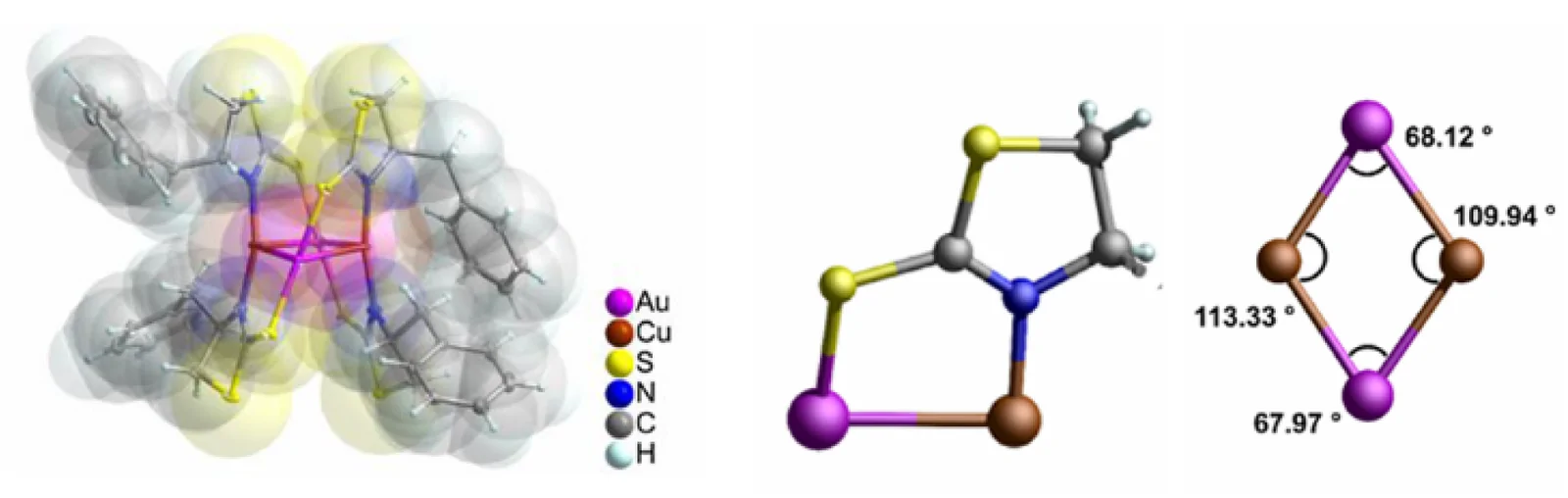
Crystal structure of Cu_2_Au_2_(*R*-BTT)_4_.^19^ Reproduced from ref. 19 with permission from American Chemical Society, copyright 2023.

In 2023, Li's group reported the first atomically precise gold–copper alloy cluster-based scintillator, (Cu_2_Au_2_)_2_dpe_2_, featuring two Cu_2_Au_2_ cores bridged by two dpe ligands.^18^ The compound was synthesized *in situ via* solvent volatilization at room temperature. As illustrated in Fig. 3a, single-crystal X-ray diffraction analysis reveals that each inner core adopts a spatially defined parallelogram geometry, while the periphery is stabilized by the cooperative coordination of four phenylacetylene ligands and two dpe ligands, resulting in a splint-shaped molecular architecture. The observed Au–Cu bond lengths range from 2.703 to 2.985 Å, which are shorter than the sum of the corresponding van der Waals radii (1.40 + 1.66 Å = 3.06 Å), indicating significant Au–Cu interaction. Four alkynyl groups exhibit a µ_2_-η^1^,η^2^ coordination mode, wherein all alkynyl moieties are σ-bonded to Au atoms and simultaneously π-interact with copper atoms. In the same year, another Au–Cu alloy cluster scintillator of Cu_2_Au_2_(*R*-BTT)_4_ was reported by Omar's team.^19^Cu_2_Au_2_(*R*-BTT)_4_ was synthesized *via* a one-step solution method with [Cu(CH_3_CN)_4_]BF_4_, chloro(dimethylsulfide)gold(i) and (*R*)-4-benzylthiazolidine-2-thione (*R*-BTT). Single-crystal X-ray diffraction analysis revealed that Cu_2_Au_2_(*R*-BTT)_4_ crystallizes in the chiral space group *P*2_1_ (no. 4). As illustrated in Fig. 9, each Cu_2_Au_2_(*R*-BTT)_4_ contains two gold atoms, two copper atoms, and four *R*-BTT ligands, forming a quasi-rhombic planar tetranuclear alloy structure bridged by *R*-BTT ligands. The average Au–Cu distance is 2.851 Å, significantly shorter than the sum of the van der Waals radii of gold and copper (3.06 Å), indicating the presence of metallophilic interactions that contribute to structural stabilization. The *R*-BTT ligands are alternately oriented above and below the quasi-rhombic plane and adopt a uniform coordination mode, wherein the nitrogen atom coordinates to the copper atom while the thioketone sulfur atom binds exclusively to gold atoms, resulting in a rigid five-membered ring (Au–Cu–N–C–S). This bidentate binding motif enables the *R*-BTT ligands to effectively stabilize the planar tetranuclear framework through tight chelation.

**Fig. 10:**
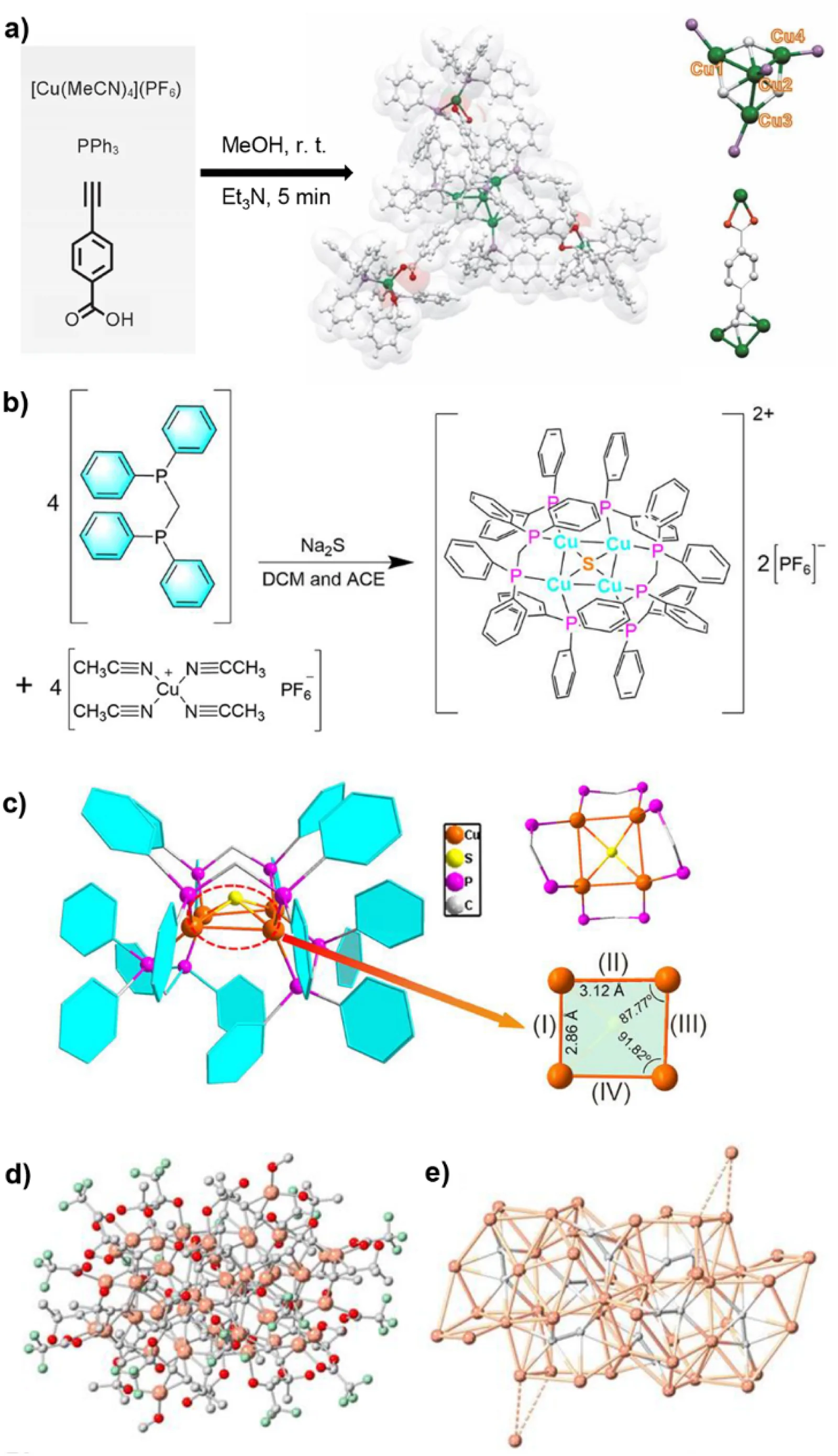
(a) General synthetic route for Cu_4_@Cu_3_.^20^ Reproduced from ref. 20 with permission from Springer Nature, copyright 2024. (b) General synthetic route for Cu_4_S(dppm)_4_(PF_6_)_2_. (c) Crystal structure and coordination mode of Cu_4_S(dppm)_4_(PF_6_)_2_.^23^ Reproduced from ref. 23 with permission from Wiley, copyright 2025. (d) The total structure of (C_2_)_8_@Cu_50_. (e) Eight C_2_^2−^ ions embedded in the metal framework of Cu_50_.^30^ Reproduced from ref. 30 with permission from Wiley, copyright 2025.

In 2024, an AIE copper cluster scintillator of Cu_4_@Cu_3_ was reported by Yao's team.^20^Cu_4_@Cu_3_ crystallizes in the triclinic space group *P*1̄ and consists of seven copper atoms, seven PPh_3_, three 4-ethynylbenzoic acid (PE), and one methanol. The central core comprises four copper atoms and three PE ligands arranged into a triangular pyramidal structure featuring an open square geometry (Fig. 10a), where Cu1 and the acetylene groups are bridged in a µ_3_ fashion. The distances of Cu1–Cu2, Cu2–Cu3, and Cu2–Cu4 at 2.453, 2.451, and 2.467 Å are significantly shorter than those observed in the Cu_3_C_3_ ring (3.81–3.83 Å), indicating the electron-deficient character of the three-center, two-electron Cu–C–Cu bonding motif. In contrast, the copper atoms within the Cu_3_C_3_ ring exhibit relatively lower electron deficiency due to their participation in µ_3_-bridging coordination with the acetylene ligands and also engaging in side coordination with the acetylene group, which may contribute to copper's p electrons. The C–Cu2–C bond angle is about 102°, with the carboxyl groups oriented outward, providing favorable coordination sites for the three outer copper centers. Notably, one of the three outer copper ions is coordinated by a methoxide ligand, rendering the entire cluster electrically neutral and consistent with thermodynamic stability requirements.

Recently, Li's group reported a copper–sulfur cluster scintillator, Cu_4_S(dppm)_4_(PF_6_)_2_ (dppm = bis(diphenylphosphino)methane ligand), which has a unique Cu_4_S core.^23^ As illustrated in Fig. 10b, Cu_4_S(dppm)_4_(PF_6_)_2_ was synthesized *via* a one-step reaction involving dppm, [Cu(CH_3_CN)_4_]PF_6_ and Na_2_S in a mixed acetone–dichloromethane solvent system. Cu_4_S(dppm)_4_(PF_6_)_2_ crystallizes in the monoclinic space group *C*2/*c*. The four copper atoms are arranged in a near-planar quadrilateral, with each copper atom bonded to the central sulfur atom, forming a distorted square pyramidal skeleton (Fig. 10c). The base edges of the pyramid are stabilized by four bridging dppm ligands. Each copper center is coordinated to one sulfur atom and two phosphorus atoms, while each dppm ligand chelates two copper atoms in a µ_2_-bridging mode. The four dppm ligands adopt a saddle-shaped conformation, with two folding upward and two folding downward relative to the Cu_4_ plane. The sulfur atom occupies the apical position of the pyramid and protrudes above the tetranuclear copper cluster plane. Within the tetragonal pyramidal framework, only the Cu–Cu bonds along edges (I) and (III) (2.86 Å) are shorter than the sum of the van der Waals radii of copper (2.88 Å), whereas the longer Cu–Cu distances (3.12 Å) along edges (II) and (IV) may arise from steric hindrance induced by the apical sulfur atom. The Cu–Cu–Cu bond angles of 91.82° and 87.77° reflect high structural symmetry. Additionally, adjacent benzene rings of the dppm ligands engage in multiple π⋯π stacking interactions, which enhance the overall structural stability of the cluster.

In addition to low-nuclearity copper clusters, high-nuclearity copper clusters have also been reported as X-ray scintillators. Recently, Sun and coworkers reported an atomically precise high-nuclearity copper alkyne cluster scintillator, (C_2_)_8_@Cu_50_.^30^(C_2_)_8_@Cu_50_ crystallizes in the monoclinic space group *P*2_1_/*n*, with half a cluster located in the asymmetric unit due to the Cu_50_ core residing on a crystallographic inversion center. As shown in Fig. 10d, (C_2_)_8_@Cu_50_ is neutral, and its overall shape resembles a rod-like structure with a length-to-width ratio of approximately 2 : 1 (1.5/0.8 nm), excluding the organic ligand shell. Eight C_2_^2−^ ions are embedded within a highly complex metal framework composed of 50 copper atoms (Fig. 10e). The C_2_^2−^ ions are generated *in situ* from the 2-methyl-3-butyn-2-ol (Hmbo) ligand *via* cleavage of the C_sp_–C_sp^3^_ single bond at room temperature. The electron-withdrawing hydroxyl group is likely responsible for facilitating the low-temperature C_sp_–C_sp^3^_ bond cleavage in Hmbo. Each C_2_^2−^ ion exhibits versatile coordination modes, involving σ, π, and/or mixed σ/π bonding interactions.

The XEL properties of the copper cluster scintillators and copper alloy clusters are summarized in Table 1.

**Table 1:** A summary of the reported copper cluster scintillators

Scintillator	Molecular formula	LY (photons per MeV)	PLQY (%)	*λ*_em_ (nm)	LOD (nGy s^−1^)	Spatial resolution for X-ray imaging (lp per mm)	Ref.
(Cu_2_Au_2_)_2_(dpe)_2_	C_88_H_56_Au_4_Cu_4_N_4_	26 000	31	586	31.7	12.5	18
Cu_2_Au_2_(*R*-BTT)_4_	C_40_H_40_Au_2_Cu_2_N_4_S_8_	17 600	74.4	535	111.7	28.44	19
Cu_4_@Cu_3_	C_208_H_165_Cu_7_O_7_P_10_	—	4	516	—	—	20
Cu_4_S(dppm)_4_(PF_6_)_2_	Cu_4_SP_10_F_12_C_100_H_88_	57 811	92.6	593	71.5	22.1	23
(C_2_)_8_@Cu_50_	C_152_H_172_Cu_50_F_42_O_56_	—	7.35	575	9830	—	30

### 4.2 Copper halide clusters

Due to the heavy atom effect of iodine, copper halide clusters, especially copper iodide clusters, exhibit high X-ray absorption capability.^31^ Coupled with their intrinsic intense luminescence, copper iodide clusters have emerged as the most intensively studied class of copper cluster-based scintillator materials. The structure of the copper halide core plays a critical role in determining the XEL properties.

Among the copper iodide cluster scintillators, those featuring Cu_4_I_4_ cores constitute the majority owing to their stable chemical structures and outstanding luminescent properties. The highly rigid cubic structure of the Cu_4_I_4_ core suppresses John–Teller distortion of excited copper(i) ions and minimizes energy loss caused by structural relaxation.^16^ Meanwhile, the higher content of heavy atoms endows Cu_4_I_4_ clusters with enhanced X-ray absorption capability compared to smaller cores such as Cu_2_I_2_. As previously discussed, early research on copper cluster scintillators primarily focused on Cu_4_I_4_ cluster scintillators.^7,8^

**Fig. 11:**
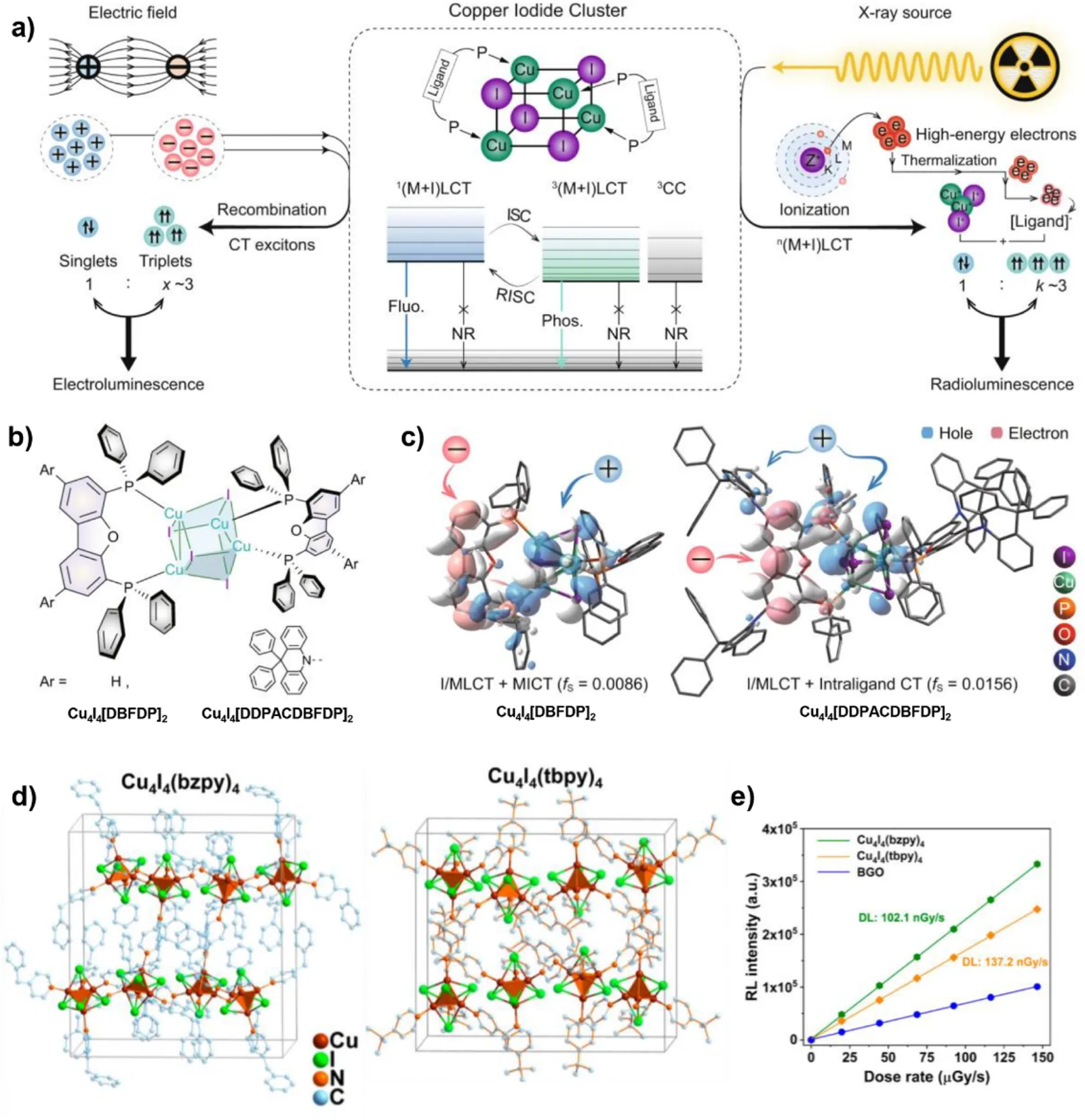
(a) Emission mechanisms of Cu_4_I_4_[DBFDP]_2_ and Cu_4_I_4_[DDPACDBFDP]_2_. (b) Chemical structures of the clusters. (c) Single crystal structures of the clusters and contours of triplet “hole” (blue) and “electron” (pink) for natural transition orbitals. *f*_S_ is the singlet oscillator strength.^16^ Reproduced from ref. 16 with permission from Springer Nature, copyright 2023. (d) Crystal structures of Cu_4_I_4_(bzpy)_4_ and Cu_4_I_4_(*t*bpy)_4_. (e) Detection limits of the cluster microcrystal-based films and BGO crystal wafer.^13^ Reproduced from ref. 13 with permission from American Chemical Society, copyright 2023.

In 2023, Xu's team reported a Cu_4_I_4_ cluster scintillator, Cu_4_I_4_[DDPACDBFDP]_2_ (DDPACDBFDP = 10,10′-(4,6-bis(diphenylphosphanyl)dibenzofuran-2,8-diyl)bis(9,9-diphenylacridine)), which exhibits highly emissive radioluminescence in response to X-ray irradiation through functionalizing biphosphine ligands with acridine (Fig. 11a).^16^ As illustrated in Fig. 11b and c, each ligand of Cu_4_I_4_[DDPACDBFDP]_2_ contains two rigid and strongly electron-donating 9,9-diphenylacridine (DPAC) units. The perpendicular arrangement of the DPAC groups relative to the dibenzofuran moiety prevents conjugation between the lone electron pair on the nitrogen atom of the DPAC unit and the π-system of the dibenzofuran framework. The DPAC donors exert minimal influence on the coordination characteristics of the (dibenzofuran-2,8-diyl)bis(9,9-diphenylacridine) (DBFDP) ligand framework. Single-crystal X-ray diffraction confirmed that Cu_4_I_4_[DDPACDBFDP]_2_ preserved a cubic core with nearly unchanged Cu–I and Cu–Cu bond lengths. In this sense, the DPAC donor enhances ligand-centered charge transfer while exerting minimal influence on X-ray absorption *via* triplet metal-to-iodine charge-transfer (MICT). Mechanistic studies indicate that copper/iodine-to-ligand and intraligand charge transfer states dominate the radiative decay processes. Compared to the control compound Cu_4_I_4_[DBFDP]_2_, the incorporation of donor groups in the ligands of Cu_4_I_4_[DDPACDBFDP]_2_ results in a greater than 20-fold enhancement in radioluminescence emission efficiency and a 14-fold increase in photoluminescence efficiency. In the same year, Omar's team reported two Cu_4_I_4_ clusters, Cu_4_I_4_(bzpy)_4_ and Cu_4_I_4_(*t*bpy)_4_ (bzpy = 4-benzylpyridine, *t*bpy = 4-*tert*-butylpyridine), which exhibit broad emission bands, high PLQY (>85%), and large Stokes shifts.^13^ The structures of Cu_4_I_4_(bzpy)_4_ and Cu_4_I_4_(*t*bpy)_4_ are shown in Fig. 11d. To fabricate high-quality scintillation screens, the colloidal cluster microcrystals were compacted into uniform films, which were readily produced *via* vacuum filtration at room temperature. The scintillation screens based on Cu_4_I_4_(bzpy)_4_ and Cu_4_I_4_(*t*bpy)_4_ exhibited outstanding scintillation performance, achieving high LYs of approximately 36 900 photons per MeV and 30 800 photons per MeV, respectively, along with low detection limits of 102.1 nGy s^−1^ and 137.2 nGy s^−1^ (Fig. 11e).

**Fig. 12:**
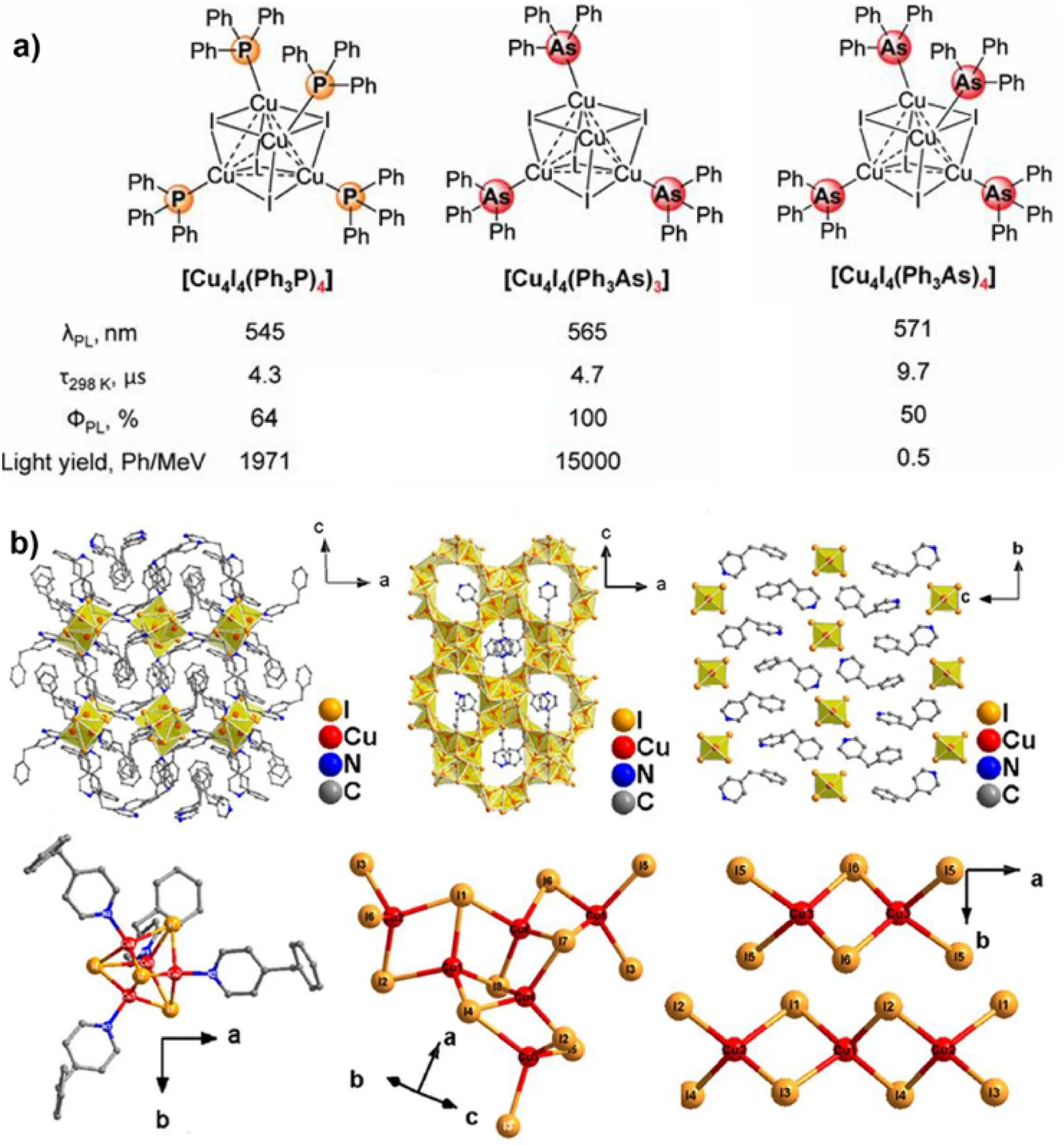
(a) Comparison of photoluminescence and XEL properties of Cu_4_I_4_(Ph_3_P)_4_, Cu_4_I_4_(Ph_3_As)_3_, and Cu_4_I_4_(Ph_3_As)_4_.^32^ Reproduced from ref. 32 with permission from Wiley, copyright 2024. (b) Crystal structures of Cu_4_I_4_(4-bzpy)_4_ (left), Cu_6_I_8_(4-bzpy)_2_ (middle) and Cu_3_I_6_(4-bzpy)_3_ (right).^33^ Reproduced from ref. 33 with permission from Royal Society of Chemistry, copyright 2024.

In 2024, Alexander's team reported a series of Cu_4_I_4_ clusters with the general formula Cu_4_I_4_(R_3_As)_3_L (L = none or nitrile), which exhibit intense yellow-green room-temperature phosphorescence with PLQY up to 100% and short decay times ranging from 4.4 µs to 4.9 µs.^32^ Among these clusters, the one without a nitrile ligand (Cu_4_I_4_(R_3_As)_3_) exhibits superior XEL properties, demonstrating a detection limit of 18.1 nGy s^−1^ and a highly linear dose-rate response over an exceptionally wide range (0–640 µGy s^−1^). Surprisingly, Cu_4_I_4_(Ph_3_As)_3_ and its structurally related tetrarsenic congener, Cu_4_I_4_(Ph_3_As)_4_, despite their similar molecular structures, exhibit markedly different emission properties. The PLQY of Cu_4_I_4_(Ph_3_As)_4_ is only 50%, which is much lower than that of Cu_4_I_4_(Ph_3_As)_3_. Meanwhile, the decay time of Cu_4_I_4_(Ph_3_As)_4_ (9.7 µs) is longer than that of Cu_4_I_4_(Ph_3_As)_3_ (4.7 µs). Furthermore, compared with its phosphine analog Cu_4_I_4_(PPh_3_)_4_, which exhibits a PLQY of 64% and a decay time of 4.3 µs, Cu_4_I_4_(Ph_3_As)_3_ demonstrates a significantly higher LY of 15 000 photons per MeV (Fig. 12a). This enhancement can be attributed to the greater X-ray absorption capability of the arsenic atom relative to the phosphorus atom. Subsequently, Tao's team reported a copper iodide cluster, Cu_4_I_4_(4-bzpy)_4_ (4-bzpy = 4-benzylpyridine).^33^Cu_4_I_4_(4-bzpy)_4_ exhibits broadband emission with a near-unity PLQY of approximately 99.7% and a significantly large Stokes shift of 216 nm. Meanwhile, (4-bzpy)_4_Cu_4_I_4_ exhibits a pronounced X-ray response with a LY of 60 948 photons per MeV, significantly surpassing that of the control copper iodide clusters, Cu_6_I_8_(4-bzpy)_2_ (5353 photons per MeV) and Cu_3_I_6_(4-bzpy)_3_ (7011 photons per MeV), which were synthesized using the same ligands but formed distinct molecular structures (Fig. 12b). The above two samples indicate that the XEL properties of copper iodide clusters are closely related to their molecular structures, even when their molecular compositions are similar.

**Fig. 13:**
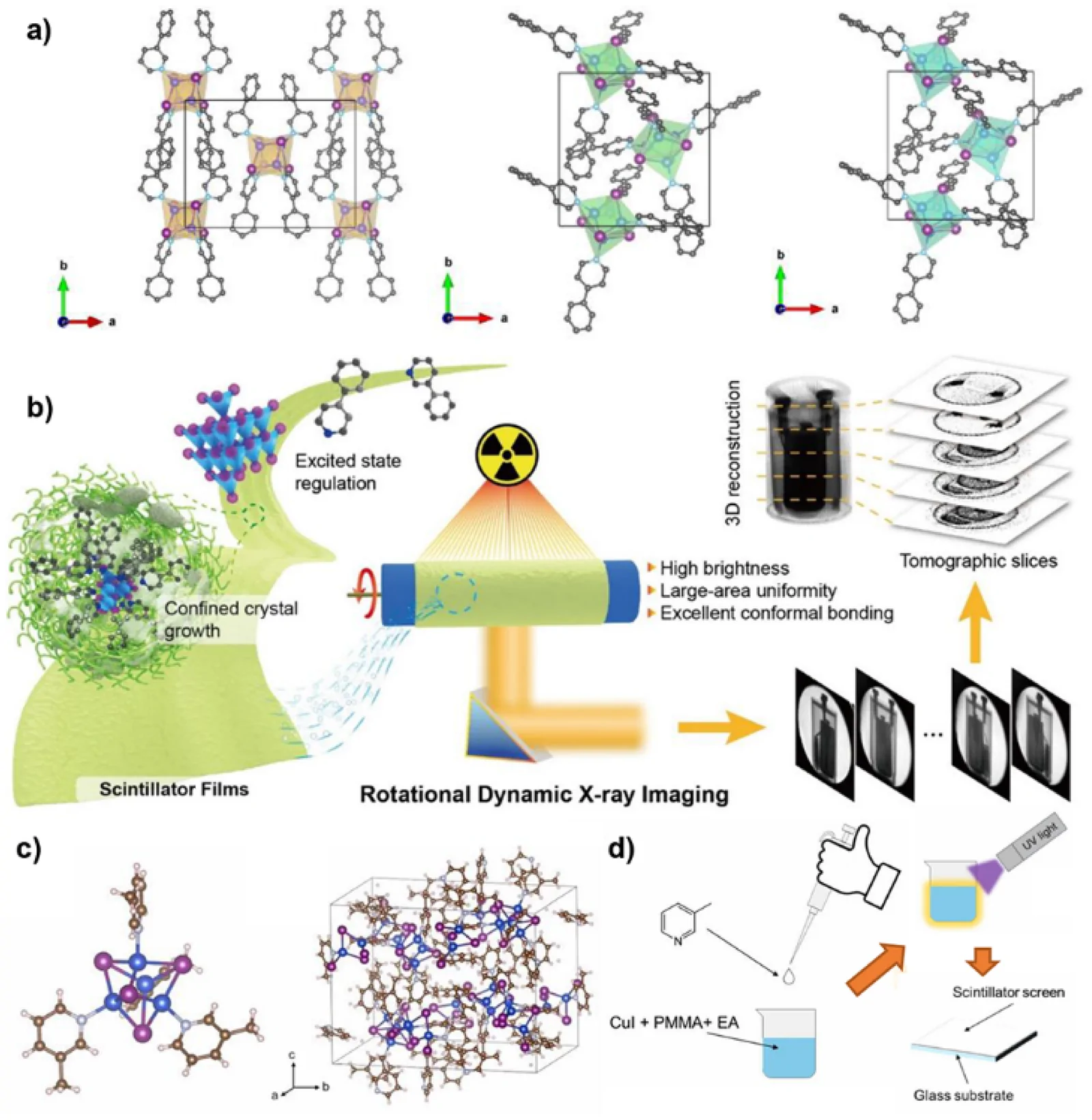
(a) Crystal structures of Cu_4_I_4_(3-ppy)_4_, Cu_4_I_4_(4-ppy)_4_-1, and Cu_4_I_4_(4-ppy)_4_-2 (from left to right). (b) Schematic illustration of the design of a copper iodide cluster-based scintillator film for rotational dynamic X-ray imaging and three-dimensional reconstruction of nonplanar objects.^34^ Reproduced from ref. 34 with permission from American Chemical Society, copyright 2025. (c) Crystal structure and unit cell packing diagram of Cu_4_I_4_(3-pic)_4_. (d) Schematic illustration of the *in situ* synthesis of Cu_4_I_4_(3-pic)_4_ ink and preparation of a scintillator screen.^35^ Reproduced from ref. 35 with permission from Royal Society of Chemistry, copyright 2025.

In 2025, Zhao's team employed electron-rich ligands to modulate the excited-state dynamics and suppress nonradiative transitions in Cu_4_I_4_ clusters.^34^ Under PVP micelle confinement, colloidal Cu_4_I_4_ cluster-derived microcrystals were synthesized. As illustrated in Fig. 13a, three copper iodide clusters, Cu_4_I_4_(3-ppy)_4_, Cu_4_I_4_(4-ppy)_4_-1, and Cu_4_I_4_(4-ppy)_4_-2, were synthesized *via* a simple solution diffusion method using 3-phenylpyridine (3-ppy) and 4-phenylpyridine (4-ppy) as ligands. Through the synergistic integration of ligand engineering, PVP micelle templating, and precise microelectronic printing control, uniform scintillator films were fabricated, enabling high-resolution X-ray imaging with a spatial resolution exceeding 20 lp per mm. These films demonstrate excellent performance in real-time dynamic X-ray imaging and three-dimensional reconstruction of nonplanar objects (Fig. 13b). In the same year, Wu's team reported the synthesis of a Cu_4_I_4_ cluster of Cu_4_I_4_(3-pic)_4_ (3-pic = 3-picoline) through a straightforward solution-based approach.^35^ Upon UV excitation, Cu_4_I_4_(3-pic)_4_ exhibits CC emission with a PLQY of 89.25%. Single-crystal structural analysis revealed the densely packed atomic arrangements in Cu_4_I_4_(3-pic)_4_, suggesting strong X-ray attenuation capability (Fig. 13c). Additionally, an *in situ* synthesis approach was developed to produce highly emissive Cu_4_I_4_(3-pic)_4_ scintillator ink (Fig. 13d). The resulting scintillator screen exhibited excellent performance, characterized by high LY, superior spatial resolution, and a low detection limit.

**Fig. 14:**
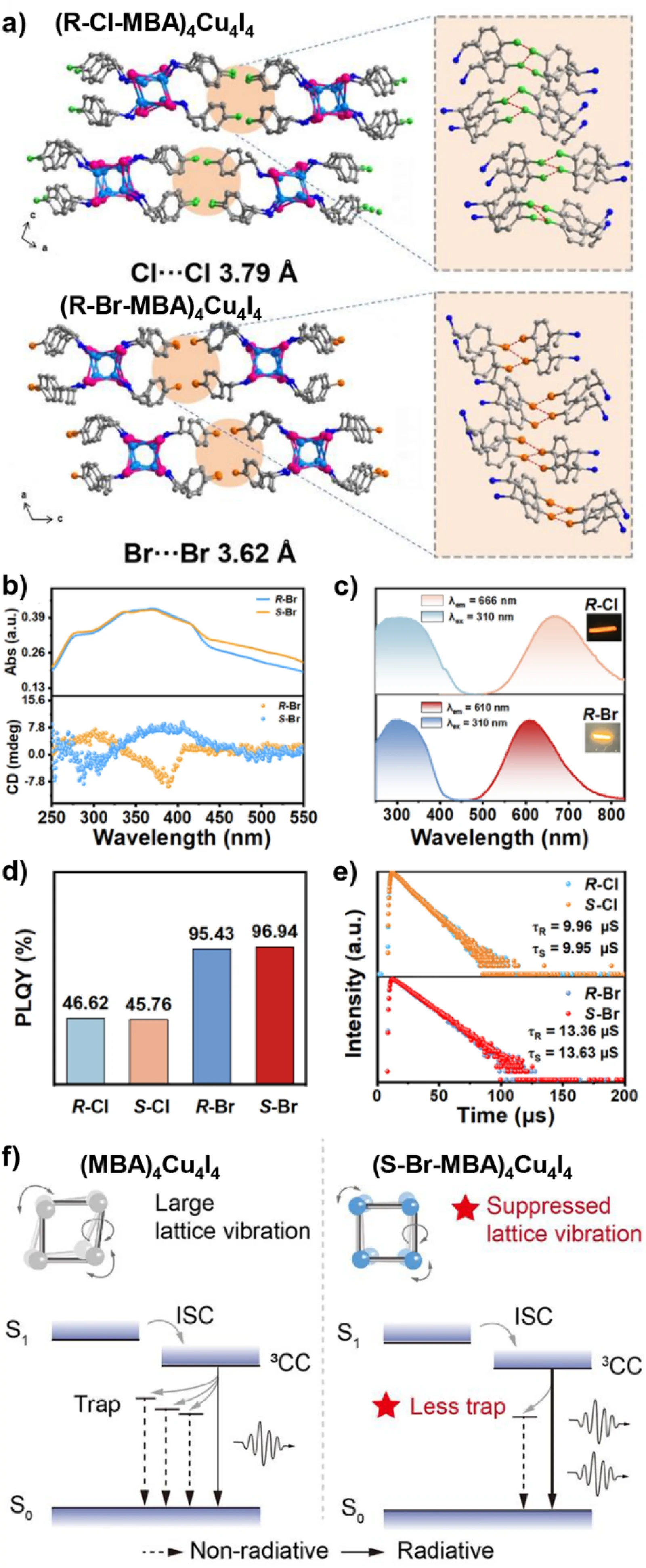
(a) Schematic diagram of halogen bonding interactions and the resulting rigid crystal structure of (*R*/*S*-Cl-MBA)_4_Cu_4_I_4_ and (*R*/*S*-Br-MBA)_4_Cu_4_I_4_. (b) CD and absorption spectra of *R*/*S*-Br. (c) Photoluminescence excitation and emission spectra of (*R*/*S*-Cl-MBA)_4_Cu_4_I_4_ and (*R*/*S*-Br-MBA)_4_Cu_4_I_4_. (d) PLQY of (*R*/*S*-Cl-MBA)_4_Cu_4_I_4_ and (*R*/*S*-Br-MBA)_4_Cu_4_I_4_. (e) Photoluminescence decay profiles of (*R*/*S*-Cl-MBA)_4_Cu_4_I_4_ (*λ*_em_ = 666 nm) and (*R*/*S*-Br-MBA)_4_Cu_4_I_4_ (*λ*_em_ = 610 nm) single crystals.^36^ Reproduced from ref. 36 with permission from Royal Society of Chemistry, copyright 2025. (f) Schematic diagram of the scintillation mechanism. In contrast to (MBA)_4_Cu_4_I_4_, (*S*-Br-MBA)_4_Cu_4_I_4_ shows suppressed lattice vibrations and fewer trap states, promoting efficient radiative decay.^37^ Reproduced from ref. 37 with permission from Wiley, copyright 2025.

Chiral ligands have also been employed in the construction of Cu_4_I_4_ cluster scintillators. In 2025, Pang and coworkers synthesized two zero-dimensional Cu_4_I_4_ clusters using chiral (*R*/*S*)-4-chloro/bromo-α-methylbenzylamine (*R*/*S*-X-MBA, X = Cl, Br) as ligands (Fig. 14a). The clusters of (*R*/*S*-Cl-MBA)_4_Cu_4_I_4_ and (*R*/*S*-Br-MBA)_4_Cu_4_I_4_ are prepared through a straightforward room-temperature solvent-diffusion method.^36^ Under 310 nm excitation, (*R*/*S*-Cl-MBA)_4_Cu_4_I_4_ and (*R*/*S*-Br-MBA)_4_Cu_4_I_4_ exhibit efficient orange and yellow luminescence, respectively, characterized by large Stokes shifts and symmetric chiral CD signals (Fig. 14b and c). It is noted that compared to (*R*/*S*-Cl-MBA)_4_Cu_4_I_4_ with a PLQY of 46%, a near-unity PLQY is observed for (*R*/*S*-Br-MBA)_4_Cu_4_I_4_, along with a longer photoluminescence lifetime of 13.5 µs (Fig. 14d and e). This enhanced emission efficiency is attributed to intermolecular halogen–halogen interactions, which significantly increase lattice rigidity and suppress nonradiative recombination of triplet excitons. This feature makes (*R*/*S*-Br-MBA)_4_Cu_4_I_4_ suitable for X-ray scintillator applications. Liu's research further compared the scintillation properties of (*S*-Br-MBA)_4_Cu_4_I_4_ with its halogen-free counterpart, (MBA)_4_Cu_4_I_4_, further demonstrating that the halogen bonding improves lattice symmetry and rigidity, effectively suppressing electron–phonon coupling and defect trapping in the clusters (Fig. 14f).^37^ Meanwhile, they also improve the preparation method of the (*S*-Br-MBA)_4_Cu_4_I_4_ scintillator film by the blade-coated method, increasing the X-ray imaging resolution to 22 lp per mm.

**Fig. 15:**
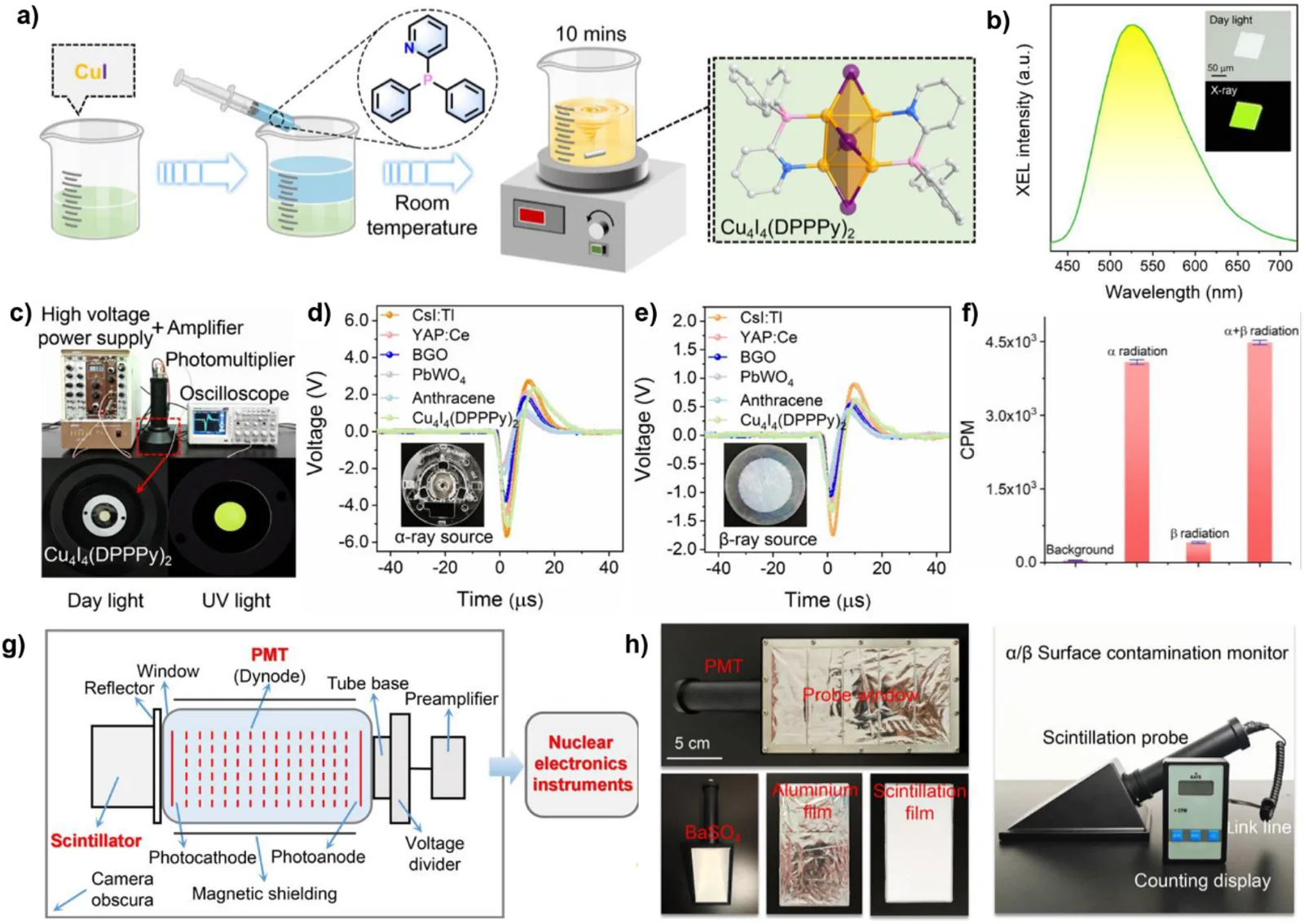
(a) Synthesis of Cu_4_I_4_(DPPPy)_2_. (b) XEL spectrum of Cu_4_I_4_(DPPPy)_2_. Inset: Photographs of the crystal under daylight and X-ray irradiation. (c) Entity structure diagram of the α/β particle detection system. Response signals of Cu_4_I_4_(DPPPy)_2_ and commercial scintillators to (d) α particles and (e) β particles under the same conditions. (f) The count display of the α/β surface contamination monitor in different radiation environments. (g) Structure diagram of the scintillator detector. (h) Structure diagram of the scintillation probe and the α/β surface contamination monitor.^38^ Reproduced from ref. 38 with permission from Wiley, copyright 2025.

Recently, Li's group reported a copper cluster, Cu_4_I_4_(DPPPy)_2_ (DPPPy = 2-(diphenylphosphino)pyridine), which demonstrates remarkable water–oxygen stability. Cu_4_I_4_(DPPPy)_2_ can be synthesized through a straightforward one-pot procedure conducted at ambient temperature (Fig. 15a).^38^Cu_4_I_4_(DPPPy)_2_ not only exhibits excellent XEL but also shows a highly sensitive scintillation response to α/β particles (Fig. 15b–f). By integrating Cu_4_I_4_(DPPPy)_2_ with a photomultiplier tube (PMT) and nuclear electronics, they have successfully developed an α/β surface contamination monitor capable of sensitively detecting elevated levels of α and β radiation in real-world environments (Fig. 15g and h). Under identical experimental conditions, both the detection frequency and signal intensity achieved with Cu_4_I_4_(DPPPy)_2_ significantly exceed those of commercial scintillators such as YAP:Ce, BGO, PbWO_4_, and anthracene, underscoring the potential of metal clusters for applications in low-dose environmental radiation detection.

**Fig. 16:**
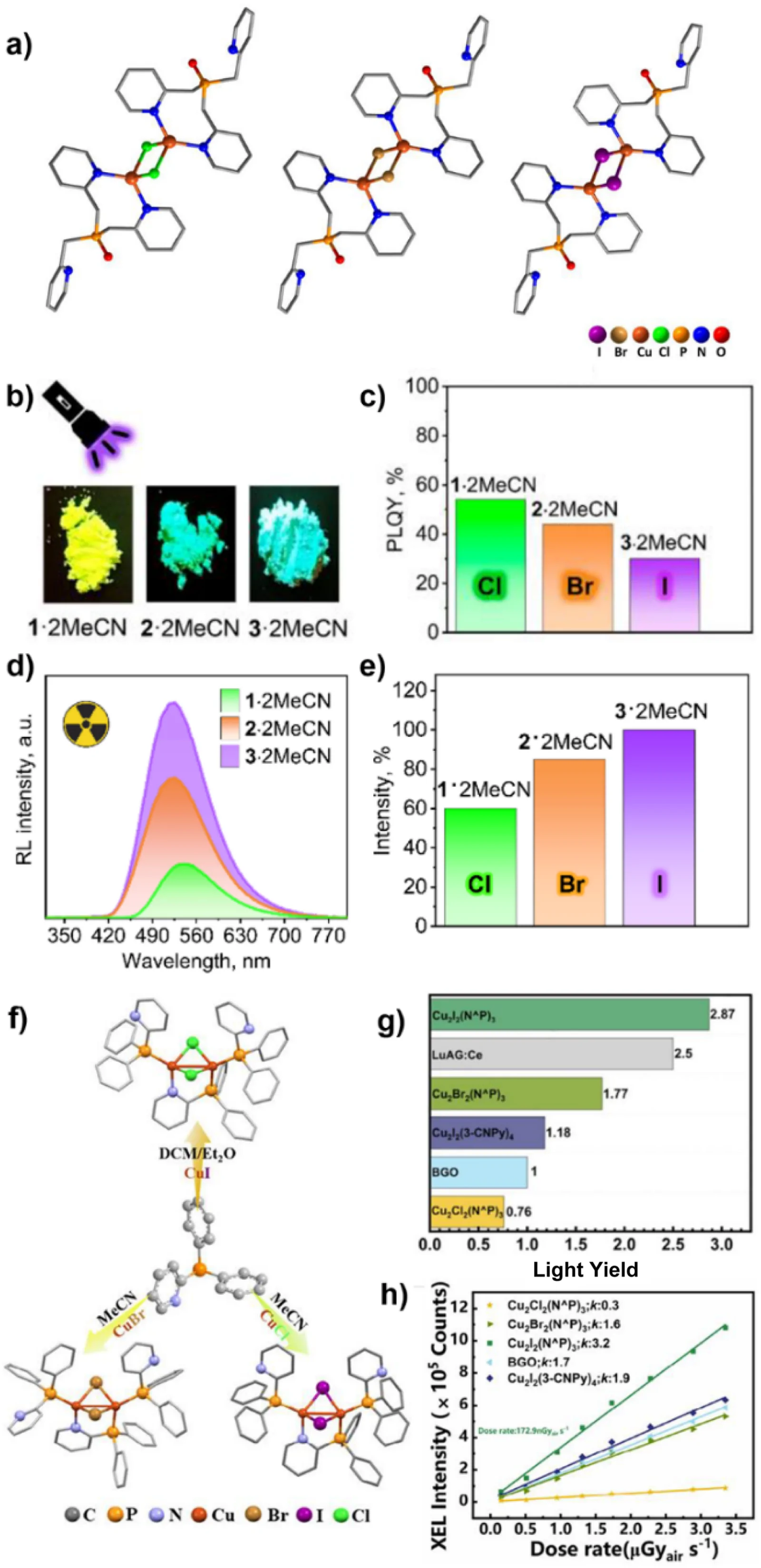
(a) Crystal structures of Cu_2_X_2_(Pic_3_PO)_2_ (b) Photographs of UV irradiated Cu_2_X_2_(Pic_3_PO)_2_. (c) PLQYs of Cu_2_X_2_(Pic_3_PO)_2_. (d) XEL spectra of Cu_2_X_2_(Pic_3_PO)_2_. (e) Relative XEL intensity of Cu_2_X_2_(Pic_3_PO)_2_.^11^ Reproduced from ref. 11 with permission from Multidisciplinary Digital Publishing Institute, copyright 2023. (f) The synthesis route and crystal structure of Cu_2_X_2_(NP)_3_. (g) LY comparison of Cu_2_X_2_(NP)_3_ with commercial scintillators. (h) Linear response of Cu_2_X_2_(NP)_3_ to X-ray dose rate.^39^ Reproduced from ref. 39 with permission from American Chemical Society, copyright 2026.

Another important kind of copper iodide cluster scintillator is the one with a Cu_2_I_2_ core. Although the X-ray absorption performance of the Cu_2_I_2_ core is weaker than that of the Cu_4_I_4_ core, the XEL performance of Cu_2_I_2_ clusters is comparable with that of Cu_4_I_4_ clusters. For example, the three TADF copper halide clusters, Cu_2_X_2_(Dppy)(F-PPh_3_)_2_ (X = Cl, Br, I), mentioned above are typical Cu_2_I_2_ clusters with excellent scintillation properties (Fig. 4).^12^ The LYs of Cu_2_I_2_(Dppy)(F-PPh_3_)_2_ and Cu_2_Br_2_(Dppy)(F-PPh_3_)_2_ were much higher than those of the reported phosphorescent Cu_4_I_4_ clusters (Cu_4_I_4_(DBA)_4_ and Cu_4_I_4_py_4_).^9,14^ These results indicate that the enhanced LY arises from the synergistic effect of high X-ray absorption by heavy halogen atoms and efficient harvesting of both singlet and triplet excitons *via* TADF mechanisms. In 2025, Artemev's group reported three copper halide clusters, Cu_2_X_2_(Pic_3_PO)_2_ (X = Cl, Br, I, and Pic_3_PO = tris(pyridin-2-ylmethyl)phosphine oxide) (Fig. 16a).^11^ These clusters exhibit TADF with emission wavelengths ranging from 485 nm to 545 nm. In the TADF process, the halide effect manifests as enhanced emission intensity and a bathochromic shift in the emission wavelength. Consequently, the photoluminescence intensity follows the order Cu_2_I_2_(Pic_3_PO)_2_ < Cu_2_Br_2_(Pic_3_PO)_2_ < Cu_2_Cl_2_(Pic_3_PO)_2_ (Fig. 16b). In contrast, the XEL intensity of the clusters exhibits an inverse trend, Cu_2_I_2_(Pic_3_PO)_2_ > Cu_2_Br_2_(Pic_3_PO)_2_ > Cu_2_Cl_2_(Pic_3_PO)_2_, because of the higher X-ray absorption efficiency of heavier halogen atoms (Fig. 16c).

Similar influence of heavy halogen atoms on XEL properties of copper iodide cluster scintillators was reported by Liu and coworkers.^39^ As shown in Fig. 16d, three copper iodide clusters of Cu_2_X_2_(NP)_3_ (X = Cl, Br, I, NP = diphenyl-2-pyridylphosphine) were synthesized. Among these clusters, the one with the heaviest halogen atom (Cu_2_I_2_(NP)_3_) exhibits the best XEL performance, which shows a LY of 28 700 photons per MeV and a detection limit of 172.9 nGy s^−1^ (Fig. 16e).

**Fig. 17:**
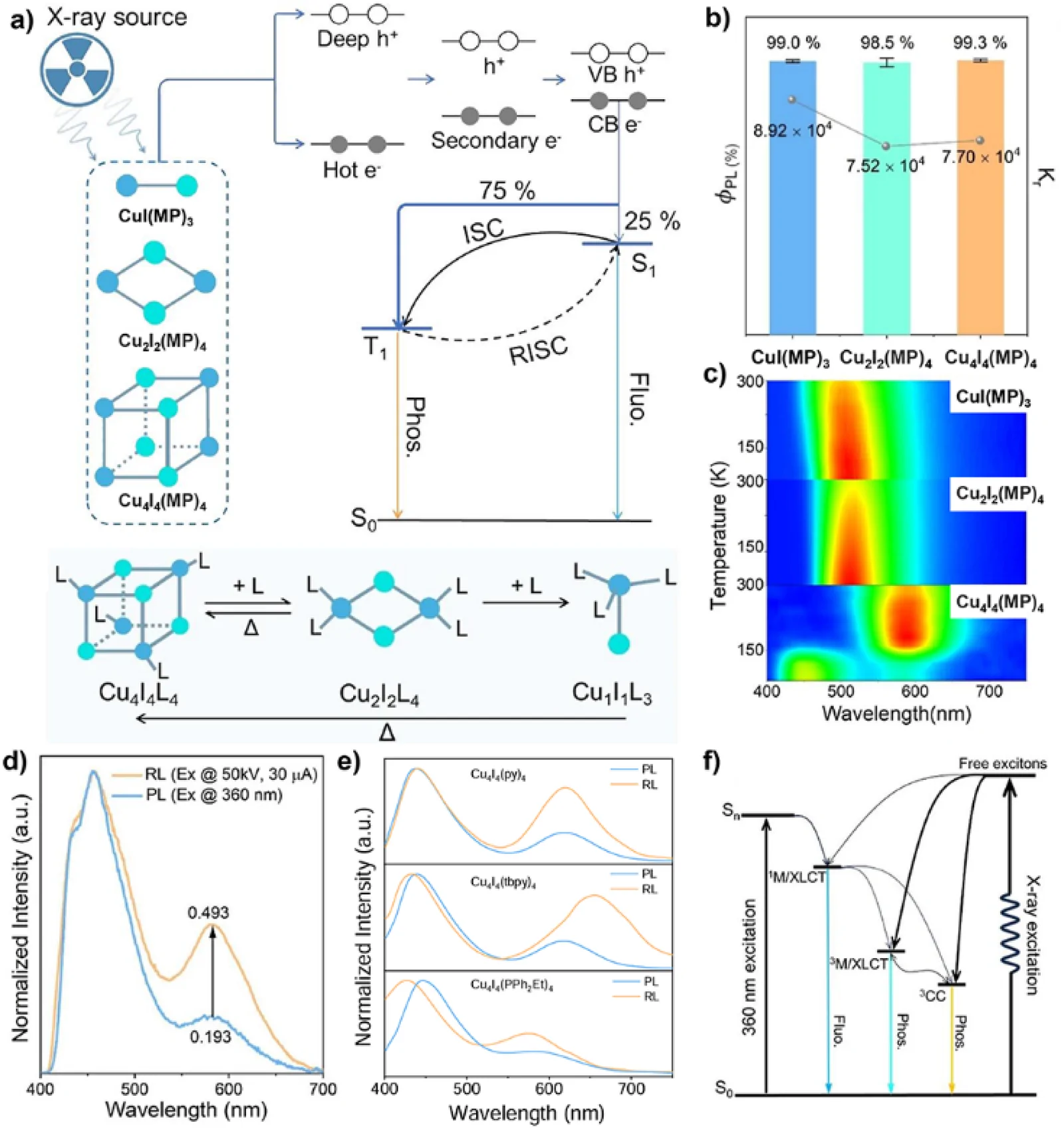
(a) Schematic illustration of the scintillation process in copper iodide clusters with distinct core geometries. (b) PLQY and corresponding radiative rate constants of Cu_1_I_1_(MP)_3_, Cu_2_I_2_(MP)_4_ and Cu_4_I_4_(MP)_4_. (c) Temperature-dependent radioluminescence spectra of Cu_1_I_1_(MP)_3_, Cu_2_I_2_(MP)_4_ and Cu_4_I_4_(MP)_4_. (d) Normalized photoluminescence and radioluminescence spectra of Cu_4_I_4_(MP)_4_ at 80 K. (e) Normalized photoluminescence and radioluminescence spectra of Cu_4_I_4_(py)_4_, Cu_4_I_4_(*t*bpy)_4_ and Cu_4_I_4_(PPh_2_Et)_4_ at 80 K. (f) Schematic representation of the exciton population across multiple excited states under X-ray and UV light excitation.^40^ Reproduced from ref. 40 with permission from American Chemical Society, copyright 2025.

In 2025, Omar and coworkers reported a unified ligand strategy to construct a series of zero-dimensional copper iodide clusters, including a Cu_1_I_1_ monomer, Cu_1_I_1_(MP)_3_, a Cu_2_I_2_ rhomboid dimer, Cu_2_I_2_(MP)_4_, and a Cu_4_I_4_ cubane tetramer, Cu_4_I_4_(MP)_4_ (MP = 3-methylpyridine) (Fig. 17a).^40^ All three copper iodide clusters exhibit TADF characteristics with near-unity PLQY (Fig. 17b). Based on these clusters, the study systematically investigates how the core architecture governs radioluminescence behavior and efficiency beyond PLQY. Their results demonstrate that core geometry significantly influences both thermal stability and exciton relaxation pathways. As shown in Fig. 17c, under X-ray excitation, all three compounds exhibit emission profiles that are similar to those observed in photoluminescence measurements. Cu_1_I_1_(MP)_3_ and Cu_2_I_2_(MP)_4_ display a gradual decrease in single-peak emission intensity over the temperature range from 80 K to 300 K. In contrast, Cu_4_I_4_(MP)_4_ exhibits a distinct evolution from high-energy to low-energy emission with increasing temperature, indicating a temperature-dependent shift in the dominant emissive state. These results confirm the involvement of the same excited states observed in photoluminescence, namely, a ^1/3^(M/X)LCT-based TADF process in Cu_1_I_1_(MP)_3_ and Cu_2_I_2_(MP)_4_, and a thermally driven transition from ^3^(M/X)LCT to ^3^CC emission in Cu_4_I_4_(MP)_4_. As shown in Fig. 17d, Cu_4_I_4_(MP)_4_ exhibits dual emission under both X-ray and UV light excitation at 80 K. Notably, a significantly stronger low-energy emission was observed under X-ray excitation, indicating that a larger fraction of excitons undergoes radiative relaxation to the ground state *via* the ^3^CC state, in addition to those originating from intersystem crossing from the ^3^(M/X)LCT state. This phenomenon is further confirmed in other well-studied dual-excited-state copper iodide cluster scintillators (Fig. 17e) such as Cu_4_I_4_(py)_4_,^41^Cu_4_I_4_(*t*bpy)_4_,^13^ and Cu_4_I_4_(PPh_2_Et)_4_,^42^ indicating that it is a general feature of copper iodide cluster scintillators. Based on these findings, they propose a multistate radioluminescence mechanism (Fig. 17f). Under X-ray excitation, the free excitons occupying the ^1^(M/X)LCT and ^3^(M/X)LCT states decay through pathways analogous to those observed under UV excitation, resulting in similar high-energy emission in the photoluminescence and radioluminescence spectra. Meanwhile, a significant fraction of free excitons directly populate the ^3^CC state under X-ray excitation (this process is not operative under UV excitation), giving rise to markedly enhanced low-energy emission. Subsequently, excitons in the ^1^(M/X)LCT state undergo intersystem crossing (ISC) to the ^3^(M/X)LCT state or the lower-lying ^3^CC state. Excitons in the ^3^(M/X)LCT state partially relax to the ^3^CC state before radiatively decaying to the ground state from both triplet states. Elevated temperature facilitates this process.

**Fig. 18:**
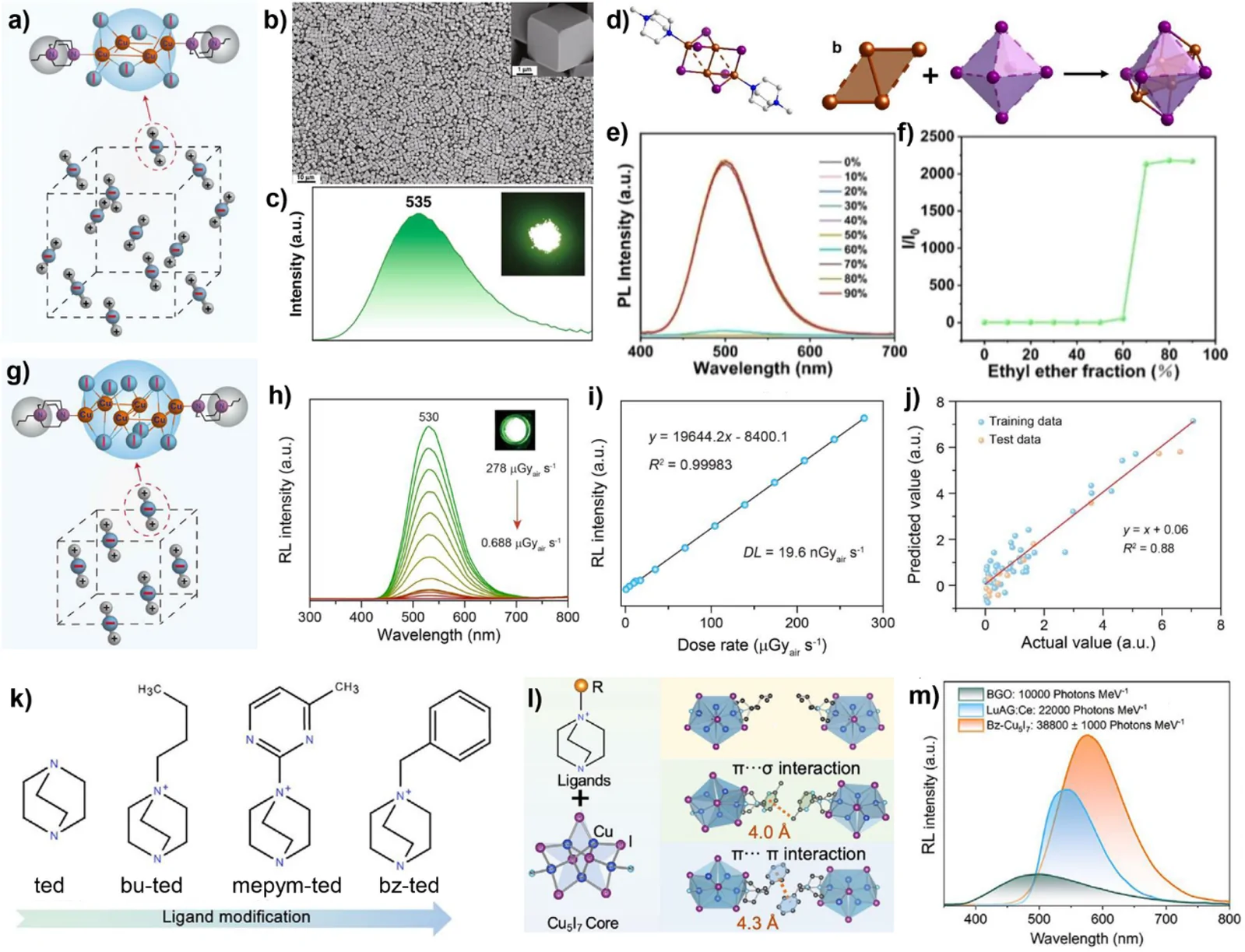
(a) Crystal structure of Cu_4_I_6_(pr-ted)_2_. (b) SEM image of the hot-injection-prepared Cu_4_I_6_(pr-ted)_2_ microcubes. (c) Photoluminescence spectra of Cu_4_I_6_(pr-ted)_2_.^15^ Reproduced from ref. 15, with permission from Springer Nature, copyright 2023. (d) Crystal structure of Cu_4_I_6_(me-ted)_2_. (e) Photoluminescence spectra of Cu_4_I_6_(me-ted)_2_ in mixed solvents with different water fractions. (f) Relative photoluminescence intensity of Cu_4_I_6_(me-ted)_2_ as a function of water fractions.^43^ Reproduced from ref. 43, with permission from Elsevier, copyright 2026. (g) Crystal structure of Cu_6_I_8_(bu-ted)_2_. (h) XEL spectra of Cu_6_I_8_(bu-ted)_2_ under different excitation dose rates. (i) XEL intensity of Cu_6_I_8_(bu-ted)_2_ as a function of excitation dose. (j) Prediction of light output area for the dataset of Cu(i)–I cluster scintillators.^44^ Reproduced from ref. 44, with permission from Wiley, copyright 2024. (k) Ligand modifications from the spherical ligand of ted to targeted ligands. (l) The structures of Cu_5_I_7_(bu-ted)_2_, Cu_5_I_7_(mepym-ted)_2_ and Cu_5_I_7_(bz-ted)_2_. (m) Comparison of XEL spectra of Cu_5_I_7_(bz-ted)_2_, BGO and LuAG:Ce.^45^ Reproduced from ref. 45 with permission from Wiley, copyright 2025.

Besides Cu_4_I_4_ and Cu_2_I_2_, those with anion cores ([Cu_*m*_I_*n*_]^(*n*−*m*)−^, *n* > *m*) are also a common type of copper iodide cluster scintillators. For example, Huang's group reported a copper iodide cluster microcube containing a [Cu_4_I_6_]^2−^ core and two cationic ligands of 1-propyl-1,4-diazabicyclo[2.2.2]octan-1-ium (pr-ted) (Fig. 18a).^15^Cu_4_I_6_(pr-ted)_2_ can be synthesized *via* a hot-injection method followed by thermal annealing at 200 °C for 1.5 h under a N_2_ atmosphere. The as-prepared Cu_4_I_6_(pr-ted)_2_ microcubes exhibit strong green XEL centered at 535 nm (Fig. 18b and c), which is identical to phosphorescence observed under 365 nm excitation. These copper iodide cluster microcubes exhibit remarkable stability toward both water and X-ray irradiation and have been successfully employed in the fabrication of flexible, high-performance composite scintillators for small-animal X-ray imaging under both static and dynamic conditions. A similar copper iodide cluster was also investigated by Wang's group. As shown in Fig. 18d, using 1-methyl-1,4-diazabicyclo[2.2.2]octan-1-ium (me-ted) as a ligand, a Cu_4_I_6_(me-ted)_2_ cluster was prepared.^43^ The crystallization-induced emission enhancement (CIEE) properties of the Cu_4_I_6_(me-ted)_2_ cluster were investigated (Fig. 18e and f). Subsequently, Huang's group used a similar ligand, 1-butyl-1,4-diazabicyclo[2.2.2]octan-1-ium (bu-ted), to prepare another copper iodide cluster scintillator with a core consisting of [Cu_6_I_8_]^2−^ (Cu_6_I_8_(bu-ted)_2_), as shown in Fig. 18g.^44^ This cluster exhibits excellent XEL performance, enables a detection limit for X-rays of 19.6 nGy s^−1^ (Fig. 18h and i). More importantly, Cu_6_I_8_(bu-ted)_2_ was designed by a machine learning strategy. Their findings reveal that combining base learning models with fused features enhances model generalization, achieving a high determination coefficient of 0.88 (Fig. 18j), highlighting the significance of a machine learning-guided approach in accelerating the development of high-performance copper iodide cluster scintillators for practical applications. Further modification on the triethylenediamine (ted) ligand was carried out by Zhao and coworkers (Fig. 18k).^45^ As shown in Fig. 18l, they reported three copper iodide cluster scintillators, Cu_5_I_7_(bu-ted)_2_, Cu_5_I_7_(mepym-ted)_2_ and Cu_5_I_7_(bz-ted)_2_, by introducing π–π interactions to suppress the nonradiative pathways, achieving near-unity PLQY for Cu_5_I_7_(bz-ted)_2_. When incorporated into a flexible scintillator film, Cu_5_I_7_(bz-ted)_2_ demonstrates remarkable X-ray imaging performance, enabling a high spatial resolution exceeding 20 lp per mm and a low radiation dose of 71.2 nGy s^−1^ (Fig. 18m).

**Fig. 19:**
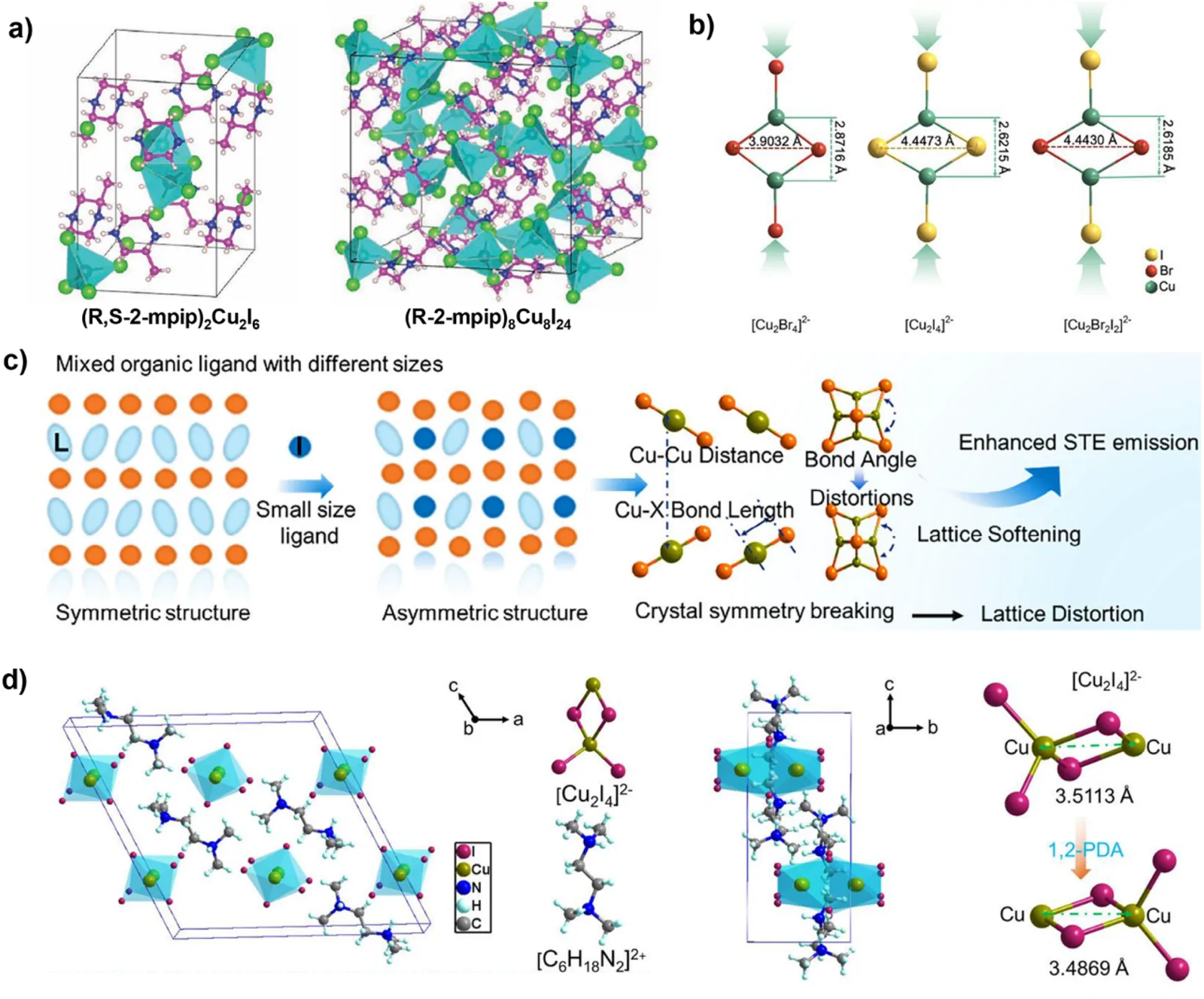
(a) Crystal structure of (*R*,*S*-2-mpip)_2_Cu_2_I_6_ and (*R*-2-mpip)_8_Cu_8_I_24_.^46^ Reproduced from ref. 46 with permission from Wiley, copyright 2023. (b) Illustration of the shorter Cu–Cu distance with the mixing of halogens.^47^ Reproduced from ref. 47 with permission from Wiley, copyright 2025. (c) The model of mixing organic ligands with the formation of crystal symmetry breaking and lattice distortion.^48^ Reproduced from ref. 48 with permission from Wiley, copyright 2025. (d) Crystal structure of (C_6_H_18_N_2_)Cu_2_I_4_ (8 : 2) and structure of [Cu_2_I_4_]^2−^ units before and after the addition of 1,2-PDA.^49^ Reproduced from ref. 49 with permission from Wiley, copyright 2025.

Other organic amine cations are also introduced into the construction of copper iodide cluster scintillators with anion cores, along with different strategies to improve their XEL properties. In 2023, Dang and coworkers reported a chirality-racemization strategy to construct a copper halogen cluster scintillator.^46^ A zero-dimensional copper iodide cluster scintillator, (*R*,*S*-2-mpip)_2_Cu_2_I_6_ (mpip = methylpiperazinium), was synthesized with equal stoichiometric ratios of *R*- and *S*-2-mpip ligands (Fig. 19a). (*R*,*S*-2-mpip)_2_Cu_2_I_6_ exhibits favourable photoluminescence and radioluminescence properties. In contrast, the copper iodide clusters synthesized with *R*-2-mpip ((*R*-2-mpip)_8_Cu_8_I_24_) or *S*-2-mpip ((*S*-2-mpip)_8_Cu_8_I_24_) exhibited no emission characteristics. In 2025, Ouyang and coworkers further proposed another strategy of halogen co-mixing (HCM) to develop copper halogen cluster scintillators.^47^ As shown in Fig. 19b, a zero-dimensional copper halogen cluster of [N(C_3_H_7_)_4_]_2_^+^[Cu_2_Br_2_I_2_]^2−^ was prepared with excellent XEL properties. The HCM structure effectively reduces the Cu–Cu distance and increases the lattice asymmetry. As a result, [N(C_3_H_7_)_4_]_2_^+^[Cu_2_Br_2_I_2_]^2−^ exhibits higher PLQY and LY than those of single-halogen control compounds [N(C_3_H_7_)_4_]_2_^+^[Cu_2_Br_4_]^2−^ and [N(C_3_H_7_)_4_]_2_^+^[Cu_2_I_4_]^2−^. Beside the HCM strategy, a mixed-ligand-induced crystal symmetry breaking and lattice distortion strategy was proposed by Ju's group, who used mixed amine ligands to construct a high-performance copper halogen cluster scintillator, (C_8_H_20_N)_1_(C_12_H_28_N)_1_Cu_4_Br_6_.^48^(C_8_H_20_N)_1_(C_12_H_28_N)_1_Cu_4_Br_6_ exhibits a near unity PLQY (99%) and high radioluminescence intensity. Mechanistic studies demonstrate that the mixed-ligand approach effectively modulates bond lengths and angles, thereby enhancing structural distortion while simultaneously promoting charge transfer to achieve improved luminescence (Fig. 19c). Recently, Ju and coworkers employed neutral 1,2-propanediamine (1,2-PDA) as a dual-function additive in the construction of a copper iodide cluster scintillator, (C_6_H_18_N_2_)Cu_2_I_4_ (C_6_H_18_N_2_^2+^ = *N*,*N*,*N*′,*N*′-tetramethylethylenediamine cation) (Fig. 19d).^49^ Mechanistic studies reveal that 1,2-PDA not only strengthens ionic interactions between the organic ligand and the copper iodide core, thereby promoting oriented aggregation at crystal interfaces, but also induces significant lattice distortion upon defect incorporation, enhancing ISC and intensifying electron–phonon coupling, which collectively boost self-trapped exciton emission.

**Fig. 20:**
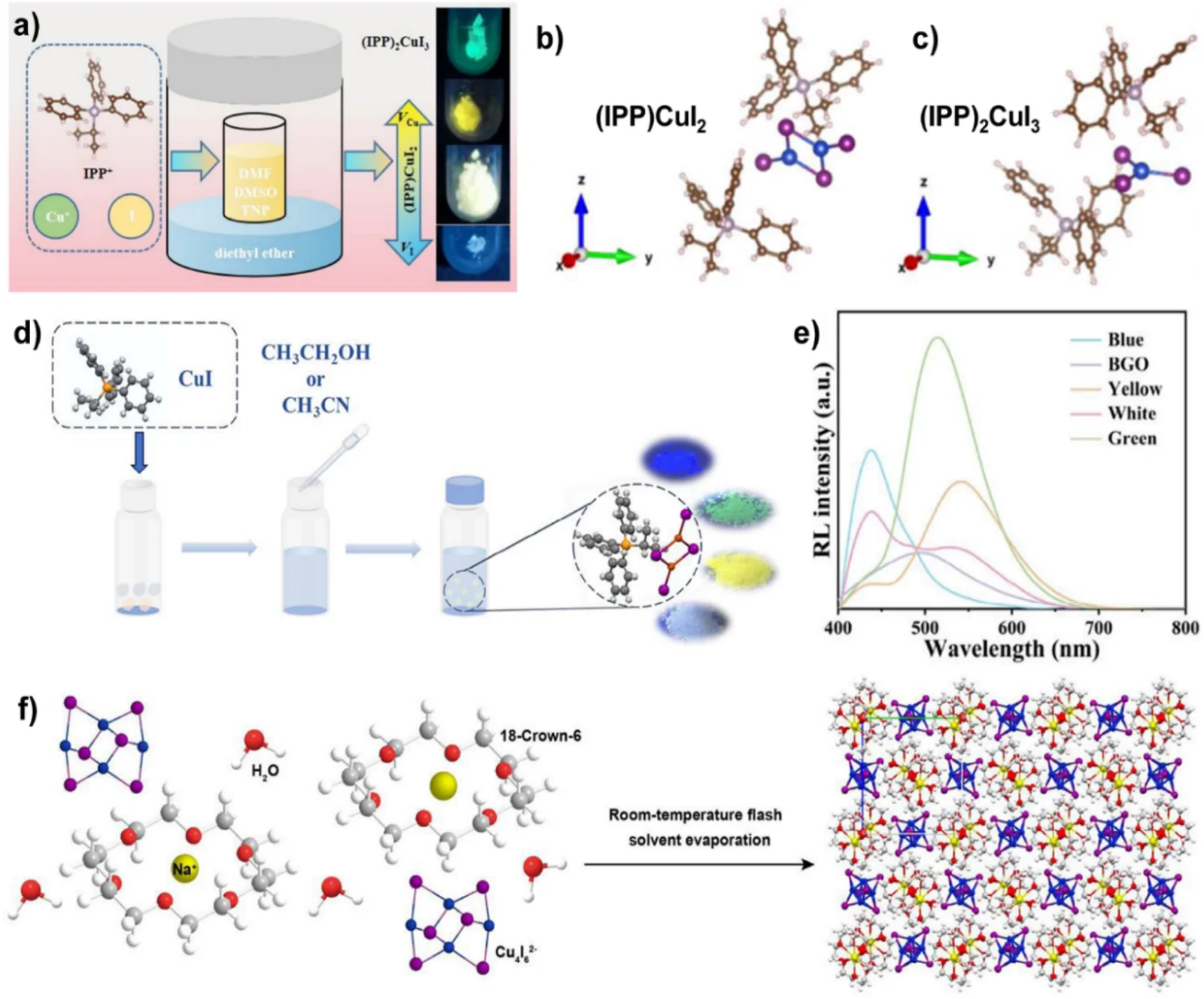
(a) Schematic diagram of the synthetic process of (IPP)CuI_2_ and (IPP)_2_CuI_3_. (b) Crystal structure of (IPP)CuI_2_. (c) Crystal structure of (IPP)_2_CuI_3_.^50^ Reproduced from ref. 50 with permission from Elsevier, copyright 2024. (d) The synthetic route of (IPP)CuI_2_ with different emission colors. (e) The XEL spectra of (IPP)CuI_2_ with different emission colors.^51^ Reproduced from ref. 51 with permission from Wiley, copyright 2024. (f) The synthesis process and crystal structure of (C_12_H_24_O_6_)_2_Na_2_(H_2_O)_3_Cu_4_I_6_.^52^ Reproduced from ref. 52 with permission from Springer Nature, copyright 2024.

Another example of copper iodide cluster scintillators with anion cores was reported by Tang's team, who prepared the copper iodide cluster scintillators, (IPP)CuI_2_ and (IPP)_2_CuI_3_ (IPPI = isopropyltriphenylphosphonium iodide), exhibiting tunable emission properties by simple component regulation (Fig. 20a).^50^ Varying the molar ratio of IPPI to CuI in reactants can modulate the luminescent behavior. As shown in Fig. 20b, the (IPP)CuI_2_ cluster exhibiting white-light emission consists of IPP^+^ cations and typical [Cu_2_I_4_]^2−^ dimers. In an isolated [Cu_2_I_4_]^2−^ dimer, each copper atom coordinates with three iodine atoms, forming two edge-sharing distorted trigonal planes. Periodically embedded with abundant IPP^+^ cations around isolated [Cu_2_I_4_]^2−^, (IPP)CuI_2_ adopts a zero-dimension structure. This dimer structure not only exhibits significant quantum confinement and strong electron–phonon coupling but also displays white-light emission arising from dual self-trapped exciton emissions. In contrast to the white-light-emitting crystal, the blue-green-emitting crystal contains isolated [CuI_3_]^2−^ moieties without edge-sharing, as illustrated in Fig. 20c. The (IPP)CuI_2_ scintillator with IPP : CuI = 1 : 0.75 shows the best scintillator properties, with a detection limit of 62 nGy s^−1^ and an LY of 75 794 photons per MeV. Further investigations of the (IPP)CuI_2_ scintillator were reported by Zhao's group.^51^ As shown in Fig. 20d, four (IPP)CuI_2_ clusters with different emission colors (blue, green, white, and yellow) are synthesized by varying the ratios of raw materials and types of reaction solvents. Mechanistic studies confirm that the different luminescent behaviours of the (IPP)CuI_2_ clusters are attributed to the vacancy defects originating from CuI. Huang's group showed that fast evaporation of a dichloromethane mixture of NaI, CuI, and 18-crown-6 led to the flash synthesis of rod-shaped (C_12_H_24_O_6_)_2_Na_2_(H_2_O)_3_Cu_4_I_6_ scintillators with a yield of 95% (Fig. 20e).^52^ They investigated the role of 18-crown-6 in the synthesis of (C_12_H_24_O_6_)_2_Na_2_(H_2_O)_3_Cu_4_I_6_ and found that the presence of crown ethers facilitates coordination with Na^+^ ions to form a positively charged complex, thereby enhancing the solubility of NaI and enabling the subsequent formation of [Cu_4_I_6_]^2−^ anions. The two Na^+^-coordinated crown ethers interconnect with each other, assisted by two water molecules, promoting the formation of secondary building units that effectively isolate [Cu_4_I_6_]^2−^ anions within the (C_12_H_24_O_6_)_2_Na_2_(H_2_O)_3_Cu_4_I_6_ crystal. The as-prepared (C_12_H_24_O_6_)_2_Na_2_(H_2_O)_3_Cu_4_I_6_ shows a high sensitivity towards X-rays, with a detection limit of 44 nGy s^−1^.

**Fig. 21:**
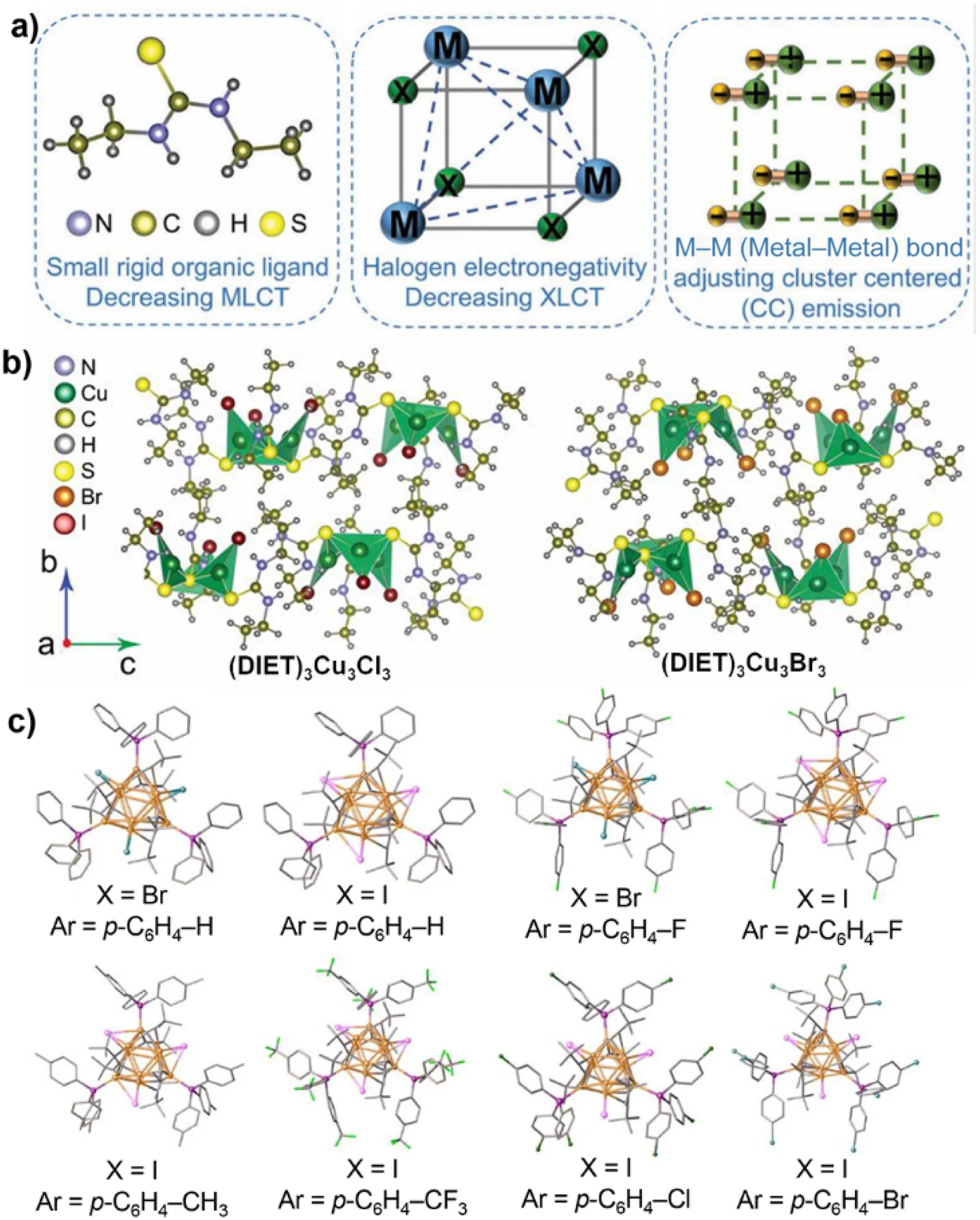
(a) Illustration of the structure designing principle for promoting single channel photon emission. (b) Crystal structures of (DIET)_3_Cu_3_Cl_3_ and (DIET)_3_Cu_3_Br_3_.^53^ Reproduced from ref. 53 with permission from Wiley, copyright 2022. (c) Crystal structures of Cu_9_X_3_(*^t^*BuC≡C)_9_(PAr_3_)_3_.^54^ Reproduced from ref. 54 with permission from Wiley and Science China Press, copyright 2025.

Recently, more copper halide cluster scintillators with different cores have been reported. Xia and coworkers reported two zero-dimensional copper halide clusters, (DIET)_3_Cu_3_X_3_ (DIET = 1,3-diethyl-2-thiourea, X = Cl, Br), which exhibit single-channel photoluminescence originating from the ^3^CC emission, thereby enhancing scintillation performance (Fig. 21a).^53^ The DIET ligands coordinated to copper ions not only establish direct Cu–L bonds but also induce the formation of a disordered Cu_3_X_3_ cluster, leading to enhanced X-ray absorption (Fig. 21b). The (DIET)_3_Cu_3_Br_3_ cluster, featuring a halogen with lower electronegativity, effectively suppresses XLCT emission, thereby promoting enhanced PLQY (69%) and superior scintillation performance. (DIET)_3_Cu_3_Br_3_ exhibits a LY of 20 000 ± 700 photons per MeV, along with a low detection limit of 189 nGy s^−1^, demonstrating its potential for high-sensitivity radiation detection. Lu and coworkers reported eight distinct propeller-like alkynyl copper clusters (Cu_9_X_3_(*^t^*BuC≡C)_9_(PAr_3_)_3_, X = Br, Ar = *p*-C_6_H_4_–R, R = H, F; X = I, Ar = *p*-C_6_H_4_–R, R = H, F, Cl, Br, CH_3_, CF_3_), each featuring a Cu_6_ core encapsulated within a Cu_9_X_3_ (X = Br or I) framework, which exhibits notable structural distinctions from previously reported copper halide clusters (Fig. 21c).^54^ Mechanistic studies indicate that the emissions of these copper halide clusters originate from the TADF process, resulting in high PLQYs. Among these copper halide clusters, the one with the tris(4-trifluoromethylphenyl)phosphine ligand exhibits the highest LY of 4924 photons per MeV and the lowest X-ray dose detection limit of 1.239 µGy s^−1^.

The XEL properties of copper iodide cluster scintillators are summarized in Table 2.

**Table 2:** A summary of the reported copper iodide cluster scintillators

Scintillator	Molecular formula	LY (photons per MeV)	PLQY (%)	*λ*_em_ (nm)	LOD (nGy s^−1^)	Spatial resolution for X-ray imaging (lp per mm)	Ref.
Cu_4_I_4_(3-pmbtd)_2_	C_49_H_46_Cl_2_Cu_4_I_4_N_8_O_10_	—	58	524	—	—	8
Cu_2_I_2_L(PPh_3_)_2_	C_60_H_65_Cu_2_I_2_N_2_P_3_	12 950	74.2	445	42.5	17.33	27
Cu_2_I_2_(L)_4_	C_74_H_28_Cl_36_Cu_2_I_2_N_4_	14 300	32	587	319	30.77	22
Tb–Cu_4_I_4_	C_21_H_19_Cu_2_I_2_N_4_O_7_Tb	29 379	70.5	547	45.2	12.66	10
Cu_4_I_4_[DDPACDBFDP]_2_	C_172_H_122_Cu_4_I_4_N_4_O_3_P_4_	—	95	508	77	12	16
Cu_4_I_4_(bzpy)_4_	C_96_H_88_N_8_Cu_8_I_8_	36 900	85	573	102.1	24.3	13
Cu_4_I_4_(*t*bpy)_4_	C_36_H_52_Cu_4_I_4_N_4_	30 800	85	623	137.2	21.2	13
Cu_4_I_4_(Ph_3_As)_3_	C_54_H_45_As_3_Cu_4_I_4_	15 000	100	565	18.1	—	32
Cu_4_I_4_(4-bzpy)_4_	C_96_H_88_N_8_Cu_8_I_8_	60 948	99.7	586	239 700	5	33
Cu_6_I_8_(4-bzpy)_2_	C_24_H_23_Cu_6_I_8_N_2_	5353	0.1	540	—	—	33
Cu_3_I_6_(4-bzpy)_3_	C_36_H_36_Cu_3_I_6_N_3_	7011	0.3	560	—	—	33
Cu_4_I_4_(3-ppy)_4_	C_44_H_36_Cu_4_I_4_N_4_	—	92.4	560	—	23.2	34
Cu_4_I_4_(4-ppy)_4_-1	C_44_H_36_Cu_4_I_4_N_4_	—	58.6	520	—	11.6	34
Cu_4_I_4_(4-ppy)_4_-2	C_44_H_36_Cu_4_I_4_N_4_	—	43.5	495	—	10.5	34
Cu_4_I_4_(3-pic)_4_	C_24_H_28_Cu_4_I_4_N_4_	60 617	89.25	585	910	13	35
(*R*/*S*-Cl-MBA)_4_Cu_4_I_4_	C_32_H_40_Cl_4_Cu_4_I_4_N_4_	—	46.62/45.76	666	—	—	36
(*R*/*S*-Br-MBA)_4_Cu_4_I_4_	C_32_H_40_Br_4_Cu_4_I_4_N_4_	4654	95.43/96.94	610	120 000	—	36
(*S*-Br-MBA)_4_Cu_4_I_4_	C_32_H_40_Br_4_Cu_4_I_4_N_4_	28 539	89.9	575	28.8	22	37
(MBA)_4_Cu_4_I_4_	C_32_H_44_Cu_4_I_4_N_4_	2128	35.7	596	73.6	—	37
Cu_4_I_4_(DPPPy)_2_	C_34_H_28_Cu_4_I_4_N_2_P_2_	29 000	92.7	525	88.2	—	38
Cu_2_I_2_(Dppy)(F-PPh_3_)_2_	C_53_H_38_Cu_2_I_2_F_6_NP_3_	175 300	91	533	43.1	27.6	12
Cu_2_Br_2_(Dppy)(F-PPh_3_)_2_	C_53_H_38_Cu_2_Br_2_F_6_NP_3_	102 400	83	553	71.5	23.2	12
Cu_4_I_4_py_4_	C_20_H_22_Cu_4_I_4_N_4_	—	92.4	565	55	20	9
Cu_4_I_4_(DBA)_4_	C_56_H_60_Cu_4_I_4_N_4_	12 842	94.9	584	—	5.0	14
Cu_2_I_2_(Pic_3_PO)_2_	C_36_H_36_I_2_Cu_2_N_6_O_2_P_2_·2(C_2_H_3_N)	—	30	485	—	—	11
Cu_2_Br_2_(Pic_3_PO)_2_	C_36_H_36_Br_2_Cu_2_N_6_O_2_P_2_·2(C_2_H_3_N)	—	44	507	—	—	11
Cu_2_Cl_2_(Pic_3_PO)_2_	C_36_H_36_Cl_2_Cu_2_N_6_O_2_P_2_·2(C_2_H_3_N)	—	54	530	—	—	11
Cu_2_I_2_(NP)_3_	C_51_H_42_Cu_2_I_2_N_3_P_3_	28 700	97.64	580	172.9	21.5	39
Cu_1_I_1_(MP)_3_	C_18_H_21_Cu_1_I_1_N_3_	19 297	98.26	496	1100	—	40
Cu_2_I_2_(MP)_4_	C_24_H_28_Cu_2_I_2_N_4_	28 329	97.49	504	630	—	40
Cu_4_I_4_(MP)_4_	C_48_H_56_Cu_8_I_8_N_8_	55 069	99.69	580	240	—	40
Cu_4_I_4_(PPh_2_Et)_4_	C_56_H_60_Cu_4_I_4_P_4_	74 500	48	610	50.3	30.8	42
Cu_4_I_6_(pr-ted)_2_	C_18_H_38_Cu_4_I_6_N_4_	—	97.1	535	22	20	15
Cu_4_I_6_(me-ted)_2_	C_14_H_30_Cu_4_I_6_N_4_	21 700	61.23	503	38.4	12.3	43
Cu_6_I_8_(bu-ted)_2_	C_18_H_38_Cu_4_I_6_N_4_	—	97.3	530	19.6	20	44
Cu_5_I_7_(bu-ted)_2_	C_20_H_42_Cu_5_I_7_N_4_·2CH_3_CN	11 300	23.4	600	377.4	12.5	45
Cu_5_I_7_(mepym-ted)_2_	C_20_H_34_Cu_5_I_7_N_4_	15 500	46.9	600	207.5	14.3	45
Cu_5_I_7_(bz-ted)_2_	C_26_H_42_Cu_5_I_7_N_4_	38 800	100	570	71.2	22.1	45
(*R*,*S*-2-mpip)_2_Cu_2_I_6_	C_10_H_28_Cu_2_I_6_N_4_	47 000	65.96	530	6.48	—	46
[N(C_3_H_7_)_4_]_2_^+^[Cu_2_Br_2_I_2_]^2−^	C_24_H_26_Cu_2_Br_2_I_2_N_2_	145 896	99.5	409	120.5	30	47
(C_8_H_20_N)_1_(C_12_H_28_N)_1_Cu_4_Br_6_	C_24_H_48_Cu_4_Br_6_I_2_N_2_	77 430	99	583	—	29.15	48
(C_6_H_18_N_2_)Cu_2_I_4_	C_6_H_18_Cu_2_I_4_N_4_	45 193	52.96	475	2280	22.3	49
(IPP)CuI_2_	C_42_H_42_Cu_2_I_4_P_2_	75 793.83	94.68	440/550	62.29	10.2	50
(IPP)_2_CuI_3_	C_42_H_42_CuI_3_P_2_	—	—	500	—	—	50
(C_12_H_24_O_6_)_2_Na_2_(H_2_O)_3_Cu_4_I_6_	C_24_H_52_Cu_4_I_6_Na_2_O_3_	—	100	536	—	24.8	52
(DIET)_3_Cu_3_Br_3_	C_15_H_36_N_6_S_3_Cu_3_Br_3_	20 000	69.2	515	189	11.71	53
Cu_9_I_3_(*^t^*BuC≡C)_9_(PCH_3_)_3_	C_99_H_90_Cu_9_F_27_I_3_P_3_	4924	43	580	1239	25.4	54

### 4.3 Copper cluster-based assembly scintillators

To improve the stability and XEL performance of copper clusters, some copper cluster-based assembly scintillators are developed. These copper cluster-based assembly materials typically contain copper iodide structural units, which can form copper cluster-based frameworks or copper cluster-based one-dimensional polymers.

**Fig. 22:**
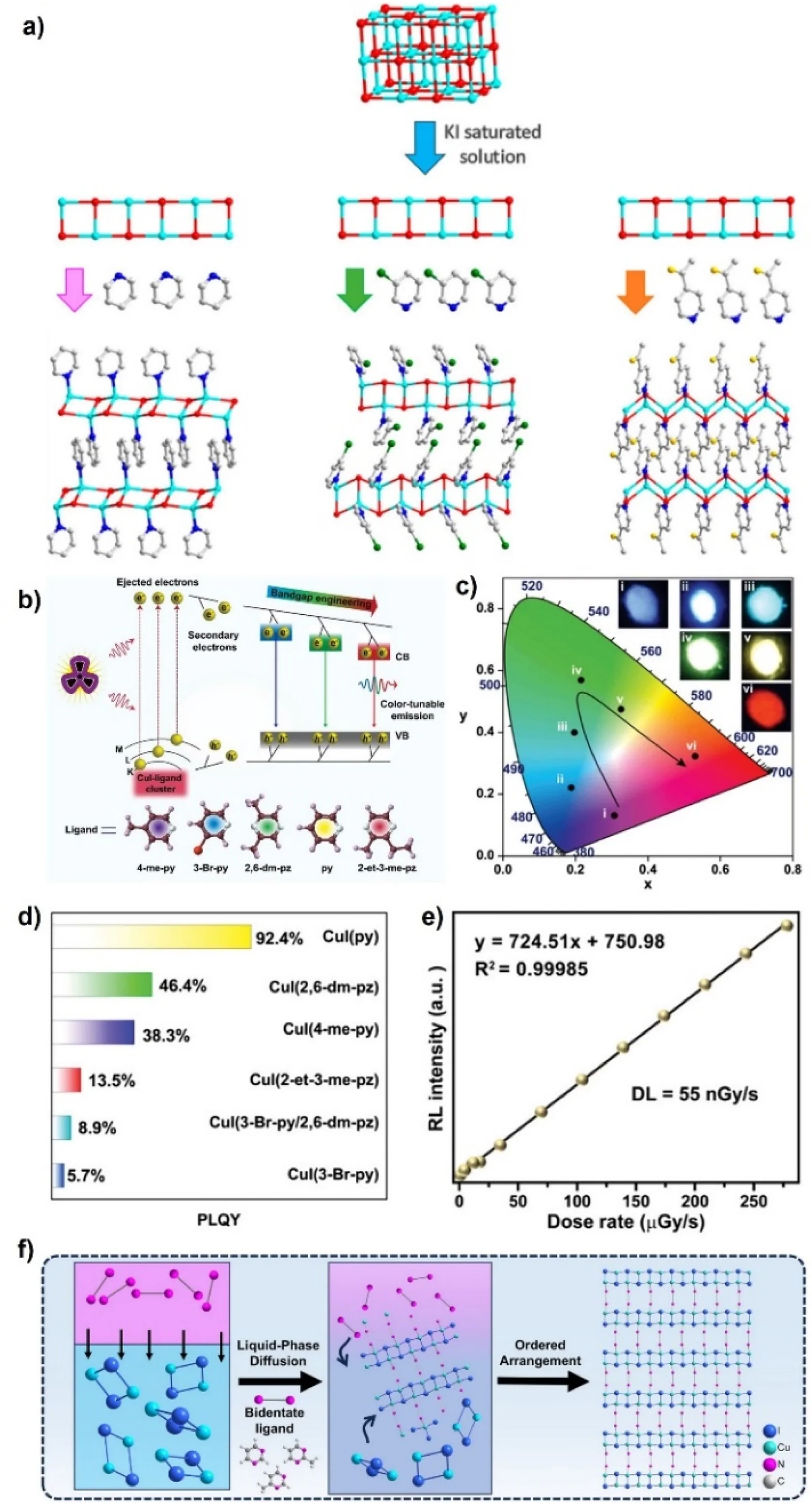
(a) Design and construction of the copper iodide cluster-based assembly materials.^55^ Reproduced from ref. 55 with permission from American Chemical Society, copyright 2014. (b) Schematic of scintillation emission tuning of the copper iodide cluster-based assembly materials. (c) A CIE chromaticity coordinate diagram of the XEL of the copper iodide cluster-based assembly materials. (d) PLQY of the copper iodide cluster-based assembly materials. (e) The linear dependence of the XEL intensity of CuI(py) as a function of dose rate.^9^ Reproduced from ref. 9 with permission from Wiley, copyright 2022. (f) Synthesis and structural description of CuI(4-Mepym).^56^ Reproduced from ref. 56 with permission from Wiley, copyright 2026.

For example, Li and coworkers reported a series of copper iodide cluster-based assembly materials including one-dimensional copper iodide–organic ligand hybrid structures in 2014 (Fig. 22a).^55^ These materials exhibit multiple emission colors and high PLQY and were successfully developed as high-performance scintillators by Liu and coworkers in 2023.^9^ As shown in Fig. 22b, six pyridine-containing ligands including pyridine (py), 4-methylpyridine (4-me-py), 3-pyridyl bromide (3-Br-py), 2,6-dimethylpyrazine (2,6-dm-pz), pyridine (py) and 2-ethyl-3-methylpyrazine (2-et-3-me-pz) are used in the preparation of the stable copper iodide clusters due to the fact that the N atom in the ligand can strongly coordinate with copper. In addition, the diversity of pyridine-contained ligands enables copper iodide cluster-based assembly materials with tailored bandgaps, permitting the generation of radioluminescence covering the whole visible region (Fig. 22c and d). Among these copper iodide cluster-based assembly materials, the one with pyridine ligands (CuI(py)) exhibits the highest PLQY of 92.4% and the lowest X-ray dose detection limit of 55 nGy s^−1^ (Fig. 22e). Zhao and coworkers proposed a ligand-confinement strategy that employs sterically hindered, electronically tuned pyrimidine-based bridging ligands to simultaneously boost XEL and accelerate decay dynamics in a 1D Cu–I cluster scintillator, CuI(4-Mepym) (Fig. 22f).^56^ Combined with high stability, good solution processability, and high LY, CuI(4-Mepym) breaks the conventional brightness–speed trade-off and enables high-resolution static imaging (>20 lp per mm), artifact-free dynamic X-ray imaging, and high-fidelity 3D tomography.

**Fig. 23:**
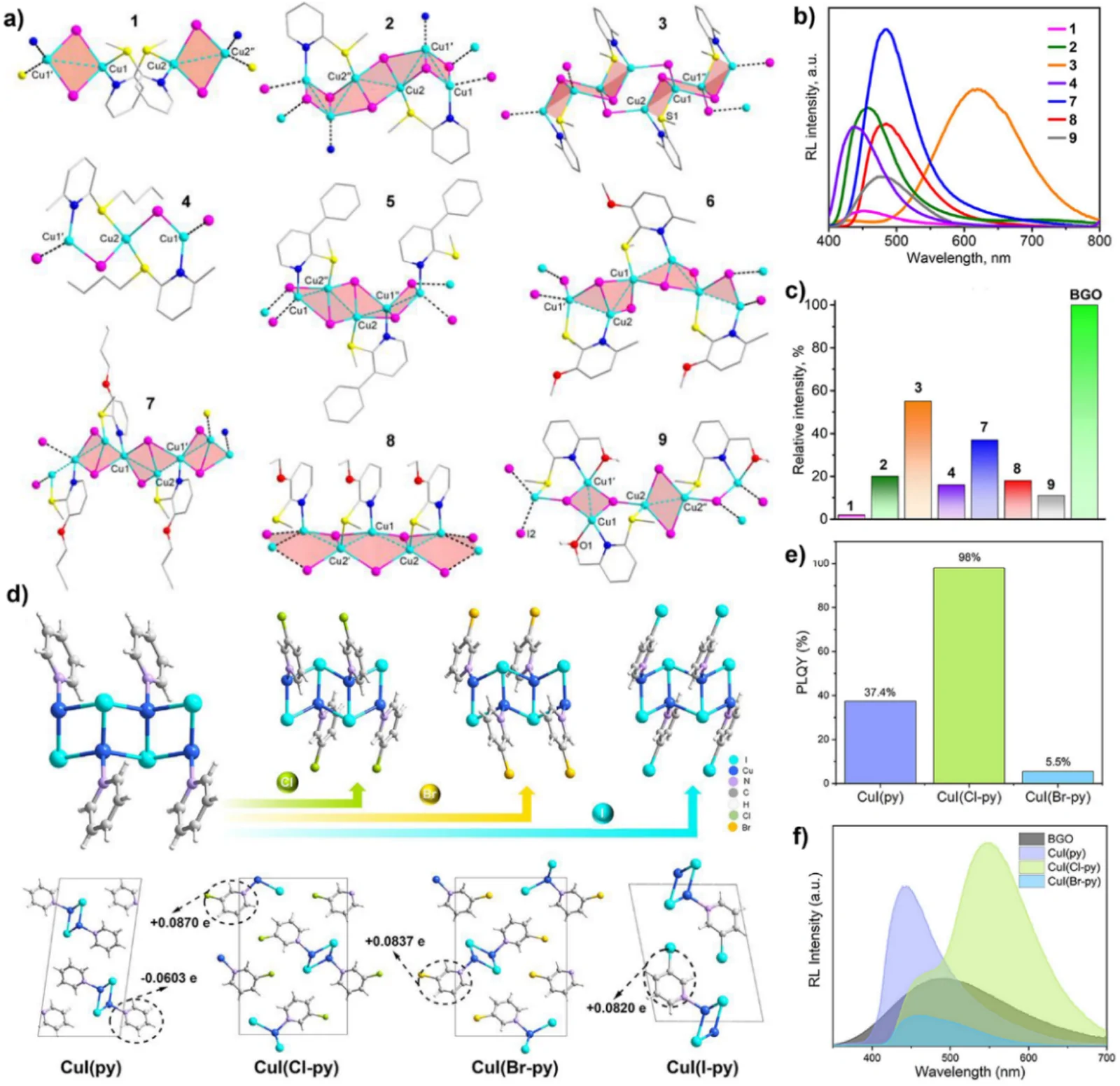
(a) Crystal structures of 1–9. (b) XEL spectra of 1–4 and 7–9 at 298 K. (c) XEL intensities of 1–4 and 7–9 against that of BGO at the same dose rate.^57^ Reproduced from ref. 57 with permission from Royal Society of Chemistry, copyright 2023. (d) Structure and Bader charge analysis of CuI(py), CuI(Cl-py), CuI(Br-py) and CuI(I-py). (e) PLQY of CuI(py), CuI(Cl-py) and CuI(Br-py). (f) XEL spectra of CuI(py), CuI(Cl-py), CuI(Br-py) and BGO.^58^ Reproduced from ref. 58 with permission from Elsevier, copyright 2024.

For example, Li's group reported a series of copper iodide-based assembly materials, which exhibit different radioluminescence properties.^57^ As illustrated in Fig. 23a, the one-dimensional chains of compound 1 are composed of repeating rhomboidal Cu_2_I_2_ units, which are interconnected through ligands in a 
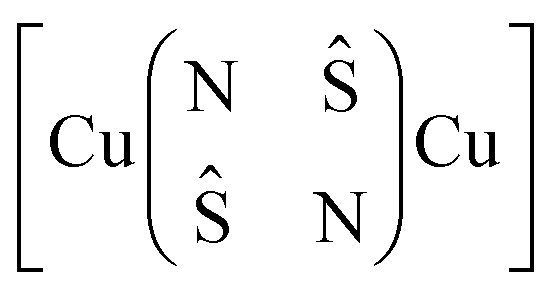
 bridging mode; compound 2 consists of double-stranded (Cu_2_I_2_)_*n*_ ribbons, with ligands decorating both sides and adjacent copper atoms bridging the structure in a µ_2_-N,S coordination mode; compound 3 features 
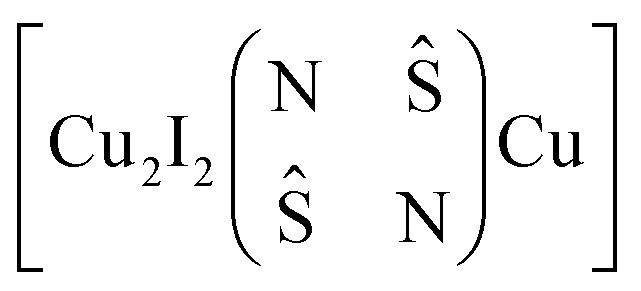
 chains constructed from alternating rhomboidal Cu_2_I_2_ units connected by two Cu–I “sticks”; the structure of compound 4 features zigzag (–Cu–I)_*n*_ chains, in which the “Cu–I–Cu” units are µ_2_-bridged by the N,S ligands; the scaffold of compound 5 is composed of double-stranded [Cu_2_I_2_]_*n*_ ribbons, in which the secondary building unit (Cu_4_I_4_L_2_) is made of two Cu_2_I_2_ butterfly-like motifs connected through Cu_2_–I_2_ edges, while the Cu1 and Cu2 atoms are µ_2_-N,S-bridged by the ligands; compounds 6 and 7 are built from the secondary building unit (Cu_4_I_4_L_2_), assembled into a one-dimensional chain featuring an “up and down” pattern; compound 8 features a double-stranded (Cu_2_I_2_)_*n*_ ribbon, forming a zigzag uniplanar array of repeating (–Cu2–Cu1–Cu2′–)_*n*_ units, with µ_2_-I atoms located on one side and µ_3_-I atoms on the opposite side; compound 9 contains two independent Cu_2_I_2_ rhomboids that are linked into curved one-dimensional chains *via* Cu_2_–I bonds. Among these compounds, compounds 1–4 and 7–9 reveal rather strong XEL at room temperature, whereas compounds 5 and 6 show no detectable XEL (Fig. 23b and c). This study highlights the structural diversity of copper iodide cluster-based assemblies and their potential for radioluminescence applications. Another example of one-dimensional copper iodide coordination polymers was reported by Omar's team in 2025.^58^ As shown in Fig. 23d and e, different halogen atoms were introduced to the ligands of the copper iodide coordination polymer CuI(py), resulting in CuI(Cl-py), CuI(Br-py) and CuI(I-py). The study shows that the incorporation of chlorine atoms into the ligands enhances structural rigidity, effectively suppressing non-radiative recombination and improving photoluminescent performance, resulting in an impressive PLQY of nearly 100% (Fig. 23f). CuI(Cl-py) demonstrates intense XEL intensity, low detection limits, and exceptional spatial resolution (Fig. 23g).

**Fig. 24:**
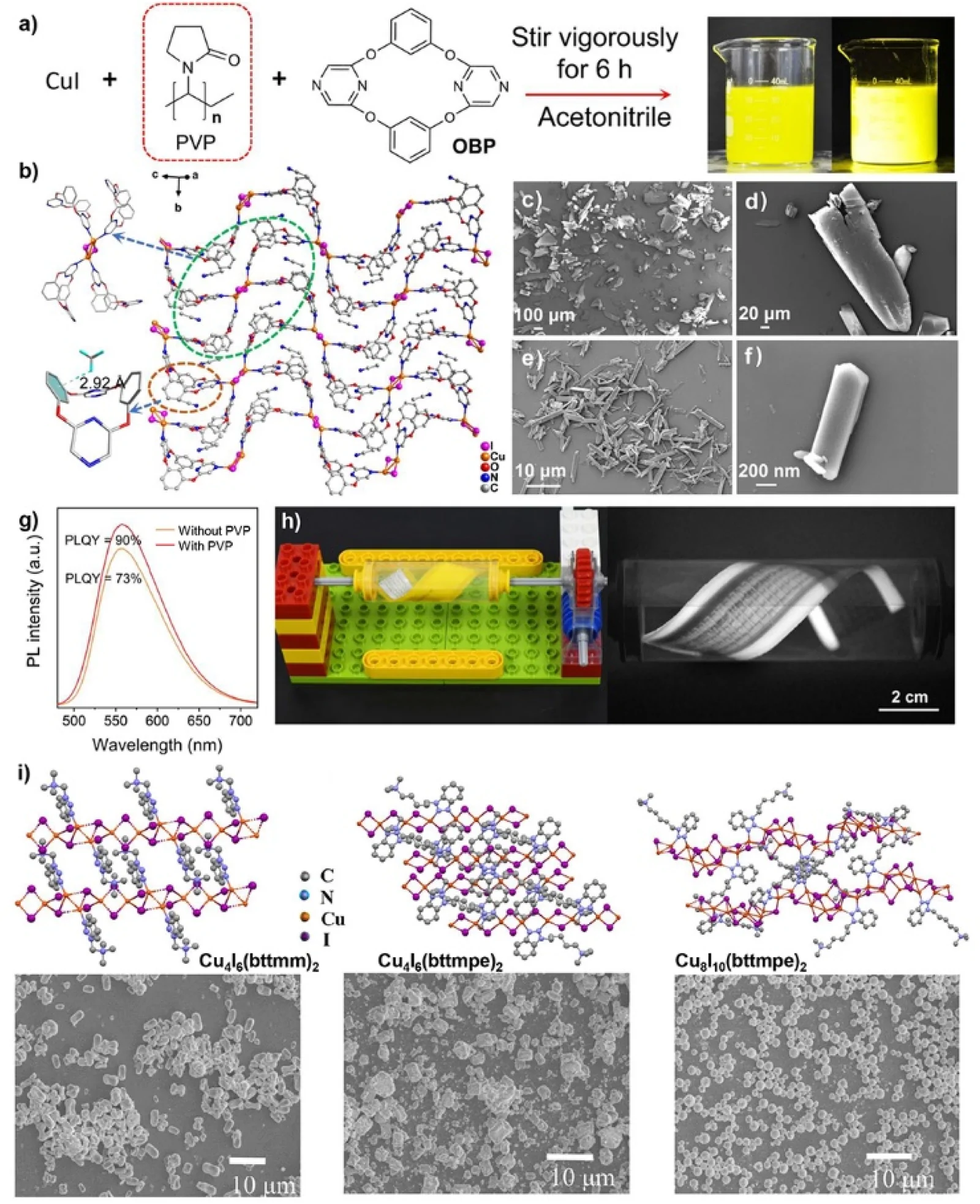
(a) Synthesis route of Cu_2_I_2_–OBP. (b) Crystal structure of Cu_2_I_2_–OBP. (c and d) SEM images of Cu_2_I_2_–OBP crystals without PVP in the synthesis process. (e and f) SEM images of Cu_2_I_2_–OBP crystals with PVP in the synthesis process. (g) Luminescence spectra of Cu_2_I_2_–OBP. (h) Dynamic flexible X-ray imaging device and real-time imaging photographs of a flexible OLED display with a Cu_2_I_2_–OBP scintillator screen.^17^ Reproduced from ref. 17 with permission from Wiley, copyright 2023. (i) Crystal structures and SEM images of Cu_4_I_6_(bttmm)_2_, Cu_4_I_6_(bttmpe)_2_ and Cu_8_I_10_(bttmpe)_2_ colloidal scintillators.^59^ Reproduced from ref. 59 with permission from Wiley, copyright 2024.

In 2023, Li's group reported a kind of copper iodide cluster-based MOF scintillator (Cu2I2–OBP) for high-performance dynamic X-ray flexible imaging (Fig. 24a–f).^17^ In this work, a macrocyclic bridging ligand of oxacalix[2]benzene[2]pyrazine (OBP) was synthesized, which exhibits typical AIE characteristics. The formation of a compact framework endows Cu_2_I_2_–OBP with high XEL efficiency and excellent chemical stability. Moreover, PVP was introduced during the *in situ* synthesis of Cu_2_I_2_–OBP, where it acts to disperse and stabilize the CuI units. The assembly of the ligand and CuI within PVP micelles leads to the formation of microcrystals with a well-defined rod-like morphology, thereby enhancing both the XEL performance and processability of the scintillator (Fig. 24g). By integrating Cu_2_I_2_–OBP with polydimethylsiloxane (PDMS), a flexible X-ray scintillator screen was fabricated, enabling high-performance dynamic X-ray flexible imaging of real objects (Fig. 24h). In 2025, Zhao's group reported a series of colloidal copper iodide cluster-based scintillators consisting of covalently bonded cation–anion pairs.^59^ As shown in Fig. 24i, three copper iodide cluster-based scintillators, Cu_4_I_6_(bttmm)_2_, Cu_4_I_6_(bttmpe)_2_ and Cu_8_I_10_(bttmpe)_2_ (bttmm = 1-(1*H*-benzo[*d*][1,2,3]triazol-1-yl)-*N*,*N*,*N*-trimethylmethanaminium, bttmpe = 5-(1*H*-benzo[*d*][1,2,3]triazol-1-yl)-*N*,*N*,*N*-tri-methylpentan-1-aminium), are synthesized. These compounds consist of three distinct types of one-dimensional anionic copper iodide (Cu_*m*_I_*m*+2_^2−^) chains, which are linked to small organic cationic ligands through either monodentate or bidentate coordination modes. Notably, the structures exhibiting bidentate coordination display significantly suppressed non-radiative decay. By incorporating strong ionic interactions between the coordination metal cluster core and ligands, the nucleation rate and growth of copper iodide cluster-based microcrystals can be effectively regulated through the use of ionic ligands and by leveraging electrostatic interactions between surfactants and ligands. Colloidal suspensions of copper iodide cluster-based scintillators were synthesized *via* confined growth in the presence of PVP. The resulting colloidal scintillators exhibit good radioluminescence performance and high sensitivity to X-ray dose rates. Compared with their crystalline counterparts, PVP-stabilized colloidal scintillators show enhanced compatibility with polymer matrices and reduced aggregation tendencies.

**Fig. 25:**
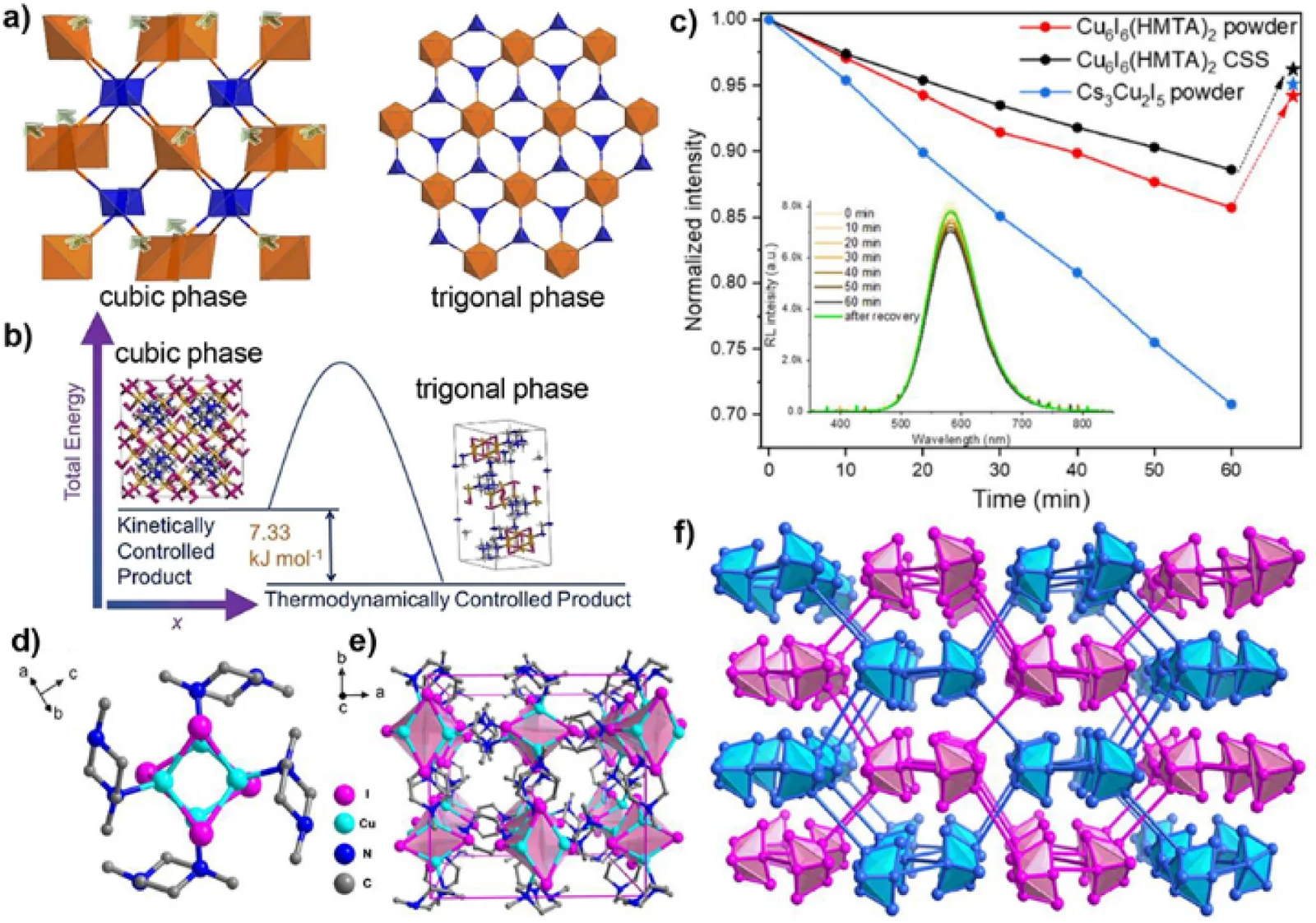
(a) Topological diagrams of cubic phase and trigonal phase Cu_6_I_6_(HMTA)_2_. (b) Diagram of energy *versus* reaction coordinate for the formations of cubic phase and trigonal phase Cu_6_I_6_(HMTA)_2_.^60^ Reproduced from ref. 60 with permission from American Chemical Society, copyright 2022. (c) Radiation stability of Cu_6_I_6_(HMTA)_2_ composite scintillation screen (black line) and thin-film scintillation screen (red line) in comparison with Cs_3_Cu_2_I_5_ (blue line) under continuous exposure of approximately 100 mGy s^−1^; the inset shows the corresponding XEL spectra during X-ray exposure.^61^ Reproduced from ref. 61 with permission from American Chemical Society, copyright 2025. (d) The structure of the Cu_4_I_4_ cluster in Cu_4_I_4_(dmp)_2_. (e) Structural packing diagram of Cu_4_I_4_(dmp)_2_. (f) The interlacing structure of Cu_4_I_4_(dmp)_2_.^62^ Reproduced from ref. 62 with permission from Royal Society of Chemistry, copyright 2025.

Ye and coworkers reported the copper iodide cluster-based MOF, Cu_6_I_6_(HMTA)_2_ (HMTA = hexamethylenetetramine), synthesized from readily available precursors through a scalable solution-based method. Cu_6_I_6_(HMTA)_2_ exhibits two polymorphic isomers, one is the cubic phase, and the other is the trigonal phase, featuring fully disordered and slightly disordered [Cu_6_I_6_] clusters, respectively (Fig. 25a and b).^60^ The scintillator properties of cubic-phase Cu_6_I_6_(HMTA)_2_ were investigated by Tarasov's group in 2025.^61^ The study demonstrates that Cu_6_I_6_(HMTA)_2_ exhibits a PLQY exceeding 95% and possesses exceptional thermal stability as well as radiation resistance. A Cu_6_I_6_(HMTA)_2_ composite scintillation screen was fabricated by embedding Cu_6_I_6_(HMTA)_2_ nanoparticles into an ethylene–vinyl acetate (EVA) matrix, achieving a high phosphor loading of 75 wt% while maintaining mechanical flexibility and uniformity. This composite scintillation screen exhibited a maximum LY of 63 500 photons per MeV and a spatial resolution of up to 18 lp per mm. Meanwhile, they also prepared a thin-film scintillator screen by depositing Cu_6_I_6_(HMTA)_2_ onto porous membranes, achieving an even higher spatial resolution of 24.5 lp per mm, although with reduced mechanical robustness. Remarkably, the Cu_6_I_6_(HMTA)_2_-based thin-film screen demonstrated superior radiation stability. As shown in Fig. 25c, under continuous X-ray exposure (approximately 100 mGy s^−1^), the luminescence intensity of the Cu_6_I_6_(HMTA)_2_-based thin-film screen decreased by only about 10%. After the cessation of exposure, the Cu_6_I_6_(HMTA)_2_-based thin-film screen exhibited self-healing behavior, with the luminescence intensity recovering to over 95% of its initial value within 12 h of dark storage. These results highlight the potential of Cu_6_I_6_(HMTA)_2_ as a high-performance scintillator for X-ray imaging applications. Another copper iodide cluster-based three-dimensional MOF, Cu_4_I_4_(dmp)_2_ (dmp = 1,4-dimethylpiperazine), was reported by Li's group.^62^ As shown in Fig. 25d, Cu_4_I_4_(dmp)_2_ shows a three-dimensional structure of Cu_4_I_4_ cubane clusters connected by dmp ligands, forming a tetrahedral propeller-like structure. Each pair of clusters shares a dmp ligand, interconnected through Cu–N bonds to generate a hexagonal ring structure, which is periodically arranged to constitute a three-dimensional framework (Fig. 25e). The extended three-dimensional network undergoes interpenetration, resulting in an interlaced structure with no guest molecules present within the pores (Fig. 25f). Cu_4_I_4_(dmp)_2_ demonstrates a good X-ray response, achieving an effective LY of 48 538 photons per MeV and a detection limit of 30.68 nGy s^−1^.

The XEL properties of copper cluster-based assembly scintillators are summarized in Table 3.

**Table 3:** A summary of the reported copper cluster-based assembly scintillators

Scintillator	Molecular formula	LY (photons per MeV)	PLQY (%)	*λ*_em_ (nm)	LOD (nGy s^−1^)	Spatial resolution for X-ray imaging (lp per mm)	Ref.
CuI(py)	C_5_H_4_CuI_2_N	—	92.4	564	55	20	9
CuI(Cl-py)	C_5_H_4_ClCuIN	—	98	540	250	16	58
CuI(4-Mepym)	C_5_H_6_Cu_2_I_2_N_2_	24 257	64.9	484	—	20	56
Cu_2_I_2_–OBP	Cu_2_I_2_C_40_H_24_N_8_O_8_	41 000	90	560	34.6	20	17
Cu_4_I_6_(bttmm)_2_	C_10_H_15_Cu_2_I_3_N_4_	—	41.2	545	—	12.55	59
Cu_4_I_6_(bttmpe)_2_	C_14_H_23_Cu_2_I_3_N_4_	—	60.8	560	—	15.88	59
Cu_8_I_10_(bttmpe)_2_	C_14_H_23_Cu_4_I_5_N_4_	—	70.5	575	—	17	59
Cu_6_I_6_(HMTA)_2_	C_6_H_12_N_4_Cu_3_I_3_	63 500	95	588	—	24.55	61
Cu_4_I_4_(dmp)_2_	Cu_4_I_4_C_12_H_28_N_4_	48 538	99.1	544	30.7	—	62

## 5. Structure–functionality correlation

Recently, the structure–functionality correlation of copper cluster scintillators has become the core issue of research in this field. Some copper cluster scintillators with novel scintillation properties such as X-ray excited circularly polarized luminescence (XECPL), afterglow radioluminescence, temperature-inert radioluminescence and negative thermal quenching (NTQ) have been successively reported. Copper cluster scintillators have also been processed into various forms, such as nanoglasses, hydrogels, *etc.*, exhibiting multiple properties and application potential.

**Fig. 26:**
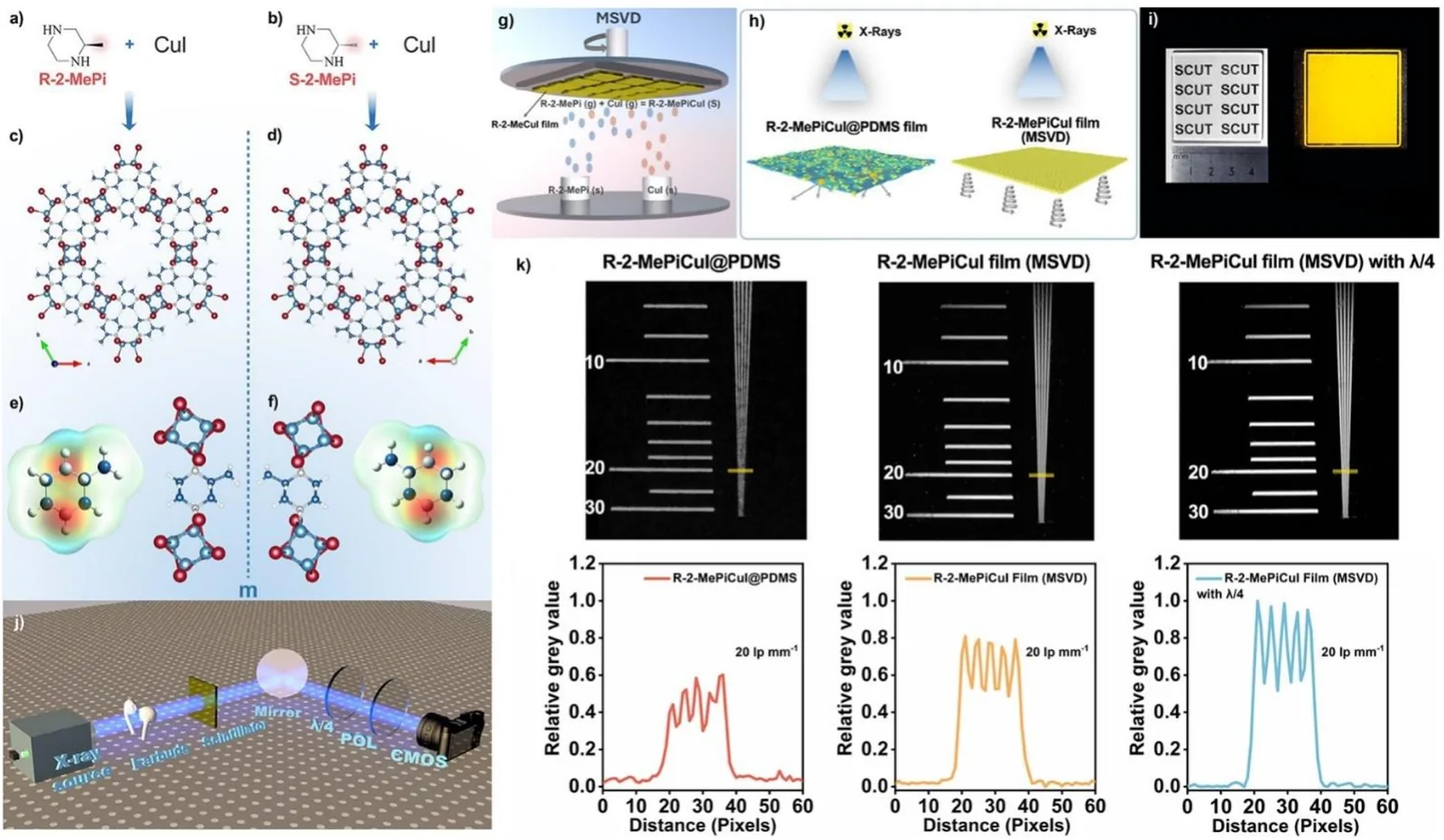
The chemical structures of (a) *R*-2-MePi and (b) *S*-2-MePi. Crystal structures of (c) *R*-2-MePiCuI and (d) *S*-2-MePiCuI. Electrostatic potential maps and units composed of Cu_4_I_4_ clusters and organic molecules of (e) *R*-2-MePiCuI and (f) *S*-2-MePiCuI. (g) Schematic illustration of the MSVD process, in which CuI and *R*-2-MePi are used as the evaporation sources. (h) Comparative images of the *R*-2MePiCuI@PDMS film and *R*-2MePiCuI film (MSVD) under X-ray irradiation. (i) Schematic illustration of the X-ray imaging optical path for the *R*-2MePiCuI film (MSVD). (j) X-ray imaging of the line-pair resolution card and corresponding gray value contour of the line pair. (k) X-ray imaging of the line-pair resolution card and the gray value contour of the line pair extracted from the *R*-2-MePiCuI@PDMS film, *R*-2-MePiCuI (MSVD) film and *R*-2-MePiCuI (MSVD) film with a *λ*/4 waveplate.^63^ Reproduced from ref. 63 with permission from Wiley, copyright 2025.

In 2025, Xia's group reported a pair of chiral hybrid copper iodide scintillators, *R*-2-MePiCuI and *S*-2-MePiCuI (*R*/*S*-2-MePi = *R*/*S*-2-methylpiperazine). *R*-2-MePiCuI and *S*-2-MePiCuI exhibit near-unity PLQYs of 99.08% ± 0.21% and 98.38% ± 0.19%, respectively, along with pronounced CPL signals and dissymmetry factors (*g*_lum_) of +0.82 × 10^−2^ and −0.67 × 10^−2^.^63^ As shown in Fig. 26a–f, the Cu_4_I_4_ units are coordinated by the two nitrogen atoms of the *R*/*S*-2-MePi ligands, leading to the formation of a three-dimensional network structure. Furthermore, high-quality chiral scintillator films were fabricated using *R*-2-MePiCuI and *S*-2-MePiCuI through a multisource vacuum deposition (MSVD) technique, enabling the formation of dense and uniform scintillating layers (Fig. 26g–i). They employed a custom-built optical setup in which a quarter-wave plate (*λ*/4 waveplate) and a linear polarizer were integrated into the conventional X-ray imaging pathway (Fig. 26j). This configuration ensures that the light reaching the complementary metal–oxide–semiconductor (CMOS) detector is polarized in a single direction, thereby significantly reducing the potential for light scattering and signal crosstalk.^64^ The imaging resolution of three types of films (*R*-2-MePiCuI@PDMS, which was fabricated with *R*-2-MePiCuI and PDMS; *R*-2-MePiCuI film (MSVD), which was prepared by the multi-source vacuum deposition (MSVD) method; *R*-2-MePiCuI film (MSVD) with *λ*/4, which was integrated into an optical system equipped with a *λ*/4 waveplate and a linear polarizer) was compared. As shown in Fig. 26k, the *R*-2-MePiCuI film (MSVD) with *λ*/4 demonstrates superior performance in both relative grey value and intensity resolution. This study demonstrates the potential of chiral copper iodide cluster scintillators exhibiting XECPL for application in high-resolution X-ray imaging. In 2026, Huang and coworkers used chiral diphosphines and trace triphenylphosphines (TPPs) to govern the coordination and nonerosive doping of the binuclear Cu(i) clusters; the resulting microcrystal exhibits a high spatial resolution of 24.1 lp per mm.^65^

**Fig. 27:**
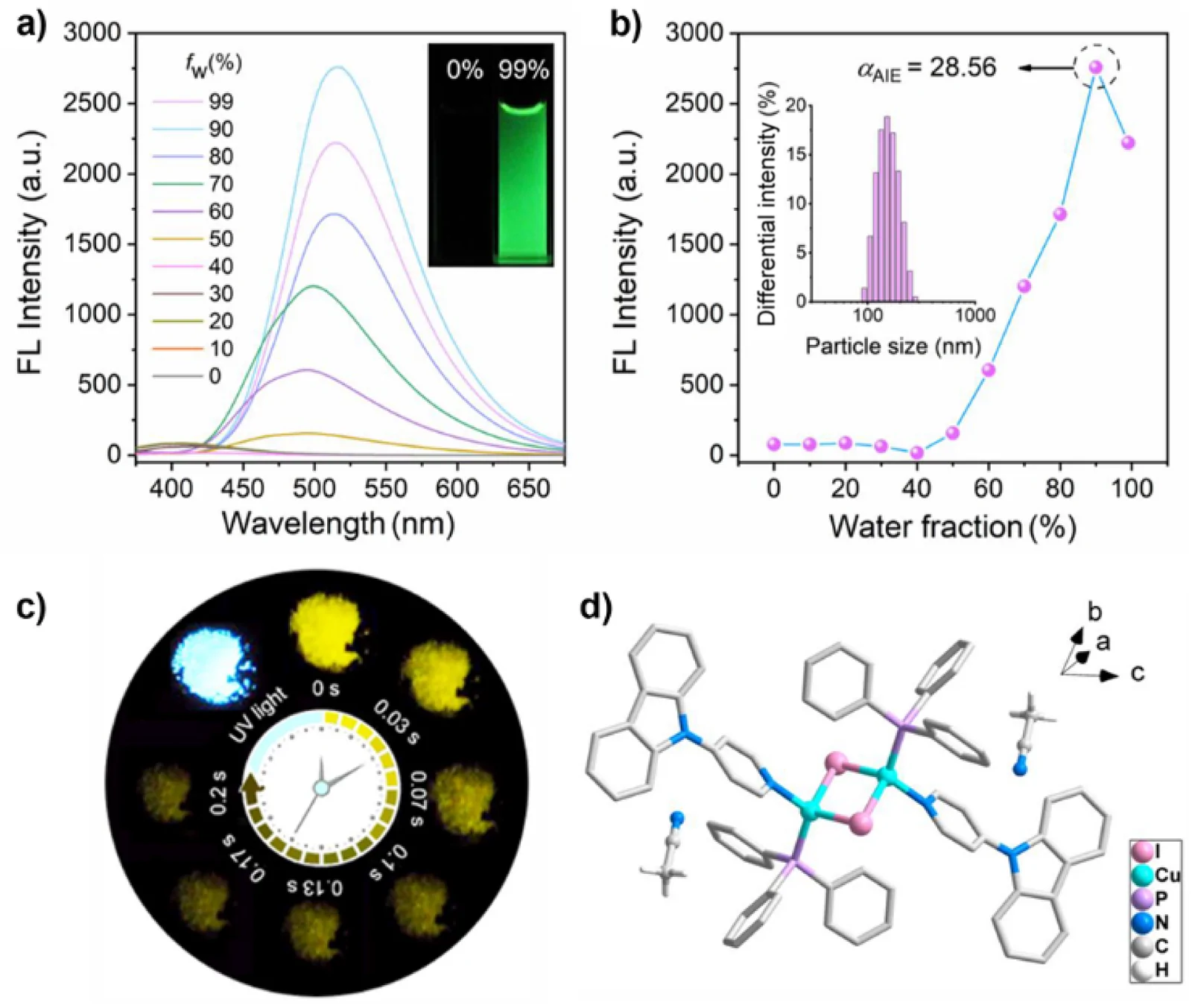
(a) Luminescence spectra of Cu_2_I_2_–CH_3_CN in different H_2_O/DMSO mixtures. Inset: Photographs of Cu_2_I_2_–CH_3_CN in H_2_O/DMSO mixtures (*f*_w_ = 0% and 99%) under UV light. (b) Luminescence intensity of Cu_2_I_2_–CH_3_CN at 516 nm as a function of *f*_w_. Inset: The DLS result of Cu_2_I_2_–CH_3_CN in H_2_O/DMSO (*f*_w_ = 99%). (c) Luminescence photograph (blue emission) and afterglow photographs (yellow emission) after UV light was removed. (d) Crystal structure of Cu_2_I_2_–CH_3_CN.^21^ Reproduced from ref. 21 with permission from Wiley, copyright 2025.

In 2025, Li's group reported a copper iodide cluster of Cu_2_I_2_–CH_3_CN exhibiting unique afterglow emission with a lifetime of 133.5 ms, ultrahigh PLQY (90.1% in the solid state) and AIE behavior (Fig. 27a–c).^21^Cu_2_I_2_–CH_3_CN can be easily prepared from 9-(pyridin-4-yl)-9*H*-carbazole and PPh_3_ ligands with CuI under solvothermal conditions (Fig. 27d). The crystal of Cu_2_I_2_–CH_3_CN belongs to the *P*1̄ space group in the triclinic crystal system. The Cu_2_I_2_ core of the cluster forms a planar quadrilateral structure consisting of two copper atoms and two iodide ions. Each copper atom is coordinated to one Cz-Py ligand and one PPh_3_ ligand, respectively, resulting in a zero-dimensional structure. Each cluster is surrounded by two CH_3_CN molecules positioned between the Cz-Py and PPh_3_ ligands, resulting in a centrosymmetric overall structure. Mechanistic studies demonstrate that TADF and long-lifetime phosphorescence occur simultaneously in Cu_2_I_2_–CH_3_CN. The unique photoluminescence properties of Cu_2_I_2_–CH_3_CN are attributed to the large spin–orbit coupling (SOC) and long-term rigidity of the crystal. The high PLQY, TADF characteristics, and heavy-atom composition of Cu_2_I_2_–CH_3_CN endow it with excellent XEL properties. A flexible scintillator screen based on Cu_2_I_2_–CH_3_CN was successfully fabricated and employed for X-ray imaging, achieving a spatial resolution of 23.6 lp per mm.

**Fig. 28:**
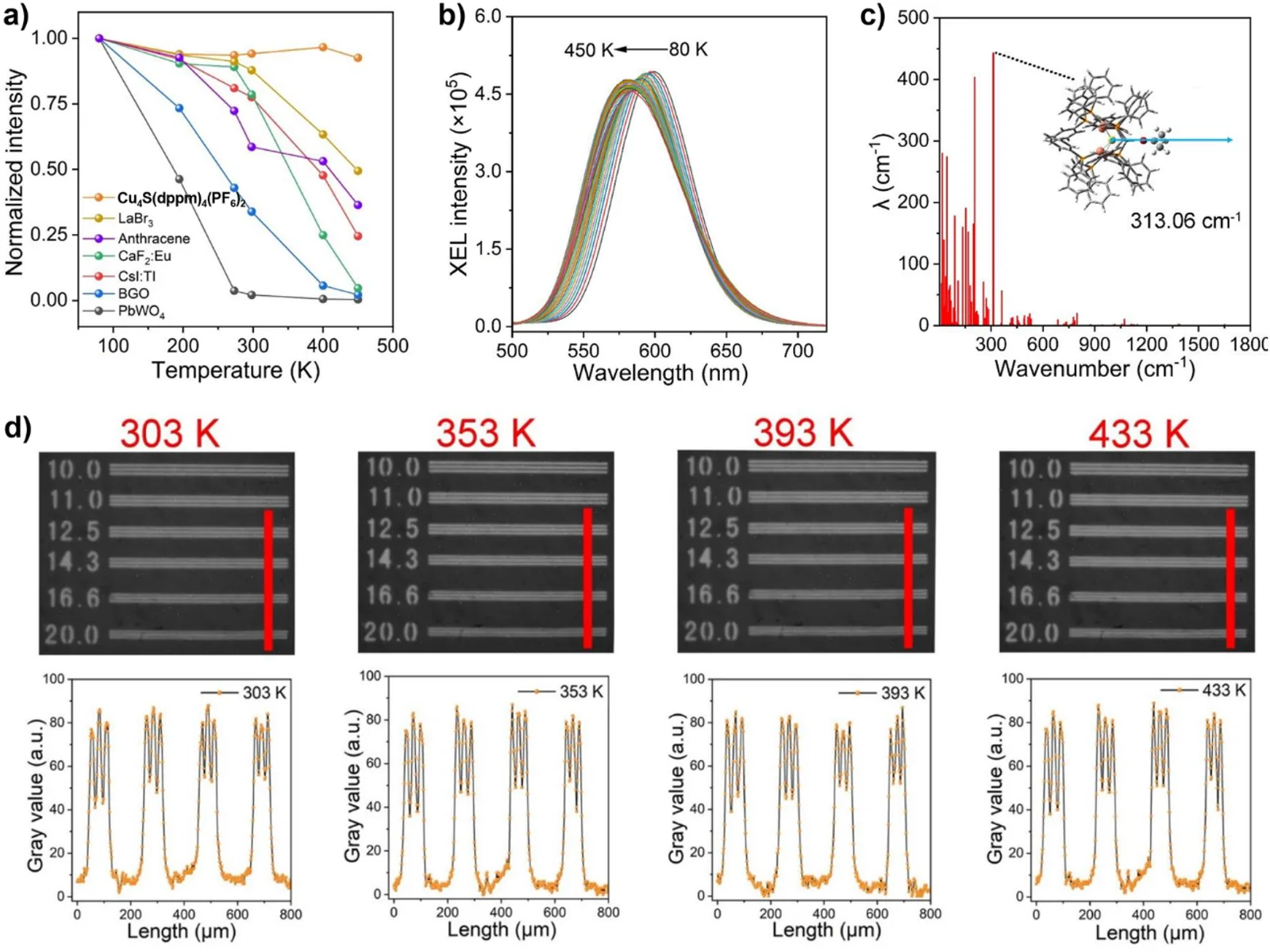
(a) Difference in the XEL intensity of Cu_4_S(dppm)_4_(PF_6_)_2_ compared with that of commercial scintillators over the temperature variation range of 80–450 K. (b) Temperature-dependent XEL spectra of Cu_4_S(dppm)_4_(PF_6_)_2_. (c) Reorganization energy components as a function of frequency at the PBE0/def2-SVP level in the Cu_4_S(dppm)_4_(PF_6_)_2_ system. Displacement vectors and calculated frequencies of the main vibrational mode of Cu_4_S(dppm)_4_(PF_6_)_2_ (labeled with blue arrows). (d) Reorganization energy components as a function of frequency at the PBE0/def2-SVP level in the Cu_4_S(dppm)_4_(PF_6_)_2_ system. Displacement vectors and calculated frequencies of the main vibrational mode of Cu_4_S(dppm)_4_(PF_6_)_2_ (labeled with blue arrows).^23^ Reproduced from ref. 23 with permission from Wiley, copyright 2025.

Similar to conventional luminogenic materials, the emission performance of scintillators is typically sensitive to ambient temperature. Increasing the temperature results in a significant decrease in radioluminescence intensity. In 2025, Li's group reported a temperature-inert copper cluster scintillator, Cu_4_S(dppm)_4_(PF_6_)_2_, whose structure was introduced in Section 4.1 (Fig. 10b and c).^23^Cu_4_S(dppm)_4_(PF_6_)_2_ demonstrates strong radioluminescence under X-ray excitation at room temperature and maintains remarkable emission stability across an extensive temperature range from 80 K to 450 K (Fig. 28a and b). Mechanistic studies revealed that Cu_4_S(dppm)_4_(PF_6_)_2_ has typical TADF characteristics. The restricted geometric relaxation during the spin-flip process, attributed to pronounced C–H⋯π interactions among the clusters, results in an extremely low reorganization energy (Fig. 28c). This induced intersystem crossing within the Marcus inverted region, thereby enabling temperature-independent radioluminescence behaviour. More importantly, the Cu_4_S(dppm)_4_(PF_6_)_2_ cluster exhibits outstanding stability against moisture, oxygen, and thermal stress, making it suitable for fabricating flexible scintillation screens using PDMS as a matrix. Based on these features, temperature-independent high-resolution X-ray imaging was successfully demonstrated using the Cu_4_S(dppm)_4_(PF_6_)_2_ scintillator screen achieving a spatial resolution consistently exceeding 20 lp per mm across varying temperatures (Fig. 28d).

**Fig. 29:**
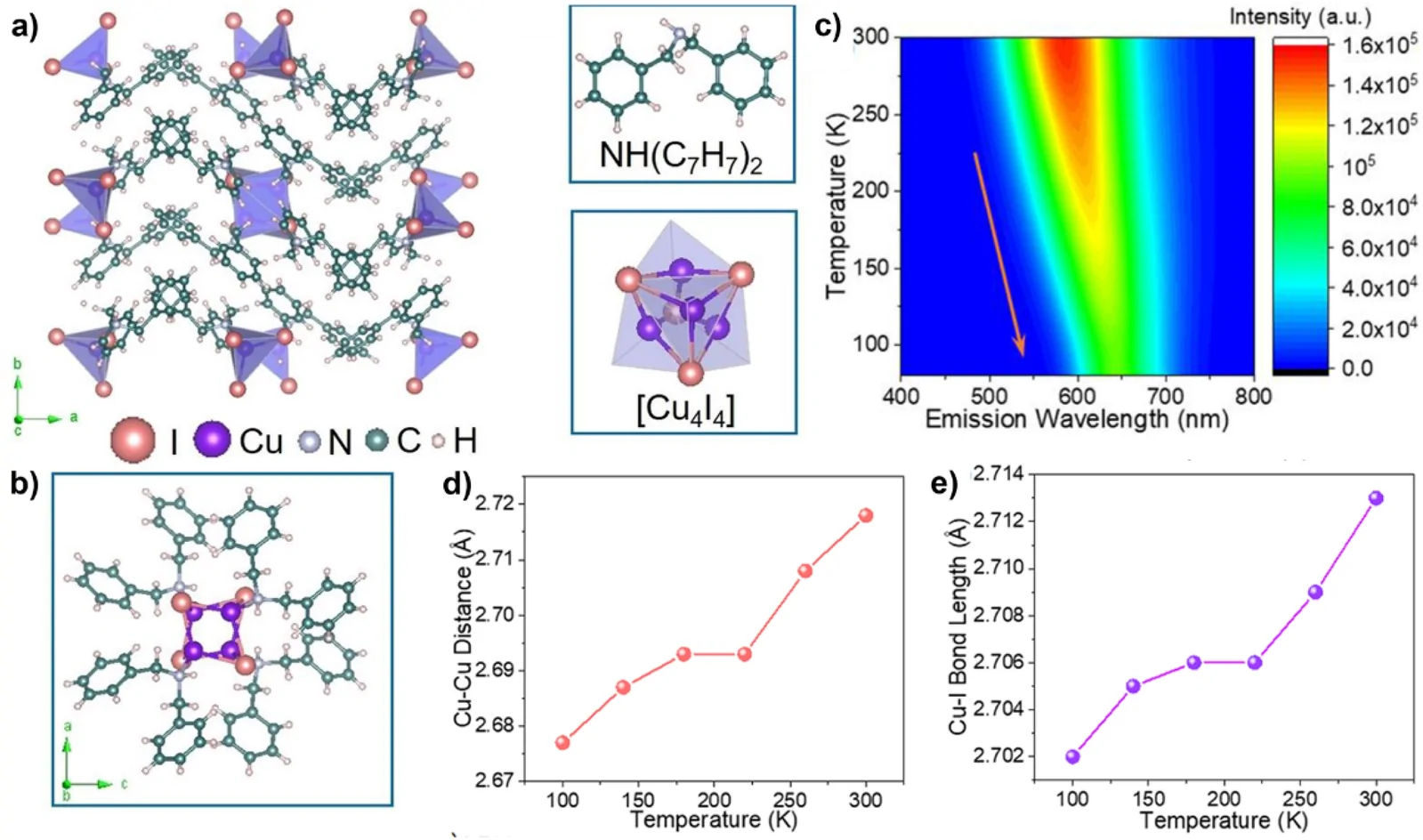
(a) Crystal structure and (b) molecular structure of (DBA)_4_Cu_4_I_4_. (c) Pseudo color map of temperature-dependent photoluminescence spectra of (DBA)_4_Cu_4_I_4_. (d) Temperature-dependent Cu–Cu distances of (DBA)_4_Cu_4_I_4_. (e) Temperature-dependent Cu–I bond lengths of (DBA)_4_Cu_4_I_4_.^14^ Reproduced from ref. 14 with permission from Wiley, copyright 2023.

Beside the temperature-inert coinage metal cluster scintillator, those with NTQ emission have also attracted widespread attention from researchers. In 2023, Sun's group reported a copper iodide cluster scintillator, (DBA)_4_Cu_4_I_4_ (DBA = dibenzylamine), with NTQ phosphorescence (Fig. 29a and b).^14^ The synergistic interplay between excited-state structural reorganization and metallophilic interactions in (DBA)_4_Cu_4_I_4_ gives rise to highly efficient triplet yellow-orange emission with a PLQY exceeding 94.9%. Meanwhile, a pronounced NTQ effect was observed in (DBA)_4_Cu_4_I_4_ over the temperature range from 80 K to 300 K (Fig. 29c). Mechanistic studies reveal that the NTQ effect in (DBA)_4_Cu_4_I_4_ originates from a phonon-assisted de-trapping process of excitons. The trapping–de-trapping process is temperature-dependent, whereby trapped charge carriers are thermally released to the emissive state and subsequently undergo radiative recombination, resulting in enhanced emission intensity at elevated temperatures. The authors propose that photoexcitation induces electron-structure reorganization, predominantly driven by copper d–sp transitions, which in turn causes structural distortion within the Cu_4_I_4_ core and leads to the formation of shallow defects. Subsequently, a portion of excitons that populate the CC triplet state *via* the ISC process are captured by these photoinduced shallow defect states. With phonon assistance, these excitons are released from the trap states and return to the triplet emissive level, where radiative recombination occurs and photons are emitted, giving rise to the NTQ effect. The process involving phonon-assisted exciton de-trapping and subsequent return to the emissive energy level is highly temperature-dependent, with its efficiency decreasing as temperature decreases. At the same time, the Cu–Cu distances and Cu–I bond lengths decrease as temperature decreases (Fig. 29d and e). The repulsive force from metallophilic interactions between copper ions and the distortion of the Cu_4_I_4_ cluster increases, leading to a continuous decrease in photoluminescence efficiency and a red-shifted emission spectrum. As a copper cluster scintillator, the (DBA)_4_Cu_4_I_4_ wafer demonstrates a LY higher than 10^4^ photons per MeV and a spatial resolution of 5.0 lp per mm.

**Fig. 30:**
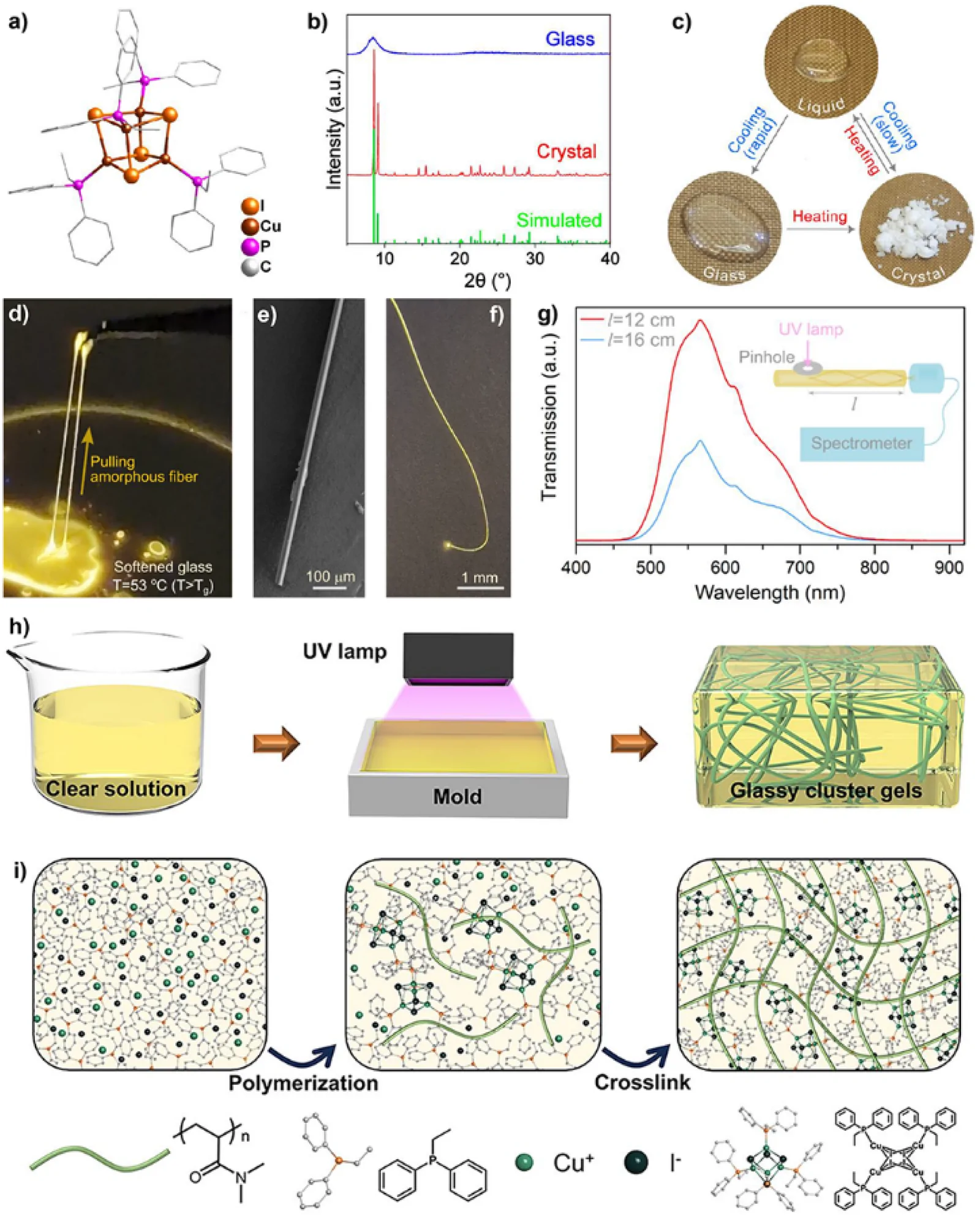
(a) Crystal structure of Cu_4_I_4_(PPh_2_Et)_4_. (b) PXRD patterns of the Cu_4_I_4_(PPh_2_Et)_4_ crystal and glass. (c) Schematic illustration of the phase transitions between crystal, liquid, and glass of Cu_4_I_4_(PPh_2_Et)_4_. (d) Producing uniform nanocluster glass fibers from the amorphous phase by pulling with tweezers at a temperature 53 °C. (e) SEM and (f) optical images of a 20 cm long nanocluster glass fiber with 45 µm diameter. (g) Measured optical transmission through a glass fiber with 200 µm diameter and 30 cm total length.^42^ Reproduced from ref. 42 with permission from American Chemical Society, copyright 2024. (h) Schematic diagram of the cluster gel synthesized *in situ* by photopolymerization. (i) Schematic representation of the microstructure from mixed solution to gradual polymerization to completion of polymerization.^66^ Reproduced from ref. 66 with permission from Elsevier, copyright 2025.

In 2024, Osman and coworkers demonstrated that Cu_4_I_4_(PR_3_)_4_ nanoclusters stabilized by phosphine ligands (PR_3_) can form robust melt-quenched glasses under ambient conditions, exhibiting reversible crystal–liquid–glass transitions (Fig. 30a–c).^42^ Protective phosphine ligands play a critical role in influencing the glass formation mechanism and modulating the physical properties of the resulting glasses. The hybrid glass of Cu_4_I_4_(PPh_2_Et)_4_ exhibits outstanding optical properties, including greater than 90% transmittance across both the visible and near-infrared regions, negligible self-absorption, near-unity PLQY, and high LY. This highly transparent glass enables high-performance X-ray imaging and low-loss waveguiding in fibers (Fig. 30d–g). Subsequently, Xia's group developed a rapid and *in situ* synthesis strategy to fabricate large-area scintillator films composed of Cu_4_I_4_(PPh_2_Et)_4_ glassy cluster gels for high-resolution X-ray imaging. They replaced the liquid dispersion medium in conventional gels with luminescent glassy Cu_4_I_4_(PPh_2_Et)_4_ clusters (Fig. 30h).^66^ The glassy copper-based cluster structure within the gel enables light transmission with negligible scattering, thereby maintaining high transparency. The glassy Cu_4_I_4_(PPh_2_Et)_4_ cluster gels are fabricated through *in situ* chemical synthesis *via* photopolymerization, with a reaction time on the order of seconds, enabling rapid and cost-effective production of large-area scintillator materials (Fig. 30i). The as-prepared glassy copper-based cluster gel scintillators can fully absorb X-rays and convert X-rays into visible light emission, enabling high-resolution X-ray imaging, with spatial resolutions of 29.3 lp per mm and 21.2 lp per mm achieved for scintillator films of 1 mm and 3 mm thickness, respectively. The synthesis method is generalizable and holds promise for extension to the *in situ* synthesis of other glassy cluster-structured composites.

**Fig. 31:**
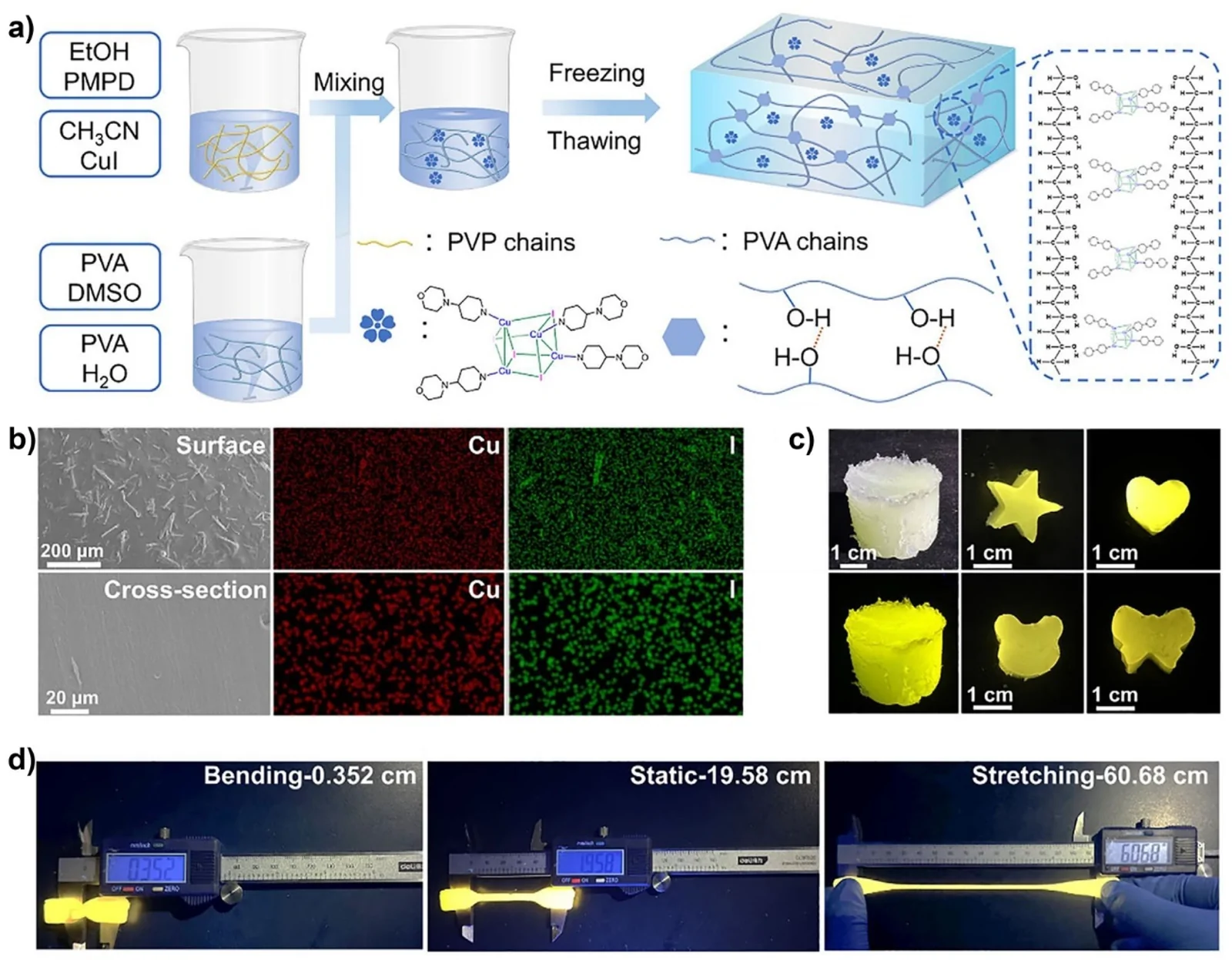
(a) The preparation method and formation mechanism of the Cu_4_I_4_(4-PMPD)_4_ scintillator hydrogel. (b) The SEM images and EDS elemental mapping (copper and iodine) of the Cu_4_I_4_(4-PMPD)_4_ scintillator hydrogel. (c) Images of the Cu_4_I_4_(4-PMPD)_4_ scintillator hydrogel under visible light and UV light. (d) Images of the stretched and folded Cu_4_I_4_(4-PMPD)_4_ scintillator hydrogel.^67^ Reproduced from ref. 67 with permission from Wiley, copyright 2025.

In 2025, a high-performance scintillator hydrogel based on Cu_4_I_4_(4-PMPD)_4_ (4-PMPD = (4-(piperidin-4-yl)morpholine)) cluster was developed by Xia and Lei's group.^67^ As shown in Fig. 31a, the micron-sized Cu_4_I_4_(4-PMPD)_4_ microcrystals, synthesized *via* PVP-mediated growth control, were dispersed into a polyvinyl alcohol (PVA)–H_2_O/DMSO solution and subjected to a freeze–thaw crosslinking process to form a PVA network structure, in which the Cu_4_I_4_(4-PMPD)_4_ microcrystals are embedded and linked to PVA chains through hydrogen bonding. SEM images reveal that Cu_4_I_4_(4-PMPD)_4_ microcrystals exhibit a certain degree of surface self-crystallization on the gel, while no large crystals are observed in the cross-section (Fig. 31b). This phenomenon can be attributed to the gel network, which constrains the growth of Cu_4_I_4_(4-PMPD)_4_ crystals. EDS analysis indicates a uniform distribution of copper and iodine elements, confirming the homogeneous dispersion of Cu_4_I_4_(4-PMPD)_4_ microcrystals within the hydrogel matrix. In this manner, they successfully prepared large-volume cylindrical scintillator hydrogels and fabricated them into various shapes (Fig. 31c). Owing to its high transmittance, the scintillator hydrogel film achieves an X-ray imaging resolution of 14 lp per mm. Furthermore, the synergistic effects of hydrogen bonding and coordination bonding endow the hydrogel scintillator with excellent plasticity and flexible stretchability (Fig. 31d).

## 6. Conclusions and future perspectives

In summary, copper clusters represent an emerging and highly promising class of scintillation materials for X-ray detection and imaging applications, attributed to their favorable photophysical properties. In this review, we comprehensively summarized the luminescence mechanisms and design strategies employed to tailor the XEL of copper clusters, copper halide clusters and copper cluster-based assembly materials. The novel applications derived from the as-obtained copper cluster scintillators are also summarized, along with the underlying structure–functionality correlations.

In recent years, copper cluster-based scintillators have achieved substantial progress, demonstrating strong potential for enabling high-performance radiation detection. Nevertheless, several critical challenges remain to be addressed. One of the most pressing issues is the difficulty in obtaining high-quality single crystal copper cluster materials. Most copper clusters are molecular crystals, and the inherently weak intermolecular forces hinder the growth of high-quality single crystals. Meanwhile, the processibility and stability of copper cluster scintillators are still limited and need to be urgently improved. These factors directly influence the practical feasibility and broader application of copper cluster scintillators. Another key challenge lies in the incomplete understanding of their scintillation mechanisms, which necessitates more in-depth investigation. Due to their structural complexity relative to conventional scintillators, the radioluminescence processes in copper cluster-based systems are highly complicated. Multiple exciton transition pathways can be modulated by various factors, including molecular packing modes, temperature, matrix materials, and excitation sources. A thorough understanding of the underlying radioluminescence mechanisms is essential for guiding the rational design of high-performance copper cluster scintillators. Recent advances in transient spectroscopy techniques and theoretical computational methods are expected to accelerate progress in this area. Finally, the application field of copper cluster scintillators requires further expansion. Despite the rapid emergence of numerous new materials, their current applications remain predominantly confined to X-ray imaging. Substantial challenges and opportunities coexist in extending their use to high-energy particle detection, neutron imaging, and X-ray-activated therapy.

## Author contributions

Y. Y. Li, J. L. Duan, Y. J. Kong, Q. C. Peng, and K. Li: investigation, conceptualization, writing – original draft, visualization, funding acquisition and writing – review & editing; S. Q. Zang: conceptualization, funding acquisition and supervision.

## Conflicts of interest

The authors declare no competing interests.

## Data Availability

No primary research results, software or code have been included and no new data were generated or analysed as part of this review.
